# A Recent Class of Chemosensory Neurons Developed in Mouse and Rat

**DOI:** 10.1371/journal.pone.0024462

**Published:** 2011-09-09

**Authors:** Lucia Silvotti, Eleonora Cavalca, Rita Gatti, Riccardo Percudani, Roberto Tirindelli

**Affiliations:** 1 Department of Neuroscience, University of Parma, Parma, Italy; 2 Italian Institute of Technology (IIT, BCSMC), University of Parma, Parma, Italy; 3 Department of Experimental Medicine, University of Parma, Parma, Italy; 4 Department of Biochemistry, University of Parma, Parma, Italy; Dalhousie University, Canada

## Abstract

In most animal species, the vomeronasal organ ensures the individual recognition of conspecifics, a prerequisite for a successful reproduction. The vomeronasal organ expresses several receptors for pheromone detection. Mouse vomeronasal type-2 receptors (V2Rs) are restricted to the basal neurons of this organ and organized in four families. Family-A, B and D (family ABD) V2Rs are expressed monogenically (one receptor per neuron) and coexpress with either Vmn2r1 or Vmn2r2, two members of family-C V2Rs. Thus, basal neurons are characterized by specific combinations of two V2Rs. To investigate this issue, we raised antibodies against all family-C V2Rs and analyzed their expression pattern. We found that six out of seven family-C V2Rs (Vmn2r2-7) largely coexpressed and that none of the anti-Vmn2r2-7 antibodies significantly stained Vmn2r1 positive neurons. Thus, basal neurons are divided into two complementary subsets. The first subset (Vmn2r1-positive) preferentially coexpresses a distinct group of family-ABD V2Rs, whereas the second subset (Vmn2r2-7-positive) coexpresses the remaining group of V2Rs. Phylogenetic reconstruction and the analysis of genetic loci in various species reveal that receptors expressed by this second neuronal subset are recent branches of the V2R tree exclusively present in mouse and rat. Conversely, V2Rs expressed in Vmn2r1 positive neurons, are phylogenetically ancient and found in most vertebrates including rodents. Noticeably, the more recent neuronal subset expresses a type of Major Histocompatibility Complex genes only found in murine species. These results indicate that the expansion of the V2R repertoire in a murine ancestor occurred with the establishment of a new population of vomeronasal neurons in which coexists the polygenic expression of a recent group of family-C V2Rs (Vmn2r2-7) and the monogenic expression of a recent group of family-ABD V2Rs. This evolutionary innovation could provide a molecular rationale for the exquisite ability in individual recognition and mate choice of murine species.

## Introduction

Success in reproduction is strongly dependent on individual recognition. In most species, pheromone detecting systems ensures that animals not only recognize individual conspecifics but can also scrutinize their social and reproductive status [1].

Rodents, in particular, possess a well developed accessory olfactory organ, the vomeronasal organ (VNO), that triggers pheromone-mediated behavioral and neuroendocrine responses, both essential features for a successful mating rate [1]. In turn, intraspecies recognition and successful reproduction in harsh environments are key factors for the worldwide spreading of some rodent species such as mouse and rat. A natural question is whether the overdeveloped system for individual recognition of these organisms is reflected in specific molecular characteristics of their chemosensory neurons.

In muridae (mouse and rat), the organization of the VNO appears rather complex in term of receptors, transduction molecules and responses to pheromone [2], [3]. Hundreds of receptors, included in three distinct G-protein coupled receptor superfamilies, have been reportedly expressed in chemosensory neurons of this accessory olfactory organ [4], [5], [6], [7], [8], [9]. Each of these receptor superfamilies is expressed in only one of two large neuronal subsets, referred to as apical and basal that project to two distinct and non-overlapping regions of the AOB, thus suggesting a dichotomic modality to elaborate the pheromonal signal [10], [11].

The apical vomeronasal sensory neurons express at least two distinct superfamilies of G-protein coupled receptors, V1Rs and TAARs, which respectively include 250 and seven members in the mouse [4], [6], [8]. V1Rs are not co-expressed with TAARs; moreover, only one intact V1R or TAAR gene is transcribed per neuron, suggesting that each apical neuron probably expresses only one receptor type (monogenic expression) [12], [13], [14]. This implies that a neuronal subset expressing a given V1R or TAAR must be considered as a distinct pheromone specific sensory unit. Indeed, physiological studies have revealed that each VNO neuron expressing a given receptor type, V1R or TAAR, is tuned to respond to a single (or perhaps very few) pheromone [6], [8], [15], [16], [17]. The basal neurons of the VNO express a superfamily of receptors, termed V2Rs, in addition to a group of nine members of the non classical class I Major Histocompatibility Complex, whose function is still being debated [5], [8], [17], [18], [19], [20], [21]. When compared to V1Rs and TAARs, V2Rs show a very different organization in the VNO neurons. V2Rs are classified in four families, namely A, B, C and D, according to sequence homology. Mouse family A includes a consistent number of receptors that have been grouped into nine subfamilies (A1-A10, with A7 only including rat genes) [22], [23], [24]. V2Rs also differs from V1Rs for their long N-terminal extracellular region, that probably includes the putative pheromone-binding region, and for the presence of six coding exons in their genes. As for TAARs and V1Rs, receptors of family A, B and D (family ABD) are expressed in a mutually exclusive manner (monogenically) with no co-expression between members of the same and of different families [25]. In the mouse, family C contains seven highly homologous (80 to 97%) receptors.

Previous observations, based on immunohistochemical studies with antibodies raised against two members of family C, namely Vmn2r1 and Vmn2r2, have shown that these receptors are expressed in two distinct neuronal subsets that overall include more than 85% of basal neurons [26]. These results led to the conclusion that family-C receptors are expressed monogenically by the same modality as that for family ABD. Moreover, they highlighted a complex receptor scheme in the basal layer of the VNO where each neuron contains at least two different V2Rs (as demonstrated, in non random combinations) [26], [27].

Physiological studies have shown that V2R expressing neurons are able to respond to both single and multiple pheromones thus raising the possibility of a larger repertoire of receptors being expressed in some neuronal subsets [28], [29], [30], [31].

Here, based on all these observations, we have investigated the possibility that some basal neurons may express a consistent population of different V2Rs. Therefore, we raised antibodies against all members of family-C receptors (Vmn2r3-7) of the mouse and investigated their expression pattern. Peculiarly, all these newly studied receptors, similarly to Vmn2r1 and Vmn2r2, are expressed in a large population of basal neurons. We performed immunohistochemical and phylogenetic studies to characterize this neuronal population and found that it represents a supplementary class of chemosensory neurons for pheromone recognition. These neurons are absent in all other mammals (including Rodentia), amphibians, and fishes, and they express specific combinations of phylogenetically recent subfamilies of pheromone receptors. This population provides an unprecedented example of multigenic expression of chemosensory receptors in the olfactory system.

## Results

### Expression pattern of family-C V2Rs

**Figure 1:**
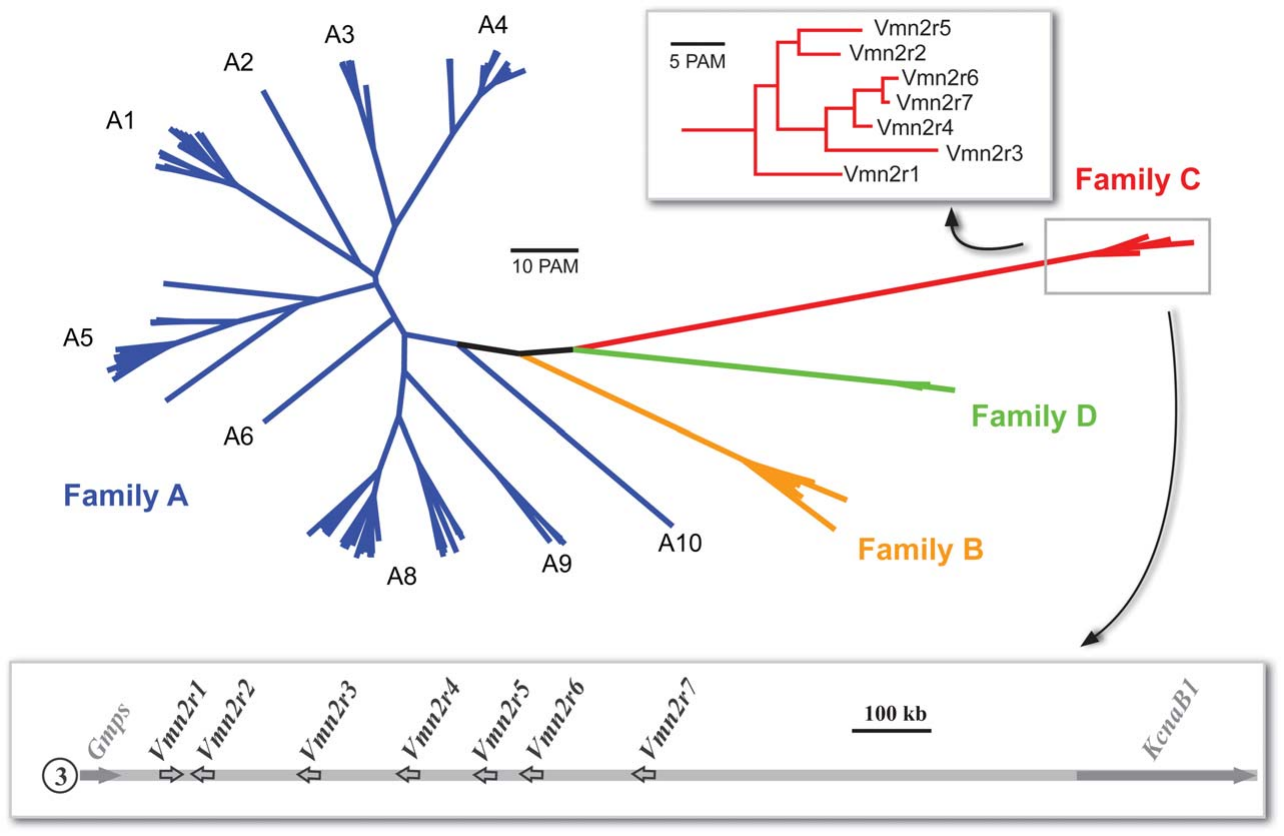
Phylogeny and genomic organization of family-C V2R genes in mouse. The unrooted phylogeny was constructed with full-length mouse V2R genes. Detail of the family-C tree is shown in the inset. The genomic organization of the family-C genes in the mouse chromosome 3 is shown below the tree.

In a previous paper, we reported that Vmn2r1 and Vmn2r2 expression defines two distinct populations of neurons in the basal layer of the mouse VNO that in total represent about 85% of all basal cells. This observation led to the conclusions that Vmn2r1 and Vmn2r2 are mutually exclusively expressed in the VNO neurons, suggesting that such a modality of expression could be extended to all family-C receptors, namely Vmn2r1-7 (Figure 1). Thus, we wondered whether the expression pattern of each family-C receptor defines distinct neuronal subsets or alternatively if co-expression of family-C receptors indeed occurs. For this purpose, we raised antibodies against Vmn2r3, Vmn2r4, and Vmn2r6 to obtain the whole panel of family-C V2R antibodies. The immunogenic and hypervariable region selected to raise specific antibodies for these receptors exactly corresponds to that employed to obtain antibodies against Vmn2r1 and Vmn2r2 [26] (Figure S1). In the structure of mGluR1a (PDB∶2E4B) [32], a receptor related to V2Rs, this region appears to lie on the external surface of the receptor in proximity to the highly enriched cystein domain that is responsible for the dimer formation [32]. Family-C receptors, however, are so highly similar that it resulted impossible to exclude cross reactivity between some of them. In fact, anti-Vmn2r6 recognizes Vmn2r7 as the two immunogenic peptides differ by three amino acids; similarly, anti-Vmn2r2 recognizes Vmn2r5 as they differ by four amino acids (Figure S2). No other cross reactivity has been detected between all other anti family-C antibodies; therefore, antibodies raised against Vmn2r1, Vmn2r3, and Vmn2r4 recognize a single receptor type. However, in all experiments, each antibody was preabsorbed with a mix of immunogenic peptides of each other family-C receptors. Moreover, we developed a new procedure for tissue processing in order to increase the immunochemical sensitivity (see Methods). Thus, we tested the distribution of family-C receptors.

**Figure 2:**
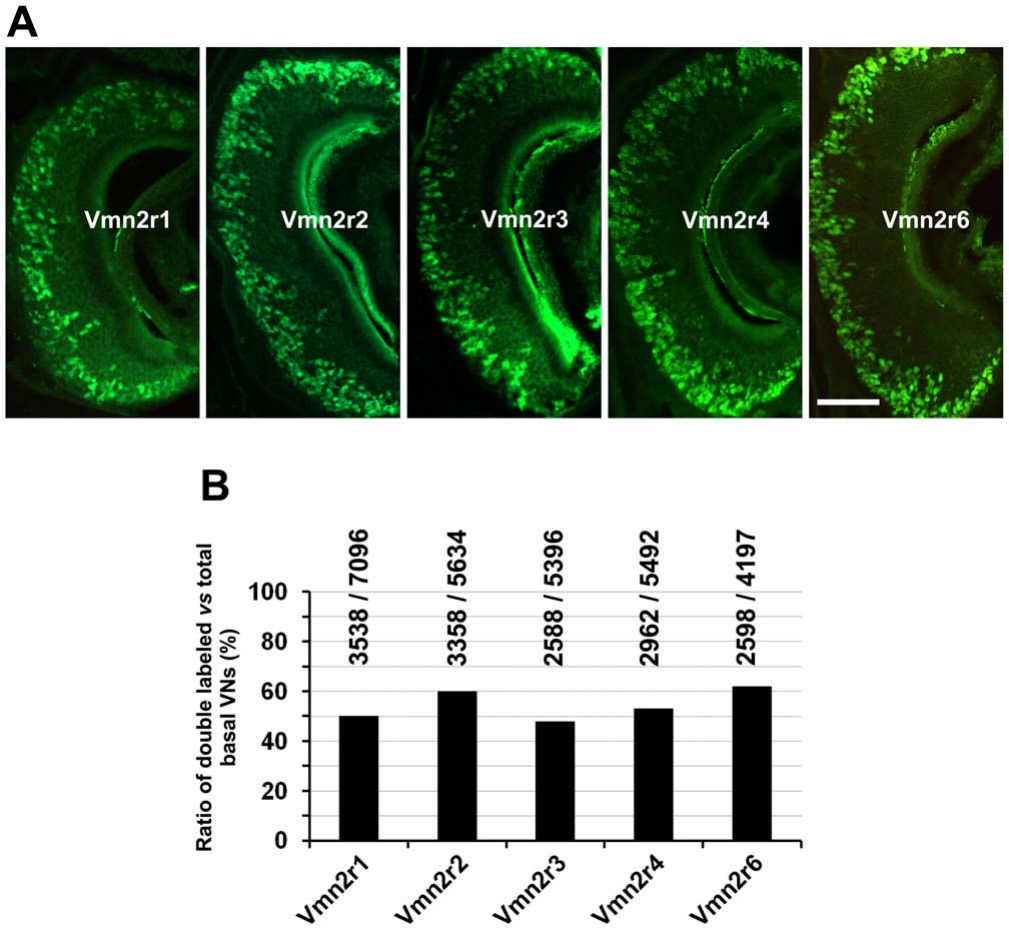
Expression pattern and distribution of family-C V2Rs in the VNO. (A) Immunolabeling of VNO sections with antibodies raised against family-C V2Rs. Scale bar = 100 µm (B) The histogram shows the percentage of basal VNO neurons (VNs) that are labelled with family-C antibodies (x-axis). The family-C receptors, Vmn2r5 and Vmn2r7, are recognized by anti-Vmn2r2 and anti-Vmn2r6 antibodies respectively. Total basal VNs were identified and counted by counterstaining sections with an antibody (panC) that recognizes all family-C V2Rs. On average, each 20 µm section from the middle-third part of the VNO contains 286±48 (s.d.) pan-C positive (basal) neurons. The number of VNO neurons analyzed is shown above each bar (double labelled/total basal neurons). For each set of experiments four 2–3-month old mice were analysed.

**Figure 3:**
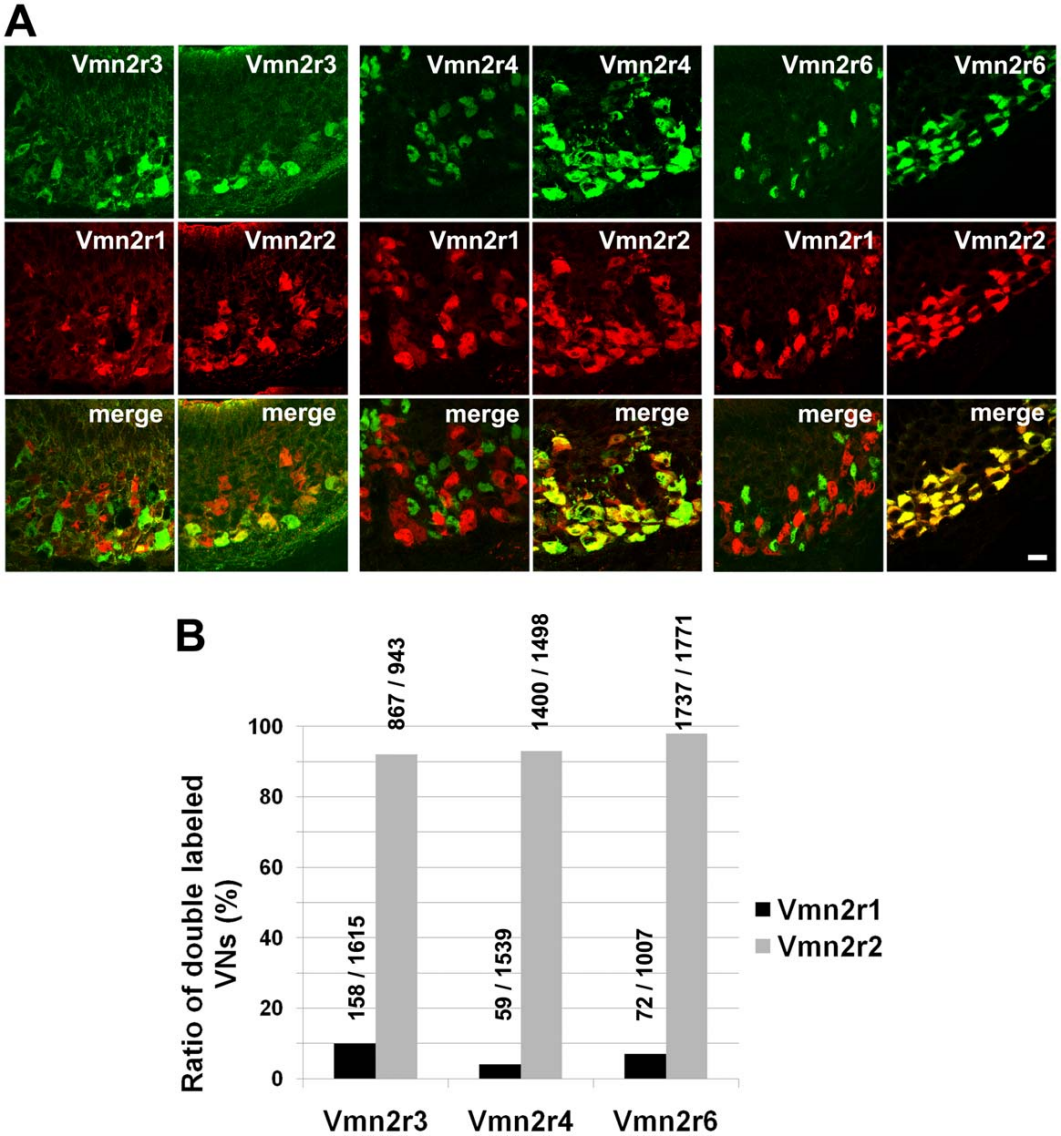
Coexpression between family-C V2Rs in the VNO neurons. (A) Double label immunohistochemistry showing the preferential coexpression of Vmn2r3, Vmn2r4, and Vmn2r6 with either Vmn2r1 or Vmn2r2 in section of the mouse VNO. Antibodies against Vmn2r3, Vmn2r4 and Vmn2r6 were used in combination with antibodies against Vmn2r1 and Vmn2r2. Scale bar = 20 µm (B) The histogram shows the percentage of Vmn2r3, Vmn2r4 and Vmn2r6 positive neurons expressing Vmn2r1 or Vmn2r2. The number of neurons is shown above each bar (double labeled/Vmn2r1 or Vmn2r2 positive neurons). Stained sections were analysed as described in the Methods section. For each set of experiments four 2–3-month old mice were employed.

Each antibody raised against a distinct family-C receptor stains a large population of VNO neurons (Figure 2). Antibodies against Vmn2r3, Vmn2r4 and Vmn2r6 stain 53%, 48% and 62% of basal cells respectively. Basal cells were identified and counted by counterstaining VNO sections with an antibody that recognized all family-C V2Rs (panC) and therefore all mature basal neurons [33]. We found that, due to the new method for tissue processing, we labeled a larger population of Vmn2r1 positive cells (50%) than reported in our previous published manuscript (26%) [26]. Thus, we observed that Vmn2r1 and Vmn2r2 stain about 96% (1056/1097, n = 3) of basal cells although a certain degree of co-expression does exist between these two receptors (5%, 74/1567, n = 3). This is consistent with a less rigorous mechanism of monogenic expression than that demonstrated for V1Rs, family-ABD V2Rs, ORs and TAARs. These results also indicate that a substantial level of coexpression must exist between some members of family-C V2Rs. The question concerns how this coexpression is being coordinated. Experiments were performed to understand the logic of coexpression between family-C V2Rs. Antibodies against Vmn2r3, Vmn2r4 and Vmn2r6 were tested in combination with both anti-Vmn2r1 and anti-Vmn2r2 in double label immunohistochemical experiments (controls for the specificity of the co-labeling experiments are reported in Figure S3). Surprisingly, results indicated that Vmn2r3, Vmn2r4 and Vmn2r6 almost exclusively co-expressed with Vmn2r2 (Figure 3). For example, respectively 92%, 93% and 98% of Vmn2r3, Vmn2r4 and Vmn2r6 positive neurons also express Vmn2r2 whereas only respectively 10%, 4% and 7% of Vmn2r3, Vmn2r4 and Vmn2r6 positive cells express Vmn2r1 (Figure 3B). Since, as aforementioned, anti-Vmn2r2 and anti-Vmn2r6 recognize Vmn2r5 and Vmn2r7 respectively (Figure S2B), it follows that Vmn2r2-7 largely coexpress in basal VNO neurons.

According to the genomic organization and the phylogenetic analysis (see in the next section) it is possible to divide family-C V2Rs into two subfamilies (hereafter termed subfamily C1 and subfamily C2) containing Vmn2r1 and Vmn2r2-7 respectively (Figure 1). This subdivision well matches with the coexpression patterns of these receptors, above described.

**Figure 4:**
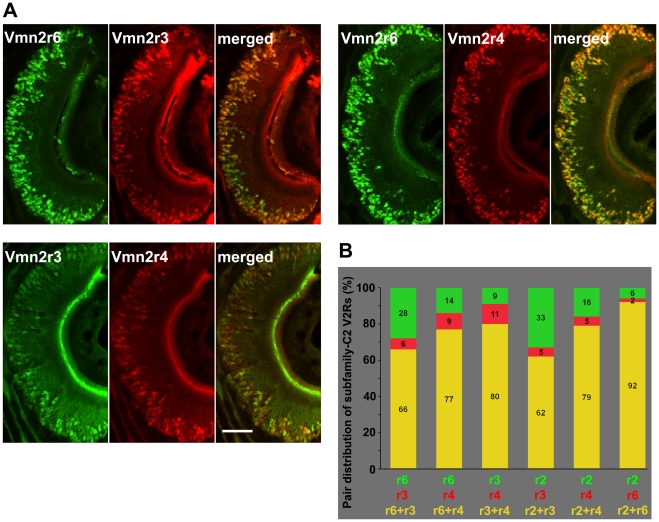
Coexpression of subfamily-C2 V2Rs. (A) Double label immunohistochemistry showing the coexpression pattern of three representative subfamily-C2 V2Rs. Scale bar = 100 µm (B) The histogram quantifies the relative coexpression of pairs of all subfamily-C2 V2Rs. Mouse VNO sections were double stained with combinations of two anti-subfamily-C2 antibodies. The relative value of each column refers to the total number of neurons (given as 100%) that resulted positive when sections were stained with both antibodies. Green and red columns refer to the ratio of VNO neurons that are positive for a single antibody. In each experiment, three groups of six serial sections throughout the VNO of the same animal were double stained with the different antibody combinations and all cells were counted. Experiments were performed on eight 2–3-month old mice.

We also attempted to investigate if the expression of different combinations of Vmn2r2-7 may identify the existence of distinct neuronal subsets. Therefore, we tested combinations of anti-Vmn2r2-7 antibodies with a double label immunohistochemical approach. Our data show that the majority of family-C2 positive neurons probably express all receptors of this subfamily (Figure 4); however, restricted neuronal subpopulations do not coexpress the entire repertoire of Vmn2r2-7 but specific combinations of them. For example, double label immunostaining with antibodies against Vmn2r3 and Vmn2r4, that recognize a single receptor type, shows that 9% and 11% of Vmn2r3 and Vmn2r4 positive cells do not express both receptors. Moreover, a relevant population of Vmn2r2 and Vmn2r6 positive cells (33% and 28%, respectively) seems to lack Vmn2r3 (Figure 4B). These observations indirectly suggest that a significant subpopulation of family-C2 positive neurons might not contain all Vmn2r2-7. Anti-Vmn2r2 and anti-Vmn2r6 antibodies (differently from anti-Vmn2r3 and anti-Vmn2r4) stain the majority of family-C2 positive cells (Figure 2B) and always coexpress in the same neurons; however, the larger distribution of Vmn2r2 and Vmn2r6 in the basal neurons might reflect the cross-reactivity of their antibodies with Vmn2r5 and Vmn2r7 respectively.

### Evolutionary origin of the V2R coexpression

**Figure 5:**
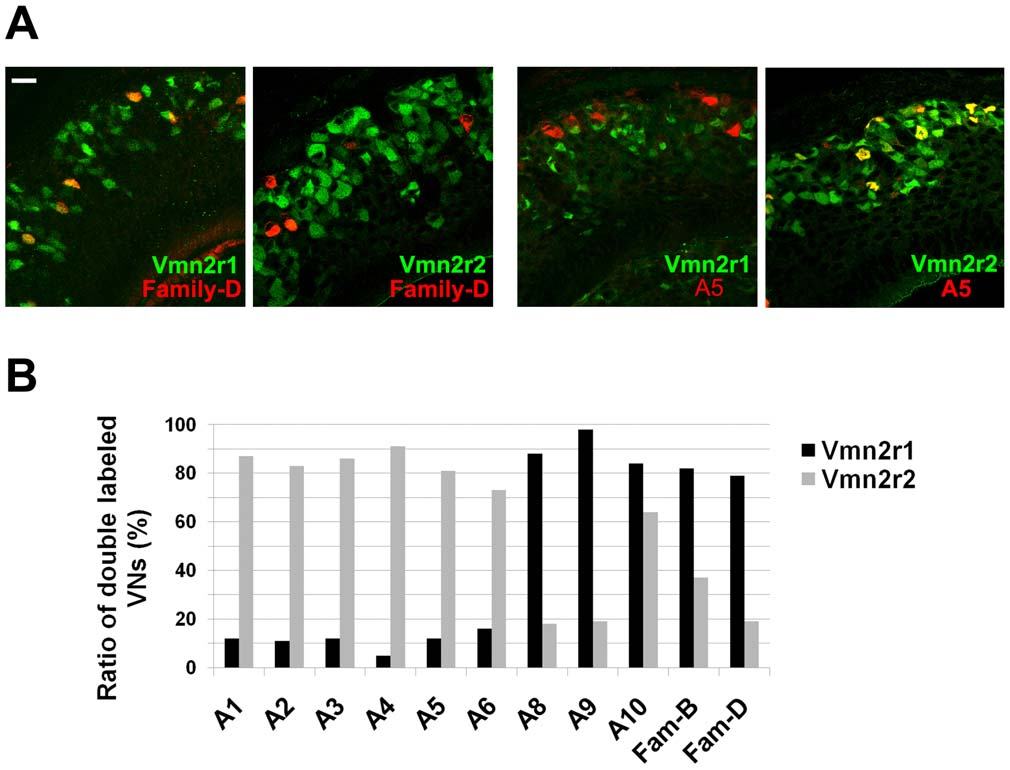
Preferential distribution of family-ABD V2Rs in subfamily-C1 and subfamily-C2 positive neurons. (A) Preferential distribution of family D and subfamily A5 V2Rs in either Vmn2r1 or Vmn2r2 positive neurons in the mouse VNO. Sections of the VNO were double labelled with antibodies against family D or family A (subfamily A5) in combination with either anti-Vmn2r1 (subfamily C1) or anti-Vmn2r2 (subfamily C2) antibodies. Scale bar = 20 µm. (B) Summary of the coexpression experiments shown in (A). The histogram shows the percentage of family-ABD immune-positive neurons (shown on x-axis) expressing Vmn2r1 or Vmn2r2. For each set of coexpression experiments four 2–3-month old mice were analyzed. Due to the very low level of expression, the analysis of the distribution of subfamily A10 was performed on eight mice. The number of VNO neurons analyzed is reported in Table S1.

In a previous work, we observed that Vmn2r1 and Vmn2r2 positive cells preferentially express specific family-ABD V2Rs. Due to the lack of antibodies against all V2R families, we tested the coexpression of Vmn2r1 and Vmn2r2 with other family-ABD V2Rs by double label in-situ hybridization (ISH), which often yields technical problems especially in the evaluation of results. Moreover, ISH cannot elude the detection of pseudogenes, which represent a consistent population of the V2R genes [22], [24]. Therefore, to complete the study, we raised specific antibodies against each V2R family (Figure 1,) and tested them with anti-Vmn2r1 (subfamily C1) and Vmn2r2 (as a representative of the subfamily C2) antibodies in experiments of double label immunohistochemistry (Figure 5, Figure S4 and Figure S5). Thus, we could proceed to the comparative analysis of all family-ABD and family-C V2Rs. Overall, we definitively established that V2Rs of subfamilies A1–A6 preferentially express in subfamily-C2 positive neurons (therefore expressing Vmn2r2-7). In contrast, receptors of subfamilies A8-10, family B and family D seem to preferentially coexpress in subfamily-C1 positive neurons (Figure 5 and Table S1). Thus, we demonstrated that coexpression between V2Rs in each basal neuron does not occur randomly but it follows a precise pattern. Moreover, a correlation seems to arise between the expression of family-C V2Rs and the phylogenetic position of the coexpressed family-ABD V2Rs.

**Figure 6:**
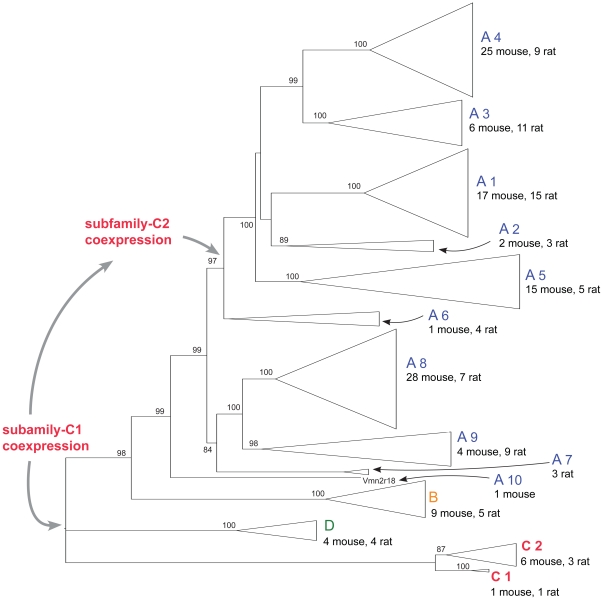
Evolution of coexpression patterns in murine V2Rs. The neighbor-joining phylogenetic tree has been rooted with the family-C receptors. Branches corresponding to previously defined families have been collapsed and are shown as triangles. Bootstrap values >75% (over 1000 replicates) are shown at the corresponding nodes. The supposed origins and evolutionary shift of V2R coexpression patterns are indicated by gray arrows.

To explore this correlation we constructed polarized phylogenies including full-length V2R sequences from *Mus musculus*, *Rattus norvegicus* (Figure 6) and full or near full-length V2R sequences extracted from the draft genomes of the closely related *Cavia porcellus* (guinea pig) and *Oryctolagus cuniculus* (rabbit) (Figure S6 and Data S1). Identical topologies were obtained by rooting the V2R trees by midpoint or by using the family C as an outgroup, consistent with a basal position of this family in the V2R phylogeny. At variance with family-ABD V2Rs, family-C orthologs are found in all species that express functional V2Rs, indicating that this family was already present in the common ancestor of bony fishes and terrestrial vertebrates [22]. Family C is typically represented by a single gene in different species, with the exception of rodents, where a modest expansion is observed [24]. As mentioned before, phylogenetic analysis and the examination of the family-C genetic locus in various organisms allow the identification of two distinct subfamilies of receptors (Figure 6 and Figure S7A). A subfamily C1, containing genes that are orthologous in various vertebrates, and a subfamily C2 containing paralogous genes originated by duplication and inversion of an ancestral C1 gene (Figure S7B). This event occurred before the separation of mouse and rat (muridae), but, within rodents, after the last common ancestor of muridae and cavioidea [34], as implied by the absence of C2 genes (and pseudogenes) in rabbit and guinea pig (Figure S7).

The co-presence of mouse and rat sequences in the majority of the groups in which family-ABD receptors are subdivided, indicates that these subdivisions have already been established in a murine ancestor, although the various groups have been subjected to species-specific expansions (Figure 6). Family-B and family-D and the most basal branches of family-A genes (subfamilies A7–A10) also contain members from rabbit or guinea pig (Figure S6). By contrast, a well-supported monophyletic group within family-A (subfamilies A1-A6) contains only mouse and rat members. Noticeably, this group corresponds to receptors that are preferentially coexpressed with the family-C2 V2Rs (Figure 5).

Based on this phylogenetic reconstruction, a simple scheme can be proposed to explain the origin and evolution of the V2R expression pattern observed in mouse (Figure 6). Coexpression between family-C1 V2Rs and basal members of family-ABD V2Rs could have been established early in the V2R evolution, given that a C1-ABD subdivision was already present in a bony fish ancestor [22]. Family-ABD receptors have been subjected to considerable expansion in vertebrates while probably maintaining coexpression with family-C1 V2Rs (through mutual exclusion within family-ABD receptors). A shift in the coexpression pattern began in a murine ancestor with the branching of the C2 and A1-A6 subfamilies and the establishing of a novel coexpression pattern in vomeronasal receptors. This pattern has been maintained with the expansion of the two subfamilies in mouse.

## Discussion

We were interested in understanding the logic of expression of V2Rs in the vomeronasal organ of rodents. We have recently shown that two receptors, namely Vmn2r1 and Vmn2r2, are expressed in distinct populations of the basal layer of the mouse VNO. Moreover, V2Rs that belong to families different from family C preferentially coexpress with Vmn2r1 or Vmn2r2 [26]. The mutually exclusive expression of Vmn2r1 and Vmn2r2 (only overlapping in a restricted population of neurons) suggested that mechanisms governing family-C receptor expression might be similar, although not so tightly regulated, to that controlling the monogenic expression of odorant receptors, V1Rs, family-ABD V2Rs and TAARs [12], [14], [25], [35]. Since family-C V2Rs account for seven receptors, the next question was whether each of these receptors defines a specific subpopulation of neurons or if, alternatively, family-C V2Rs coexpress in the same neuron. For this purpose, we raised antibodies against three new family-C V2Rs, namely Vmn2r3, Vmn2r4 and Vmn2r6 and tested their expression pattern. We found that each of these antibodies stains a large population of basal neurons, suggesting that coexpression of one or more receptors of family C occurs. Our data show that all these newly studied receptors preferentially coexpress with Vmn2r2 and only minimally with Vmn2r1 (see Figure 3); therefore family-C receptors specify two neuronal subpopulations namely, Vmn2r1 positive and Vmn2r1negative. According to our phylogenetic analysis, Vmn2r1 and Vmn2r2 are placed into two distinct subfamilies within the family-C tree; subfamily C1 includes mouse Vmn2r1 and rat Vom44 as well as orthologous genes from other species. In contrast, subfamily C2, that includes the remaining mouse (6 genes) and rat (3 genes) V2Rs, does not have a counterpart in other organisms (see Figure S7A). Thus, it appears that the coexpression pattern of family-C V2Rs reflects the evolutionary diversification and expansion of these receptors in mouse and rat. This hypothesis is strongly supported by the analysis of the genetic loci of family-C genes in different species suggesting an event of gene duplication and inversion that occurred in a common ancestor of mouse and rat (see Figure S7B).

On which bases does a VNO basal neuron selectively express either a single receptor of subfamily C1 or multiple receptors of subfamily C2? Recent data suggest that family-C V2R expression is tightly dependent on the expression of family-ABD receptors, in that these genes are likely to direct the alternative expression of Vmn2r1 or Vmn2r2 [27]. Here, we find that Vmn2r2-7 (subfamily C2) coexpresses with a distinct group of family-ABD genes (subfamilies A1–A6) while Vmn2r1 (subfamily C1) coexpresses with the remaining group of family-ABD genes. A similar pattern of expression was observed in rat (Figure S8).

There is no apparent correlation between chromosomal location or gene orientation of family-ABD receptors and their preferential coexpression with a given subfamily C (Table S2). In contrast, we found a clear correspondence between the coexpression patterns and the phylogenetic position of the different receptors in the V2R tree. In particular, receptors that have been identified as the most recent within family C, namely subfamily-C2 V2Rs, are coexpressed with a similarly recent group of receptors within family ABD, namely subfamily A1–A6 V2Rs. Both groups of receptors are found in mouse and rat (muridae) but not in any of the considered vertebrate species including the closely related guinea pig (Rodentia) and rabbit (Lagomorpha). This conclusion is also supported by a search in other rodent species with low genome coverage such as kangaroo rat and squirrel (data not shown). Since the identity of a VNO neuron is defined by the pheromone receptors that it expresses, the existence of neurons expressing a combination of two phylogenetically recent classes of V2Rs suggests that muridae developed a novel population of chemosensory cells.

It has been previously reported that a subpopulation of neurons exclusively expresses a specific type of the non-classical class Ib proteins of the Major Histocompatibility Complex (MHC) in the mouse VNO [18], [20]. These atypical MHC proteins are clustered in the M10 locus that includes nine functional genes in the mouse and seven in the rat. In contrast to classical MHC molecules, proteins of the M10 locus do not appear to bind any form of MHC peptides, consistently with the absence of a suitable MHC peptide-binding groove [21]. The function of M10 proteins in the VNO is still unclear [19], [20]; however, M10 genes have been shown to express in Vmn2r1 negative cells [19], [36], therefore in a neuronal population that overlaps with the phylogenetically recent subset here defined. Noteworthy, we were not able to identify these atypical MHC molecules outside of mouse and rat by homology searches in the available draft genomes of rodent species and rabbit (not shown). Thus, these observations, if confirmed, might suggest that the diversification of MHC M10 molecules occurred in muridae in coincidence with the establishment of a supplementary class of chemosensory neurons expressing multiple combinations of novel V2R subfamilies.

Do the newly generated class of neurons, in which a multigenic receptor expression occurs, respond to several pheromones? Different peptides are apparently capable of activating the same neurons, suggesting that multiple receptors are involved in the signal transduction of these molecules [30], [37]. The multigenic expression observed in the Vmn2r1 negative neurons raises the possibility that responses to mix of pheromones might be triggered by multiple combinations of V2R receptors within the same neuron.

Evidence from immunohistochemistry and comparative genomics presented in this work reveals a nuanced picture of the vomeronasal organ in rodent species. Noticeably, two different neuronal populations exist in the basal VNO of mouse and rat. One population expresses phylogenetically ancient V2R families that are found in other species, including terrestrial and marine vertebrates. The other population expresses multiple combinations of V2R subfamilies and MHC molecules that were more recently established in a murine ancestor. The more complex organisation of the vomeronasal organ developed in mouse and rat could provide a molecular rationale for the exquisite chemosensory ability in individual recognition and mate choice, prominent features of murine species.

## Materials and Methods

### V2R genes

Sequences of murine V2R genes were retrieved from literature or kindly provided by Dr Young. V2R nomenclature is according to Young and Trask [24].

### Tissues

For immunohistochemistry, two month old, FVB mice were deeply anaesthetized with pentobarbital and transcardially perfused at room temperature with a solution containing 10% saturated picric acid, 2% paraformaldehyde in PBS for 5′ followed by a 50 ml of PBS. Vomeronasal organs were then dissected, decalcified in EDTA 0.5 M pH 8.0 for 48–72 hrs at 4°C, and cryo-protected in 30% sucrose at 4°C overnight. Subsequently, tissues were included in OCT embedding solution (CellPath, UK) and frozen in liquid nitrogen cooled pentane.

Cryostat-cut sections (20 µm) were treated with 0.5% sodium dodecyl-sulphate for 10 min, washed in PBS, prior of the incubation with the primary antibody.

All experiments were carried out in rodents and exclusively included painless suppression of animals. The experiments comply with the Principles of Animal Care (publication no. 85–23, revised 1985) of the National Institutes of Health and with the current law of the European Union and Italy. The present project was approved by the Ethical Committee of the University of Parma: approval ID: 57/07, December 10^th^, 2007.

### Isolation and expression of V2Rs

The sequence corresponding to the C-terminal region of the extracellular domain of the mouse family-C receptors, Vmn2r3 and Vmn2r4 and Vmn2r6 was amplified from VNO cDNA with the following primers:

Vmn2r3: 5′- TGGATCCAATCAGAGTTTTAATAAAAGA -3′,
5′- ACTCGAGTTAAACATTAAGCTCTATTTTTCT -3′;
Vmn2r4: 5′-TGGATCCAATCAGAGTTTTAATAAAAGA -3′,
5′- ACTCGAGTTAAACATTAAGCTCTATTTTTCT -3′;
Vmn2r5: 5′-AATTGTGACTTATTGGAAGAA -3′,
5′- TGCCATTAAGTTCTATTTCTG -3′
Vmn2r6: 5′-TGGATCCATAGATCGTTTTAGCCAAGCT -3′,
5′- TCAAATGAATTTTATTTTTGTCAAGTACA-3′
Vmn2r7: 5′- GGAAAATAGAACTTGGCAGGA -3′,
5′- TCCTGCCAAGTTCTATTTTCC -3′

The PCR products were cloned in pGEM-T easy (Promega) and subjected to sequence analysis.

Both forward and reverse primers respectively included a BamHI and XhoI site that were used for cloning in pGEX-T (GE Life Science).

An *E.coli* based expression system (XL1B cells, Stratagene) was used to produce peptides encoding part of the extracellular domain of V2Rs. Peptide expression was induced with 1 mM isopropylthiogalactoside (Sigma) for 5 hrs at 37°C. After centrifugation, bacterial pellets were resuspended in 10 mM Tris, 150 mM NaCl, 1 mM PMSF, pH 8.0 and sonicated for 1 min. After centrifugation, pellets were washed in 10 mM Tris, 150 mM NaCl, and 1% Triton X-100, pH 8.0 and sonicated. This step was repeated three times and the final pellet, highly enriched in the peptide of interest, was dissolved in sample buffer and subjected to preparative SDS-PAGE. Purification of the fusion protein was performed by electroelution. After extensive dialysis, the peptides were lyophilized and resuspended in sterile saline solution at a concentration of 1 mg/ml.

The extracellular regions of Family-A and family-D receptors were amplified from VNO cDNA with the following primers:

Vmn2r119 (family A, subfamily A2): 5′- ACTATTTCACAATATCATGGAGAT -3′,
5′- GCCTCAGGATATAGCCAGTGACTCGAG -3′.
Vmn2r70 (family A, subfamily A5): 5′- TTACAGTAGTGAATTTTCCTTTGC -3′,
5′- ACTCGAGTTAGATGTCCTTTTCTTCCTG -3′.
Vmn2r120 (family A, subfamily A6): 5′- TTACAGTAGTGAATTTTCCTTTGC -3′,
5′- ACTCGAGTTATGAAGCCTTGCCCTCCTG -3′
Vmn2r99 (family A, subfamily A8): 5′-ggatccTATCTTCCTAAGCTGTGG -3′,
5′- CTCGAGTCATGAAAATATTTCTGGCCA -3′.
Vmn2r81 (family A, subfamily A9): 5′- GTACCCAGAAGATATTTTTCTTCA -3′,
5′- ACTCGAGTTAGTGAGGAATCTCTGTAAA -3′.
Vmn2r18 (family A, subfamily A10): 5′- CTATTTTCCAAGAAGAGAAATATC -3′,
5′- CTCGAGTCAAGTCTGATTAAAATTGTT -3′.
Vmn2r56 (family D): 5′- ACATTTGGCCTTCTGTATCAC -3′,
5′- TCGAGCCACACCAGAGACCAT -3′.

The PCR products were cloned in pGEM-T Easy and subjected to sequence analysis. The fragments were then cloned in pET-28 (Novagen) and expressed in E coli (BL21) as previously described. Bacterial pellets were processed as before except that the final pellet was dissolved in 8 M urea in PBS. Purification of the peptide was performed by affinity chromatography onto a TALON metal affinity resin (Clontech, CA) according to the manufacturer's instruction. Approximately 2 to 4 mg of protein were obtained from 200 ml culture. The purity of all final products was evaluated by densitometric analysis after SDS-PAGE and resulted to be approximately 96–98%. Antisera were obtained from rabbit by InCura Srl, Cremona, Italy.

### Antibody preparation and labeling

Antibody purification was performed by ammonium sulfate precipitation followed by DEAE exclusion chromatography. The immunoglobulin fraction was extensively dialyzed against PBS and stored at a concentration of 2–4 mg/ml.

Two milligrams of the purified immunoglobulin fraction were incubated with 50 molar excess of biotinyl-amino caproic acid N-hydroxysuccinimide (Pierce) in 1 ml reaction in PBS. After two hours at room temperature, the antibody was separated from the unreacted products by dialysis.

### Immunohistochemistry

Sections were blocked in 1% albumin, 0.3% Triton X-100 for 30 min and incubated with the anti-V2R antibodies in the same blocking solution for 36 hrs at 4°C. For double label immunohistochemistry, sections were first incubated with the unlabelled anti-V2R antibody and revealed with an anti-rabbit IgG conjugated with Alexa586 (or Alexa488)(1∶350)(Molecular Probes). Sections were then incubated with the biotinylated antibody and visualized with streptavidin-Alexa488 (or streptavidin-Alexa568) (1∶350). For preabsoption controls, five µg of each anti-V2R antibody were incubated with 10 µg of the polypeptide against which the antibody was raised.

Before use, to minimize cross-reactivity, each anti-V2R antibody was preadsorbed with a 1000 fold molar excess of each of the other V2R-immunogenic peptides. Antibodies were typically used at a 1∶400 dilution, whereas for biotin-conjugated antibodies the dilution was 1∶50.

Fluorescent images were obtained using a Zeiss fluorescent microscope and a Zeiss confocal microscope equipped with an argon-krypton laser.

### Cell count

Coronal sections (20 µm) were cut throughout the vomeronasal epithelium.

Labeled cells were counted on high-resolution images of sections of the central portion of the VNO taken at 25× magnification with a fluorescent microscope (Zeiss).

For double label experiments, counts of co-localizing cells were blind to the experimenter as positive cells in the two images of the same VNO section taken at different wavelength (λ_εξχ_ = 488 and 514) were independently marked with filled circles (green and red) on separate transparent layers using Photoshop. These two layers were then merged and co-localizing (yellow) and non co-localizing (red and green) circles were counted contextually.

### Bioinformatics

The search for genes encoding family-C V2Rs was conducted using vertebrate genomic sequences available in the in the Ensembl database (http://www.ensembl.org/). An initial tblastn search with the mouse Vmn2r1 protein sequence was performed to identify and retrieve the genomic contigs containing family-C V2R genes in the various species. Next, complete coding sequences and pseudogenes were determined using Genewise [38] through homology comparisons with a Hidden Markov Model (HMM) of V2Rs proteins. The V2R HMM was constructed using the Hammer package [39] with an alignment of ten manually curated full-length V2R sequences from mouse and rat. The full-length protein sequences extracted with this procedure from the genomes of mouse, rat, guinea pig, and rabbit are reported in the supporting information. Sequence alignments were carried out with Clustalw [40]. Phylogenetic analysis was performed with the neighbor-joining algorithm and the Kimura model of amino acid substitutions implemented in Clustalw. Trees were visualized and annotated with the FigTree program (http://tree.bio.ed.ac.uk/software/figtree/).

## Supporting Information

Figure S1 **Sequence alignment of all family-C mouse V2Rs.** The short hypervariable region that was considered for the production of antibodies is typed with red characters. The green bar highlights the transmembrane region of the receptors.
(TIF)

Figure S2 **Control of specificity of antibodies against family-C V2Rs.** (A) VNO sections were stained with antibodies against Vmn2r3, Vmn2r4 and Vmn2r6 which were preincubated with a mixture (10 µgr) of each other family-C immunogenic peptide (except for Vmn2r5 and Vmn2r7); anti-Vmn2r3, Vmn2r4 and Vmn2r6 were preincubated along with the peptide (IP) to which each antibody was raised. (B) VNO sections were incubated with antibodies anti-Vmn2r2 and anti-Vmn2r6 previously preabsorbed with the immunogenic peptides Vmn2r5 (r5 IP) and Vmn2r7 (r7 IP), respectively. Scale bar = 100 µm.
(TIF)

Figure S3 **Controls for co-labeling experiment with anti-family-C antibodies.** (A) VNO sections were incubated with biotinylated antibodies against Vmn2r4 and Vmn2r6 (previously preabsorbed with a mixture of each other family-C immunogenic peptide) and in turn revealed with streptavidin (SA) and an anti-rabbit secondary antibody (α-rb); (B) VNO sections were incubated with a mixture of antibodies against Vmn2r3, Vmn2r4 and Vmn2r6 and, in turn, revealed with an anti-rabbit secondary antibody (α-rb) and streptavidin (SA). Scale bar = 100 µm.
(TIF)

Figure S4 **Control of specificity of anti-family-A and anti-family-D antibodies.** (A) VNO sections were incubated with antibodies raised against subfamilies-A (A2, A5, A6, A8, A9, A10) and family-D V2Rs (previously preabsorbed with a mixture of each other V2R immunogenic peptide) and along with the peptide to which each antibody was raised (IP); Scale bar = 100 µm.
(TIF)

Figure S5 **Control of specificity of biotinylated antibodies against family-AD V2Rs.** VNO sections were incubated with biotinylated antibodies against A2, A5, A6, A8, A10 and family-D V2Rs (previously preadsorbed with a mixture of each other V2R immunogenic peptide) and in turn revealed with streptavidin (SA) and an anti-rabbit secondary antibody (α-rb); Scale bar = 100 µm.
(TIF)

Figure S6 **Phylogeny of V2Rs in rodents and rabbit.** Midpoint-rooted phylogeny of V2R showing the presence and expansion of the various families in rodents (mouse, rat, guinea pig) and lagomorpha (rabbit). Node labels are not shown. Family classification is as in Figure 1A.
(TIF)

Figure S7 **The V2R family-C genes in rodents and rabbit.** (A) Rooted phylogeny of family-C receptors showing the amplification of the subfamily-C2 in mouse and rat. Bootstrap value (over 100 replicates) are shown at the corresponding nodes. The Opossum family-C receptor has been used as an outgroup (not shown). (B) Comparison of the genomic organization of the family-C genes.
(TIF)

Figure S8 **V2Rs expression in the rat VNO.** (A) Anti-Vmn2r1 and anti-Vmn2r2 were used to stain ratVom44 (subfamily C1) and ratVom45 (subfamily C2) respectively in a double label immunohistochemistry experiment. Scale bar = 100 µm. (B) Preferential distribution of rat family D and subfamily A3 V2Rs in either ratVom44 or ratVom45 positive neurons. Sections of the rat VNO were double labelled with antibodies against family D or family A (subfamily A3) in combination with either anti-Vmn2r1 or anti-Vmn2r2 antibodies. Scale bar = 20 µm.
(TIF)

Data S1 **V2R sequences in guinea pig and rabbit.**
(PDF)

Table S1 **Distribution of family-ABD V2Rs in family-C positive neurons.**
(DOC)

Table S2 **Chromosomal organization of family-ABD genes.**
(DOC)
