# Supplementary figures and images for: A Recent Class of Chemosensory Neurons Developed in Mouse and Rat
Source: PLoS One. 2011 Sep 9;6(9):e24462. doi: 10.1371/journal.pone.0024462 (PMC3170373; doi:10.1371/journal.pone.0024462)

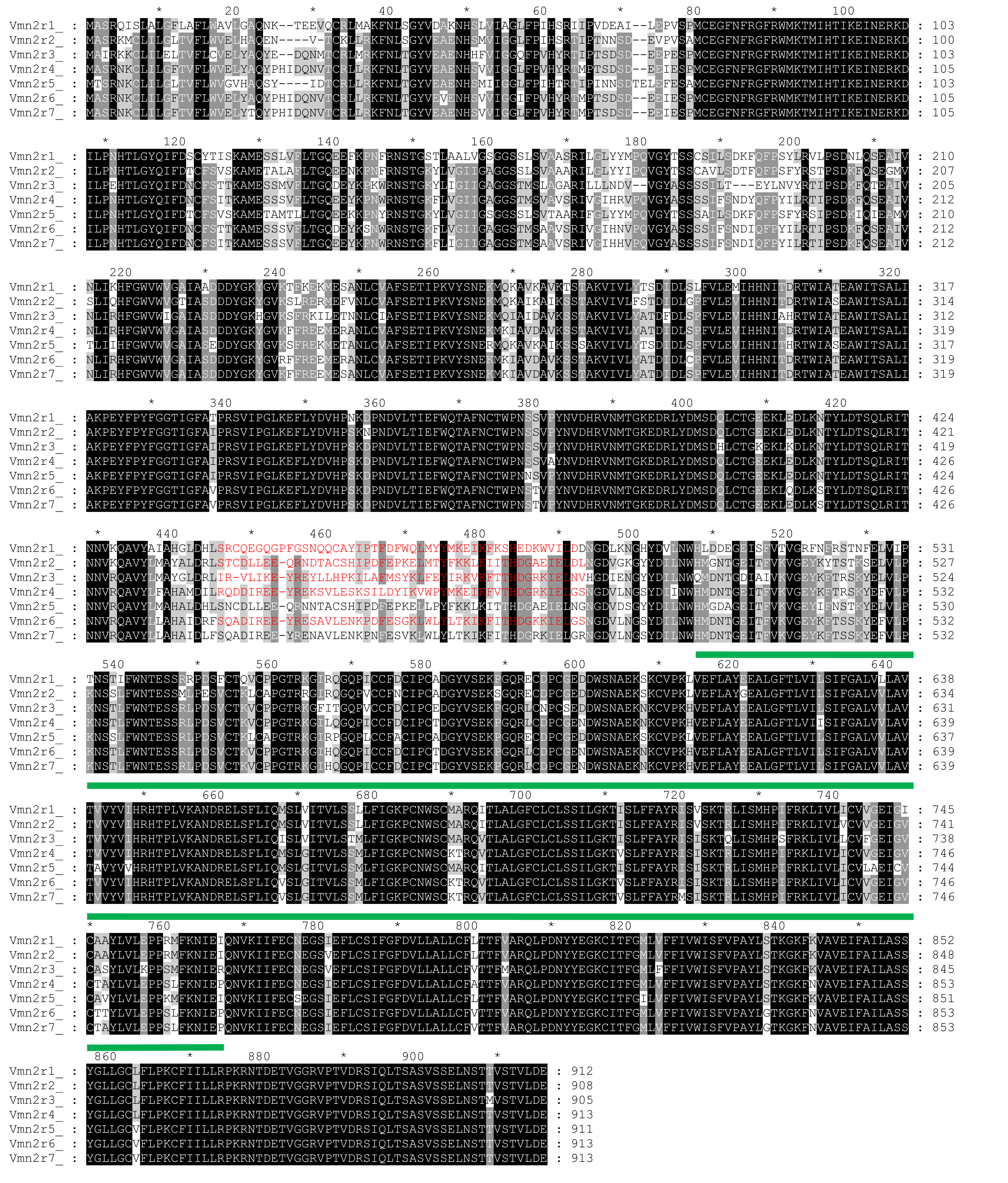

Supplement: Figure S1 — Sequence alignment of all family-C mouse V2Rs. The short hypervariable region that was considered for the production of antibodies is typed with red characters. The green bar highlights the transmembrane region of the receptors. (TIF) [file pone.0024462.s001.tif]

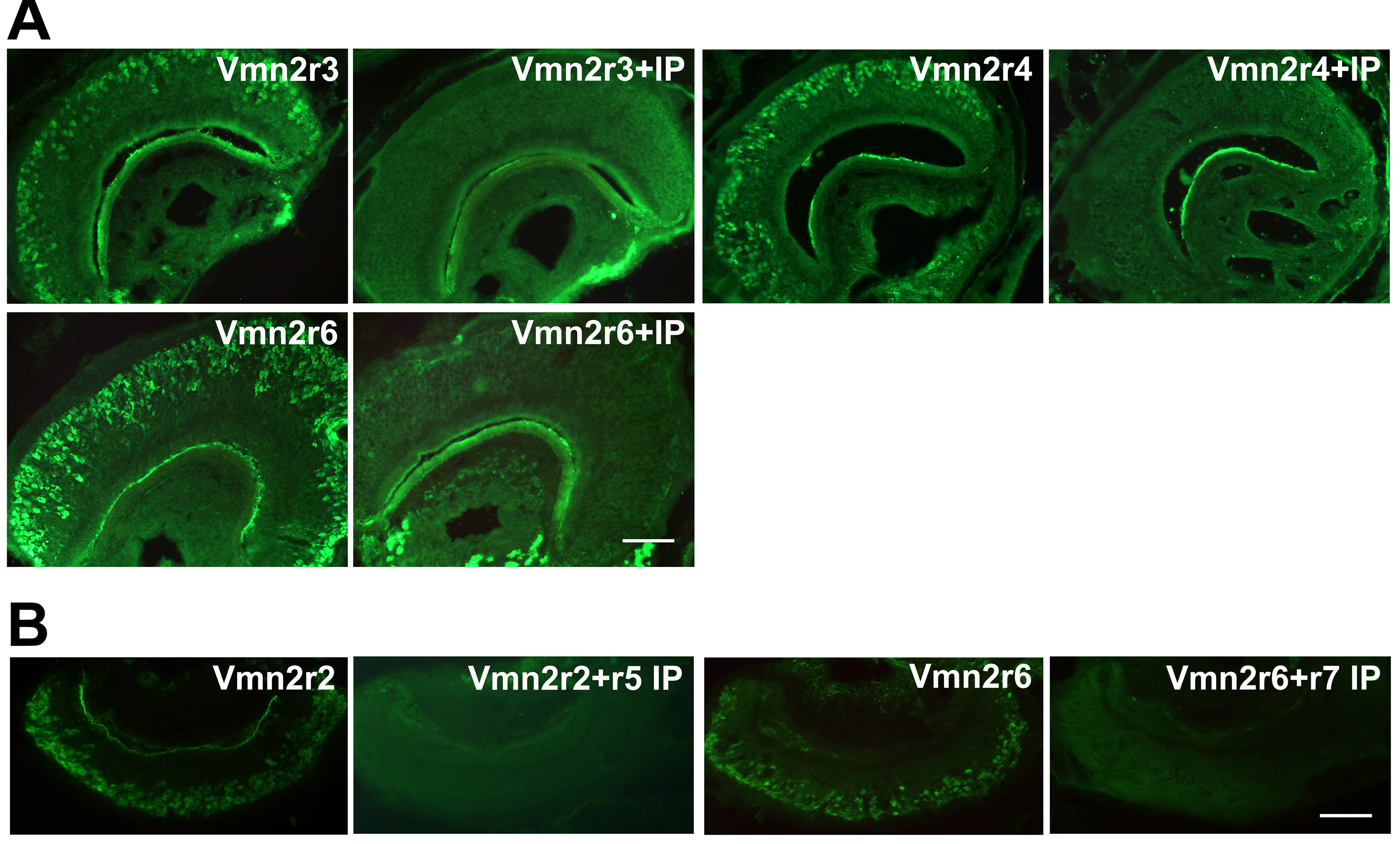

Supplement: Figure S2 — Control of specificity of antibodies against family-C V2Rs. (A) VNO sections were stained with antibodies against Vmn2r3, Vmn2r4 and Vmn2r6 which were preincubated with a mixture (10 µgr) of each other family-C immunogenic peptide (except for Vmn2r5 and Vmn2r7); anti-Vmn2r3, Vmn2r4 and Vmn2r6 were preincubated along with the peptide (IP) to which each antibody was raised. (B) VNO sections were incubated with antibodies anti-Vmn2r2 and anti-Vmn2r6 previously preabsorbed with the immunogenic peptides Vmn2r5 (r5 IP) and Vmn2r7 (r7 IP), respectively. Scale bar = 100 µm. (TIF) [file pone.0024462.s002.tif]

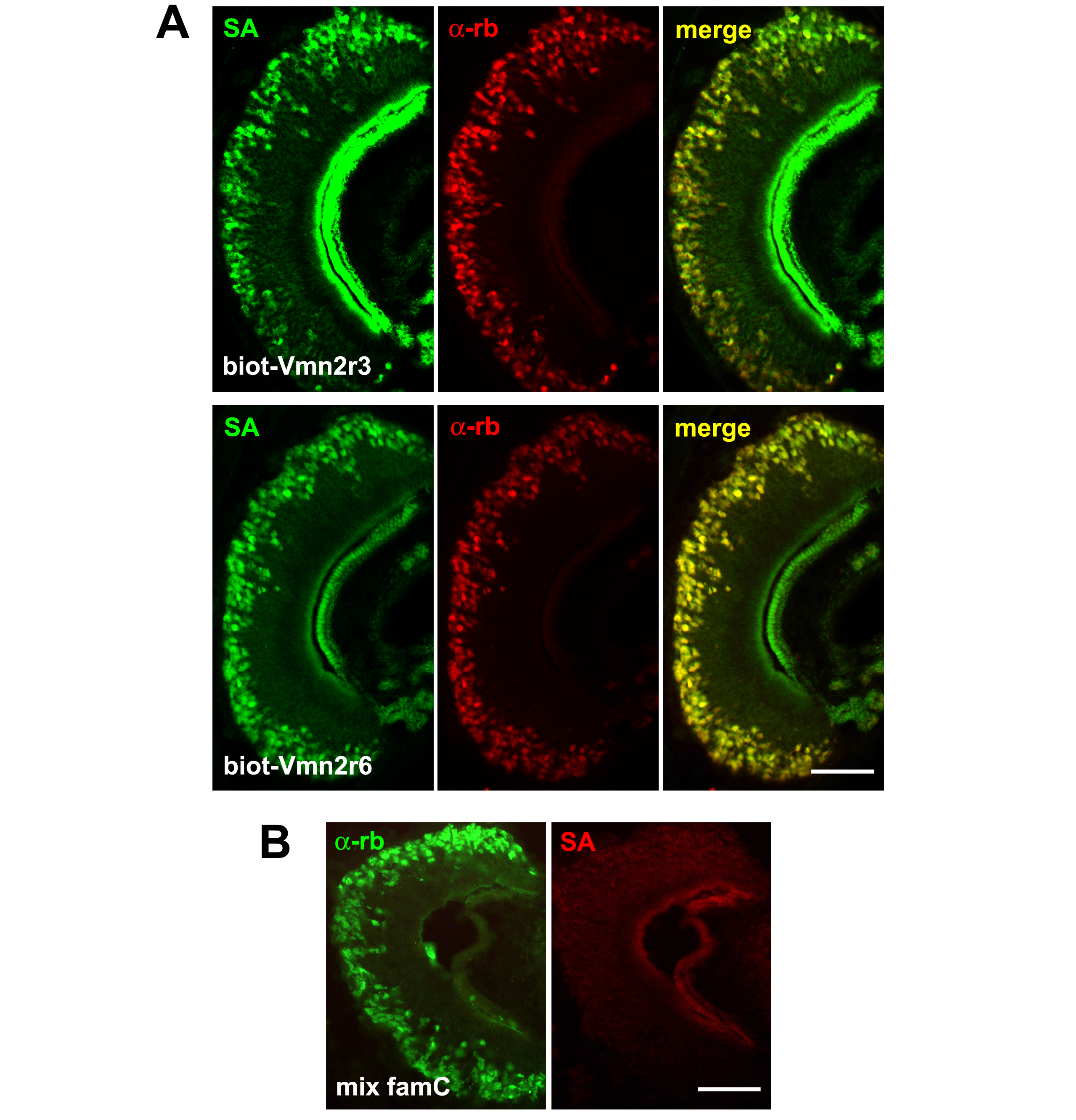

Supplement: Figure S3 — Controls for co-labeling experiment with anti-family-C antibodies. (A) VNO sections were incubated with biotinylated antibodies against Vmn2r4 and Vmn2r6 (previously preabsorbed with a mixture of each other family-C immunogenic peptide) and in turn revealed with streptavidin (SA) and an anti-rabbit secondary antibody (α-rb); (B) VNO sections were incubated with a mixture of antibodies against Vmn2r3, Vmn2r4 and Vmn2r6 and, in turn, revealed with an anti-rabbit secondary antibody (α-rb) and streptavidin (SA). Scale bar = 100 µm. (TIF) [file pone.0024462.s003.tif]

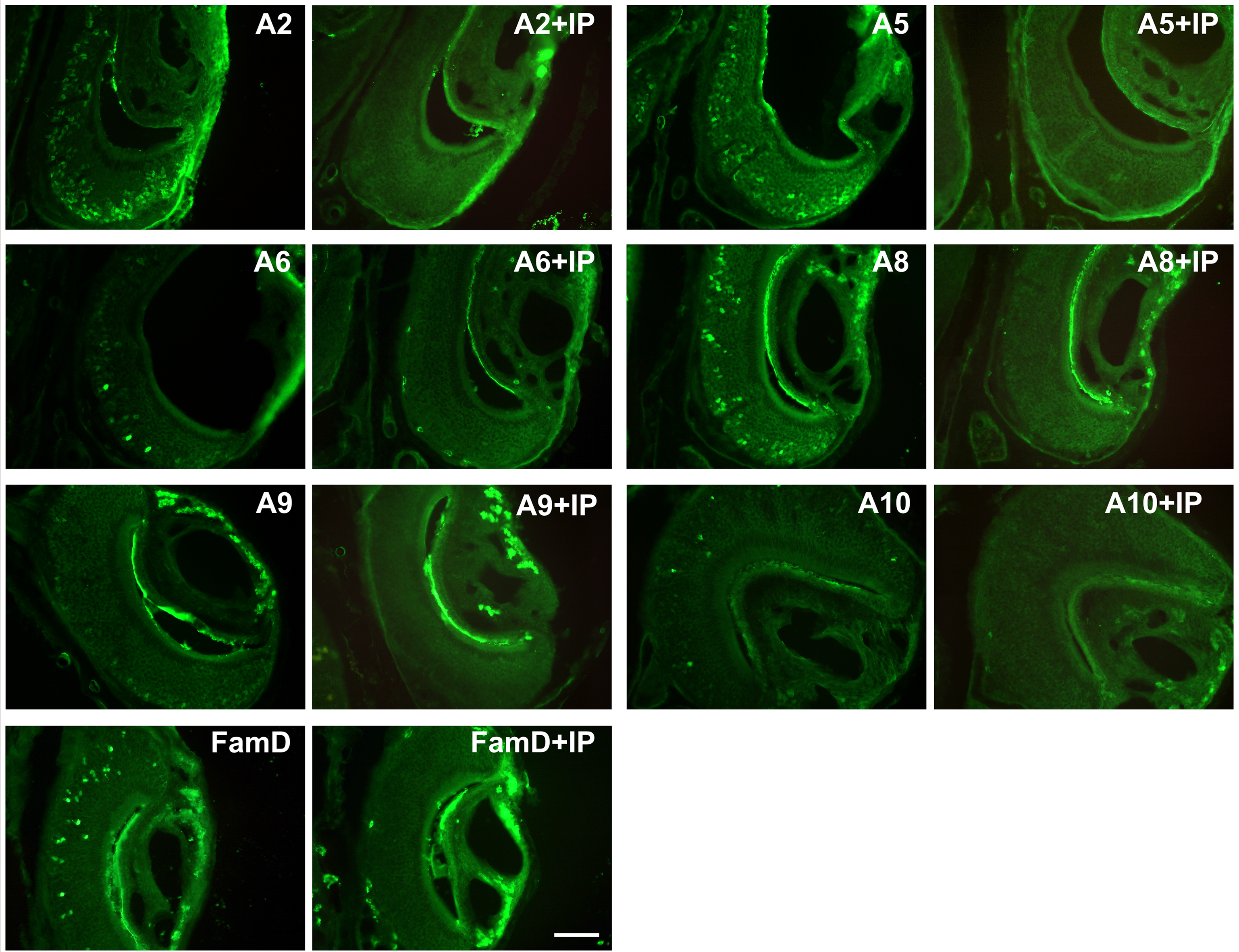

Supplement: Figure S4 — Control of specificity of anti-family-A and anti-family-D antibodies. (A) VNO sections were incubated with antibodies raised against subfamilies-A (A2, A5, A6, A8, A9, A10) and family-D V2Rs (previously preabsorbed with a mixture of each other V2R immunogenic peptide) and along with the peptide to which each antibody was raised (IP); Scale bar = 100 µm. (TIF) [file pone.0024462.s004.tif]

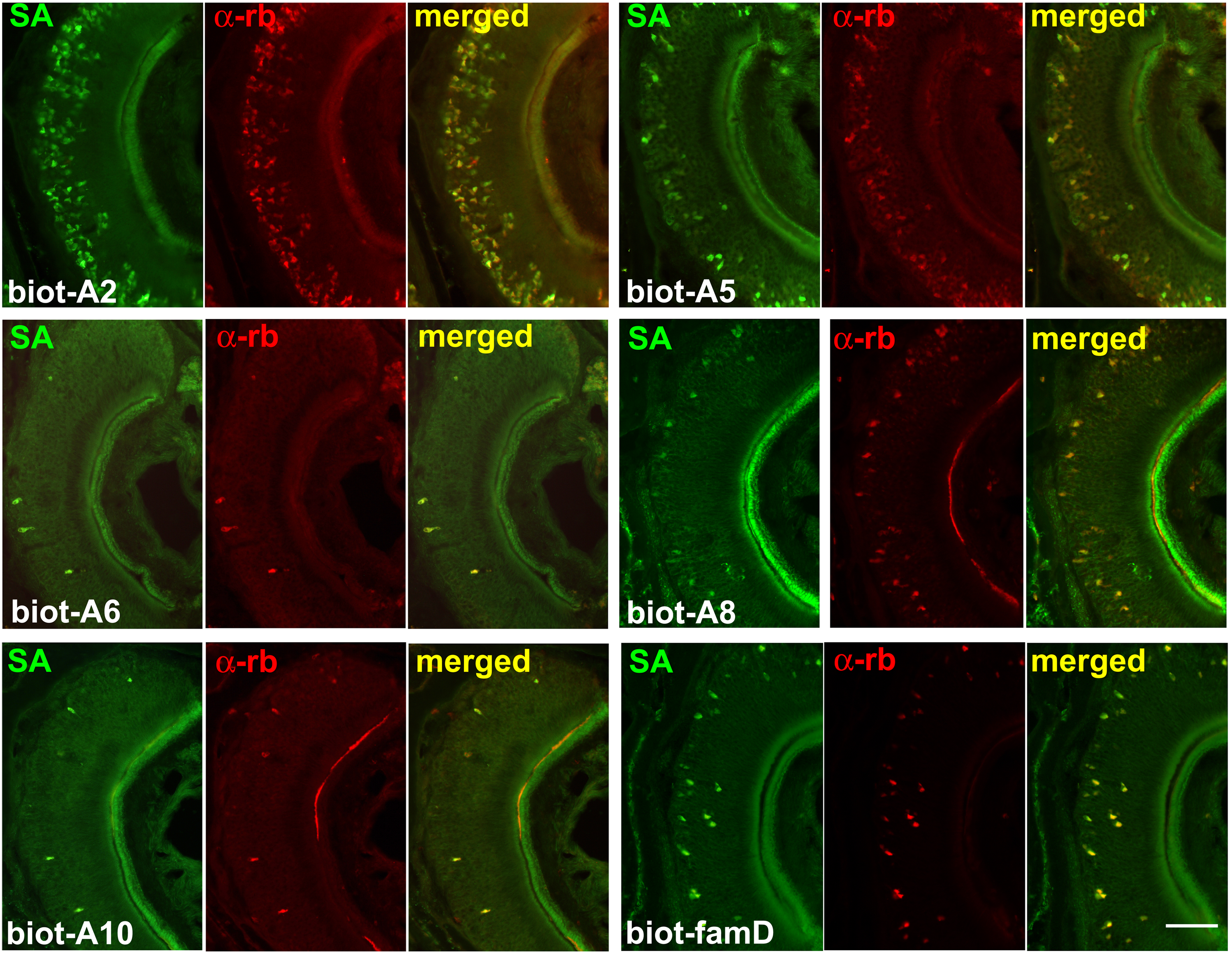

Supplement: Figure S5 — Control of specificity of biotinylated antibodies against family-AD V2Rs. VNO sections were incubated with biotinylated antibodies against A2, A5, A6, A8, A10 and family-D V2Rs (previously preadsorbed with a mixture of each other V2R immunogenic peptide) and in turn revealed with streptavidin (SA) and an anti-rabbit secondary antibody (α-rb); Scale bar = 100 µm. (TIF) [file pone.0024462.s005.tif]

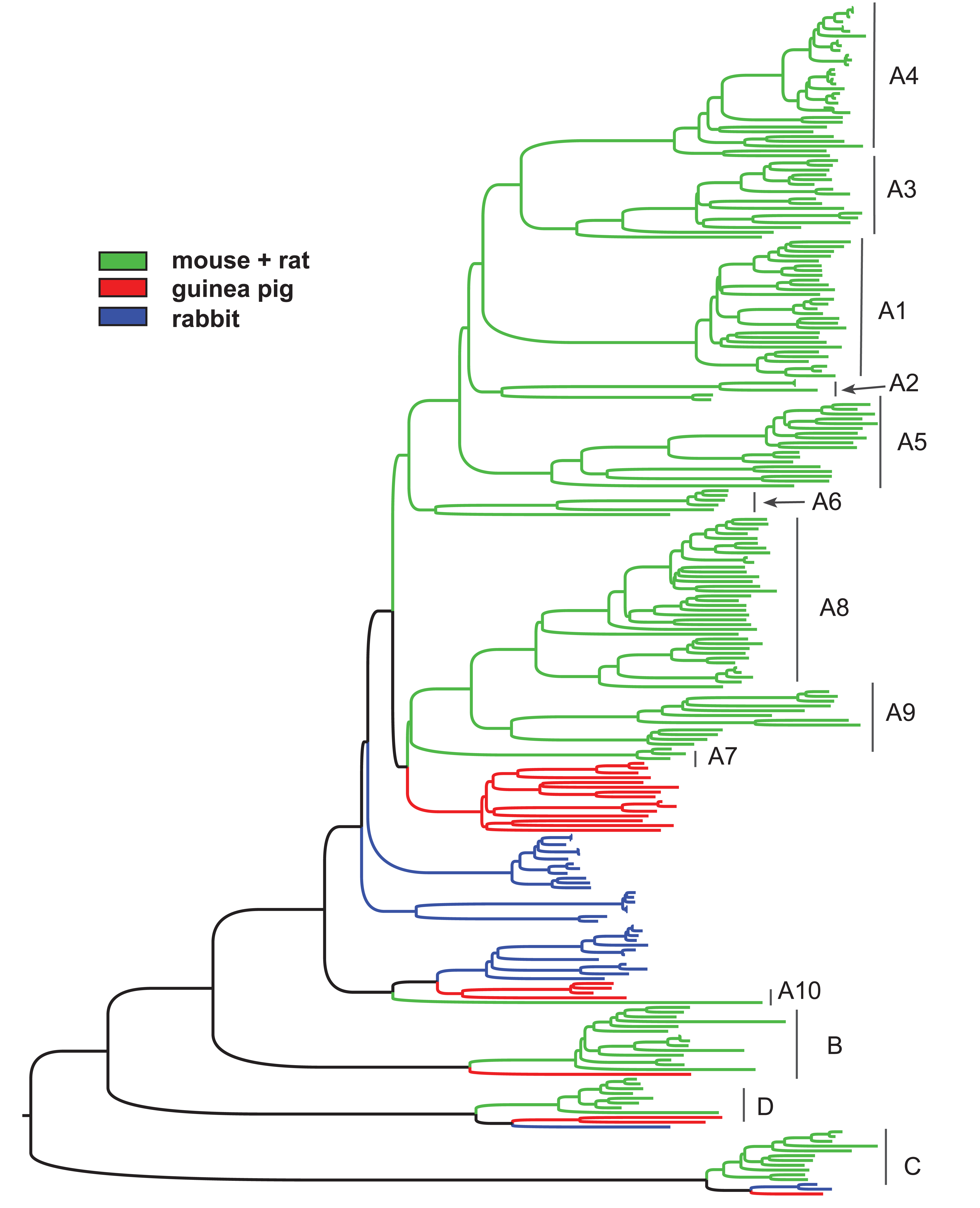

Supplement: Figure S6 — Phylogeny of V2Rs in rodents and rabbit. Midpoint-rooted phylogeny of V2R showing the presence and expansion of the various families in rodents (mouse, rat, guinea pig) and lagomorpha (rabbit). Node labels are not shown. Family classification is as in Figure 1A. (TIF) [file pone.0024462.s006.tif]

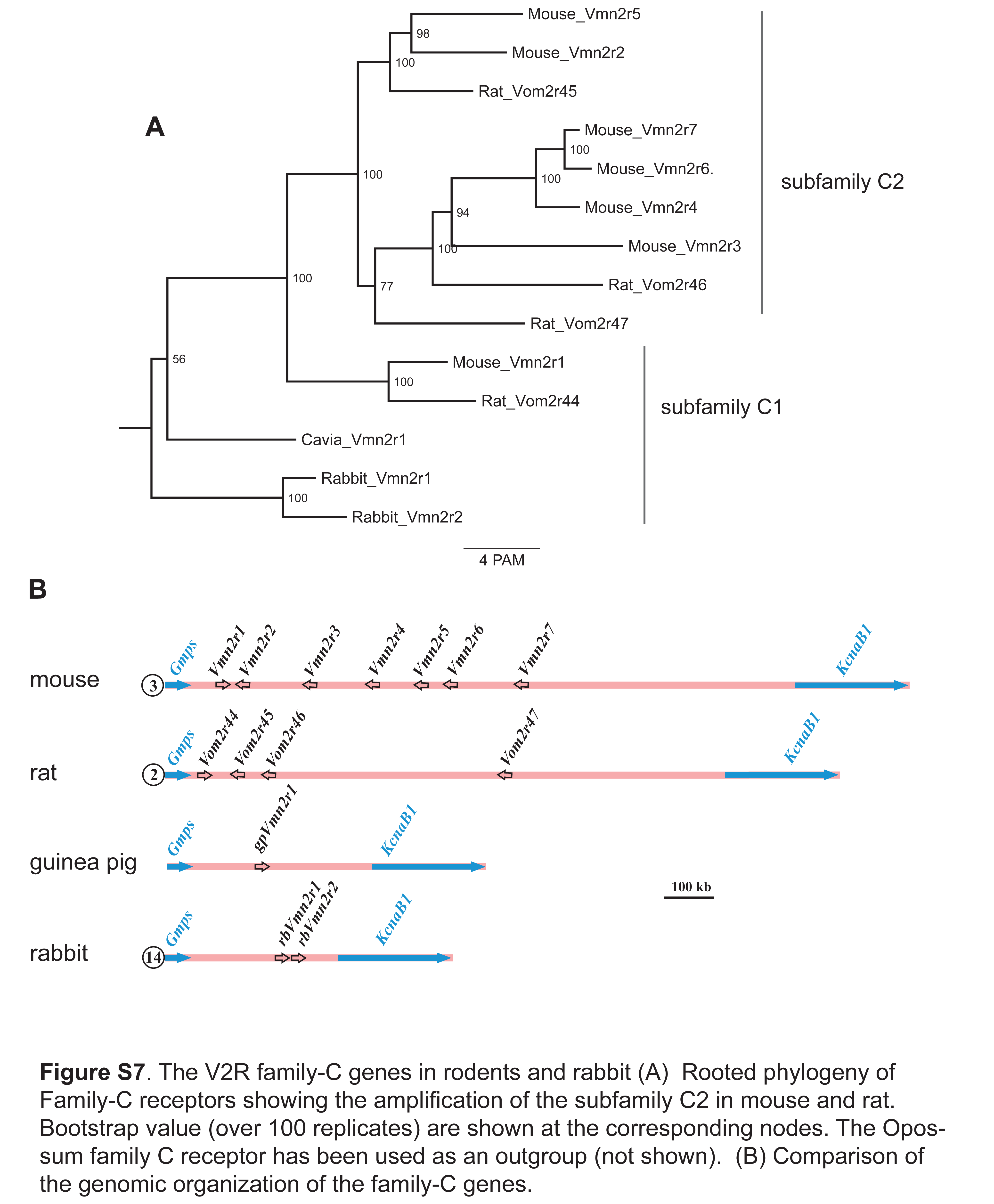

Supplement: Figure S7 — The V2R family-C genes in rodents and rabbit. (A) Rooted phylogeny of family-C receptors showing the amplification of the subfamily-C2 in mouse and rat. Bootstrap value (over 100 replicates) are shown at the corresponding nodes. The Opossum family-C receptor has been used as an outgroup (not shown). (B) Comparison of the genomic organization of the family-C genes. (TIF) [file pone.0024462.s007.tif]

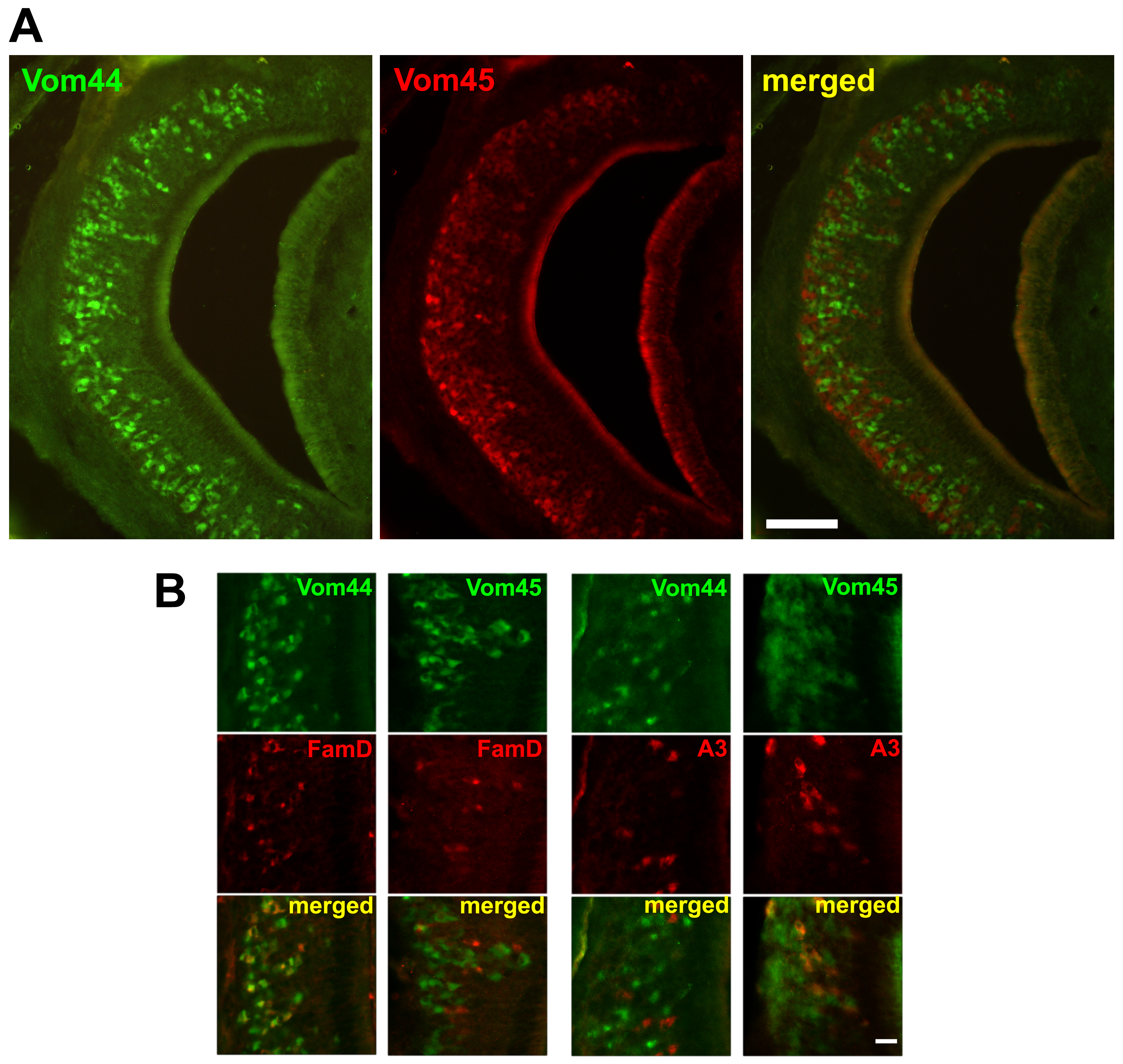

Supplement: Figure S8 — V2Rs expression in the rat VNO. (A) Anti-Vmn2r1 and anti-Vmn2r2 were used to stain ratVom44 (subfamily C1) and ratVom45 (subfamily C2) respectively in a double label immunohistochemistry experiment. Scale bar = 100 µm. (B) Preferential distribution of rat family D and subfamily A3 V2Rs in either ratVom44 or ratVom45 positive neurons. Sections of the rat VNO were double labelled with antibodies against family D or family A (subfamily A3) in combination with either anti-Vmn2r1 or anti-Vmn2r2 antibodies. Scale bar = 20 µm. (TIF) [file pone.0024462.s008.tif]
