# Supplementary material for: A Recent Class of Chemosensory Neurons Developed in Mouse and Rat
Source: PLoS One. 2011 Sep 9;6(9):e24462. doi: 10.1371/journal.pone.0024462 (PMC3170373; doi:10.1371/journal.pone.0024462)
Supplement: Data S1 — V2R sequences in guinea pig and rabbit. (PDF) [file pone.0024462.s009.pdf]

Complete sequences of Family-C V2R proteins identified in mouse (*Mus musculus*), rat (*Rattus norvegicus*) , guinea pig (*Cavia porcellus*) and rabbit (*Oryctolagus cuniculus*).

>Mouse_Vmn2r7
MASRNKCLILGFTVFLWVELYTQYPHIDQNVTCRLLRKFNLTGYVEAENHSVVIGGLFPVHYRTMPTSDSDEEIESPMCEGFNFRGFRWMKTMIHTIKEI
NERKDILPNHTLGYQIFDNCFSITKAMESSSVFLTGQEEYKPNWRNSTGKFLIGIIGAGGSTMSAAVSRIVGIHHVPQVGYASSSSIFSNDIQFPYILRTIPSD
KFQSEAIVNLIRHFGWVWVGAIASDDDYGKYGVKFFREEMESANLCVAFSETIPKVYSNEKMKIAVDAVKSSTAKVIVLYATDIDLSPFVLEVIHHNITDRT
WIATEAWITSALIAKPEYFPYFGGTIGFAVPRSVIPGLKEFLYDVHPSKDPNDVLTIEFWQTAFNCTWPNSTVPYNVDHRVNMTGKEDRLYDMSDQLCT
GEEKLEDLKSTYLDTSQLRITNNVRQAVYLLAHAIDLFSQADIREEYRENAVLENKPNFESVKLWLYLTKIKFITHDGRKIELGRNGDVLNGSYDILNWHMD
NTGEITFVKVGEYKFTSSKYEFVLPKNSTLFWNTESSRLPDSVCTKVCPPGTRKGIHQGQPICCFDCIPCTDGYVSEKPGQRLCDPCGENDWSNAEKNKC
VPKHVEFLAYEEALGFTLVILSIFGALVVLAVTVVYVIHRHTPLVKANDRELSFLIQVSLGITVLSSMLFIGKPCNWSCKTRQVTLALGFCLCLSSILGKTVSLFF
AYRMSISKTRLISMHPIFRKLIVLICVVGEIGVCTAYLVLEPPSLFKNIEPQNVKIIFECNEGSIEFLCSIFGFDVLLALLCFVTTFVARQLPDNYYEGKCITFGML
VFFIVWISFVPAYLGTKGKFNVAVEIFAILASSYGLLGCVFLPKCFIILLRPKRNTDETVGGRVPTVDRSIQLTSASVSSELNSTTVSTVLDE
>Mouse_Vmn2r6.
MASRNKCLILGFTVFLWVELYAQYPHIDQNVTCRLLRKFNLTGYVEVENHSVVIGGIFPVHYRTMPTSDSDEEIESPMCEGFNFRGFRWMKTMIHTIKEI
NERKDILPNHTLGYQIFDNCFSTTKAMESSSVFLTGQDEYKSNWRNSTGKFLVGIIGAGGSTMSAAVSRIVGIHNVPQVGYASSSSIFSNDIQFPYILRTIPS
DKFQSEAIVNLIRHFGWVWVGAIASDDDYGKYGVRFFREEMERANLCVAFSETIPKVYSNEKMKIAVDAVKSSTAKVIVLYATDIDLCPFVLEVIHHNITD
RTWIATEAWITSALIAKPEYFPYFGGTIGFAVPRSVIPGLKEFLYDVHPSKDPNDVLTIEFWQTAFNCTWPNSTVPYNVDHRVNMTGKEDRLYDMSDQL
CTGEEKLQDLKSTYLDTSQLRITNNVRQAVYLLAHAIDRFSQADIREEYRESAVLENKPDFESGKLWLYLTKIKFITHDGKKIELGSNGDVLNGSYDILNWH
MDNTGEITFVKVGEYKFTSSKYEFVLPKNSTLFWNTESSRLPDSVCTKVCPPGTRKGIHQGQPICCFDCIPCTDGYVSEKPGQRLCDPCGENDWSNAEKN
KCVPKHVEFLAYEEALGFTLVILSIFGALVVLAVTVVYVIHRHTPLVKANDRELSFLIQVSLGITVLSSMLFIGKPCNWSCKTRQVTLALGFCLCLSSILGKTVS
LFFAYRISISKTRLISMHPIFRKLIVLICVVGEIGVCTTYLVLEPPSLFKNIEPQNVKIIFECNEGSIEFLCSIFGFDVLLALLCFVTTFVARQLPDNYYEGKCITFGM
LVFFIVWISFVPAYLGTKGKFNVAVEIFAILASSYGLLGCVFLPKCFIILLRPKRNTDETVGGRVPTVDRSIQLTSASVSSELNSTTVSTVLDE
>Mouse_Vmn2r5 .
MTSRNKCLILGLTVFLWVGVHAQSYIDTCRLLRKFNLTGYVEAENHSMIIGGLFPIHTRTIPINNSDTELEFESAMCEGFNFRGFRWMKTMIHTIKEINERK
DILPNHTLGYQIFDTCFSVSKAMETAMTLLTGQEEKKPNYRNSTGKYLVGIIGSGGSSLSVTAARIFGLYYMPQVGYTSSSAILSDKFQFPSFYRSIPSDKIQI
EAMVTLIIHFGWVWVGAIASEDDYGKYGVKSFREKMETANLCVAFSETIPKVYSNERMQKAVKAIKSSSAKVIVLYTSDIDLSPFVLEVIHHNITHRTWIAS
EAWITSALIAKPEYFPYFGGTIGFAIPRSVIPGLKEFLYDVHPSKDPNDVLTIEFWQTAFNCTWPNNSVPYNVDHRVNMTGKEDRLYDMSDQLCTGEEKL
EDLKNTYLDTSQLRITNNVRQAVYLMAHALDHLSNCDLLEEQRNNTACSHIPDFEPKELLPYFKKLKITTHDGAEIELNGNGDVDSGYYDILNWHMGDA
GEITFVKVGEYIFNSTKYELVLPKNSSLFWNTESSRLPDSVCTKLCAPGTRKGIRPGQPLCCFACIPCADGYVSEKPGQRECDPCGEDDWSNAEKSKCVPK
LVEFLAYEEALGFTLVILSIFGALVVLAVTAVYVVHRHTPLVKANDRELSFLIQMSLGITVLSSMLFIGKPCNWSCMARQITLALGFCLCLSSILGKTISLFFAY
RISISKTRLISMHPIFRKLIVLICVLAEICVCAVYLVLEPPKMFKNIEIQNVKIIFECSEGSIEFLCSIFGFDVLLALLCFLTTFVARQLPDNYYEGKCITFGILVFFIV
WISFVPAYLSTKGKFKVAVEIFAILASSYGLLGCVFLPKCFIILLRPKRNTDETVGGRVPTVDRSIQLTSASVSSELNSTTVSTVLDE
>Mouse_Vmn2r4 .
MASRNKCLILGFTVFLWVELYAQYPHIDQNVTCRLLRKFNLTGYVEAENHSVVIGGLFPVHYRTMPTSDSDEEIESPMCEGFNFRGFRWMKTMIHTIKEI
NERKDILPNHTLGYQIFDNCFSITKAMESSSVFLTGQEEYKPNWRNSTGKFLVGIIGAGGSTMSVAVSRIVGIHRVPQVGYASSSSIFSNDYQFPYILRTIPS
DKFQSEAIVNLIRHFGWVWVGAIASDDDYGKYGVKFFREEMERANLCVAFSETIPKVYSNEKMKIAVDAVKSSTAKVIVLYATDIDLSPFVLEVIHHNITD
RTWIATEAWITSALIAKPEYFPYFGGTIGFAIPRSVIPGLKEFLYDVHPSKDPNDVLTIEFWQTAFNCTWPNSSVAYNVDHRVNMTGKEDRLYDMSDQLC
TGEEKLEDLKNTYLDTSQLRITNNVRQAVYLFAHAMDILRQDDIREEYREKSVLESKSILDYIKVWPYMKEIKFVTHDGRKIELGSNGDVLNGSYDIINWH
MDNTGEITFVKVGEYKFTRSKYEFVLPKNSTLFWNTESSRLPDSVCTKVCPPGTRKGILQGQPICCFDCIPCTDGYVSEKPGQRLCDPCGENDWSNAEKN
KCVPKHVEFLAYEEALGFTLVIISIFGALVVLAVTVVYVIHRHTPLVKANDRELSFLIQMSLGITVLSSMLFIGKPCNWSCKTRQVTLALGFCLCLSSILGKTVS
LFFAYRISISKTRLISMHPIFRKLIVLICVVGEIGVCTAYLVLEPPSLFKNIEPQNVKIIFECNEGSIEFLCSIFGFDVLLALLCFATTFVARQLPDNYYEGKCITFG
MLVFFIVWISFVPAYLSTKGKFNVAVEIFAILASSYGLLGCLFLPKCFIILLRPKRNTDETVGGRVPTVDRSIQLTSASVSSELNSTTVSTVLDE
>Mouse_Vmn2r3 .
MAIRKKCLILELTVFLCVELYAQYEDQNMTCRLMRKFNLTGYVEAENHHFVIGGQFPVHYRTIPTSDSDEEPESPMCEGFNFRGFRWMKTMIHTIKEINE
RKDILPEHTLGYQIFDNCFSTTKAMESSMVFLTGQDEYKPKWRNSTGKYLIGIIGAGGSTMSLAGARILLLNDVVQVGYASSSILTEYLNVYRTIPSDKFQ
TEAIVNLIRHFGWVWIGAIASDDDYGKHGVKSFRKILETNNLCIAFSETIPKVYSNEKMQIAIDAVKSSTAKVIVLYATDFDLSPFVLEVIHHNIAHRTWIAT
EAWITSALIAKPEYFPYFGGTIGFAIPRSVIPGLKEFLYDVHPSKDPNDVLTIEFWQTAFNCTWPNSSVPYNVDHRVNMTGKEDRLYDMSDHLCTGKEKL
KDLKNTYLDTSQLRITNNVRQAVYLMAYGLDRLIRVLIKEYREYLLHPKILAFMSYKLFEYIRKVKFTTHDGRKIELNVHGDIENGYYDILNWQMDNTGDIA
IVKVGEYKFTRSKYELVLPKNSTLFWNTESSRLPDSVCTKVCPPGTRKGFITGQPVCCFDCIPCEDGYVSEKPGQRLCNPCSEDDWSNAEKNKCVPKHVEF
LAYEEALGFTLVILSIFGALVVLAVTVVYVIHRHTPLVKANDRELSFLIQISLVITVLSTMLFIGKPCNWSCMARQVTLALGFCLCLSSILGKTISLFFAYRISISKT
QLISMHPSFRKLIVLLCVFGEIGVCASYLVLKPPSMFKNIERQNVKIIFECNEGSVEFLCSIFGFDVLLALLCFVTTFMARQLPDNYYEGKCITFGMLFFFIVWI
SFVPAYLSTKGKFKVAVEIFAILASSYGLLGCLFLPKCFIILLRPKRNTDETVGGRVPTVDRSIQLTSASVSSELNSTMVSTVLDE

>Mouse_Vmn2r2 .
MASRKMCLILGLTVFLWVELHAQENVTCKLLRKFNLSGYVEAENHSMVIGGLFPIHSRTIPTNNSDEVPVSAMCEGFNFRGFRWMKTMIHTIKEINERK
DILPNHTLGYQIFDTCFSVSKAMETALAFLTGQEENKPNFRNSTGKYLVGIIGAGGSSLSVAAARILGLYYIPQVGYTSSCAVLSDTFQFPSFYRSTPSDKFQ
SEGMVSLIQHFGWVWVGTIASDDDYGKYGVKSLRERMEFVNLCVAFSETIPKVYSNEKMQKAIKAIKSSTAKVIVLFSTDIDLGPFVLEVIHHNITDRTWI
ASEAWITSALIAKPEYFPYFGGTIGFAIPRSVIPGLKEFLYDVHPSKNPNDVLTIEFWQTAFNCTWPNSSVPYNVDHRVNMTGKEDRLYDMSDQLCTGEE
KLEDLKNTYLDTSQLRITNNVKQAVYLMAYALDRLSTCDLLEEQRNDTACSHIPDFEPKELMTYFKKLKIITHDGAEIELDLNGDVGKGYYDILNWHMGN
TGEITFVKVGEYKYTSTKSELVLPKNSSLFWNTESSMLPESVCTKLCAPGTRRGIRQGQPVCCFNCIPCADGYVSEKSGQRECDPCGEDDWSNAEKSKCV
PKLVEFLAYGEALGFTLVILSIFGALVVLAVTVVYVIHRHTPLVKANDRELSFLIQMSLVITVLSSLLFIGKPCNWSCMARQITLALGFCLCLSSILGKTISLFFAY
RISVSKTRLISMHPIFRKLIVLVCVVGEIGVCAAYLVLEPPRMFKNIEIQNVKIIFECNEGSVEFLCSIFGFDVLLALLCFLTTFVARQLPDNYYEGKCITFGMLV
FFIVWISFVPAYLSTKGKFKVAVEIFAILASSYGLLGCLFLPKCFIILLRPKRNTDETVGGRVPTVDRSIQLTSASVSSELNSTTVSTVLDE
>Vmn2r1 .
MASRQISLALGFLAFLWAVLGAQNKTEEVQCRLMAKFNLSGYVDAKNHSLVIAGLFPIHSRIIPVDEAILEPVSPMCEGFNFRGFRWMKTMIHTIKEINE
RKDILPNHTLGYQIFDSCYTISKAMESSLVFLTGQEEFKPNFRNSTGSTLAALVGSGGSSLSVAASRILGLYYMPQVGYTSSCSILSDKFQFPSYLRVLPSDNL
QSEAIVNLIKHFGWVWVGAIAADDDYGKYGVKTFKEKMESANLCVAFSETIPKVYSNEKMQKAVKAVKTSTAKVIVLYTSDIDLSLFVLEMIHHNITDRT
WIATEAWITSALIAKPEYFPYFGGTIGFATPRSVIPGLKEFLYDVHPNKDPNDVLTIEFWQTAFNCTWPNSSVPYNVDHRVNMTGKEDRLYDMSDQLCT
GEEKLEDLKNTYLDTSQLRITNNVKQAVYAIAHGLDHLSRCQEGQGPFGSNQQCAYIPTFDFWQLMYYMKEIKFKSHEDKWVILDDNGDLKNGHYDVL
NWHLDDEGEISFVTVGRFNFRSTNFELVIPTNSTIFWNTESSRRPDSFCTQVCPPGTRKGIRQGQPICCFDCIPCADGYVSEKPGQRECDPCGEDDWSN
AEKSKCVPKLVEFLAYEEALGFTLVILSIFGALVLLAVTVVYVIHRHTPLVKANDRELSFLIQMSLVITVLSSLLFIGKPCNWSCMARQITLALGFCLCLSSILGK
TISLFFAYRISVSKTRLISMHPIFRKLIVLICVVGEIGICAAYLVLEPPRMFKNIEIQNVKIIFECNEGSIEFLCSIFGFDVLLALLCFLTTFVARQLPDNYYEGKCITF
GMLVFFIVWISFVPAYLSTKGKFKVAVEIFAILASSYGLLGCLFLPKCFIILLRPKRNTDETVGGRVPTVDRSIQLTSASVSSELNSTTVSTVLDE
>Rat_Vom2r47 .
MDSRKKCLILQLTLFLWVESSAQNKDQNVTCRLLRKFNLTGYVDSENHSMVIGGLFPVHYRTIPKIDPDEEPESAMCEGFNFRGFRWMKTMIHTIKEINE
RKDILPNHTLGYQIFDTCFSISKTVESTFVFLTGQEEYKPNFRNSSGKYLAGIIGSGGSSLSVASSRILEVYYMSQVGYTSSSSILSDHFRFPTFYRTIPSDKIQTE
AMVNLIKHFGWVWVGAIAADDDYGKYGIKSFKEKLERSNLCVAFSETIPKVYSNEKMQKAINAVKSSTAKVVVLYATDIDLRPFVLEVVHHNITDRTWIA
SEAWITSALIAKPEYFPYFGGTIGFAIPRSVIPGLKEFLYDVHPSKDPNDVLTIEFWQTAFNCTWPNSSVPYNVDYRGNMTGKEDRFYAMSDRLCTGEEKL
EDLKNTYLDTSQLRITNNVKQAVYLLAHALDRLSRCDVPQPDVKCSQIPNFDPFELMIYIMRMEFTTHDGRKIELDRSGDVKNGYYDILNWQMDDAGEI
AFVKFGEYKFTSSKYELVLSKNSTLFWNTESSELPDSFCTKLCPPGTRKGIRQGKPTCCFDCIPCADGYVSEKPGQRECDPCSEDDWSNAEKSKCVPKLVEF
LSYEEALGFTLVILSIFGALVVMAVTVVYVIHRHTPLVKANDRELSFFIQMSLVITVLSSMLFIGKPCNWSCMARQVTLALGFCLCLSSILGKTISLFFAYRISK
SKTRLISMHPIFRKLIVLICVVGEIGVCTAYLVLEPPRMFKNIEPQNVKIIFECNEGSIEFLYSIFGFNVLLALLCFLTTFVARQLPDNYYEGKCITFGMLVFFIV
WISFVPAYLSTKGKFKVAVEIFAILASSYGLLGCVFLPKCFIILLRPKRNTDETVGGRVPTLDKSIQLTSASVSSELNNTAESIVLDE
>Rat_Vom2r46
MASRKKCLILGLTVFLWLGLYAQNVNQNETCRLLRKFNLTGYVEAENHTVVIGGLFPVHYRTMPTSDSDEEIESPMCEGFNFRGFRWMKTMIHTIKEIN
ERKDILPEHTLGYQIFDNCFSTTKAMESALVFLTGQEEYKPNYKNSTGKYLVAIIGAGGSTMSVAVSRILQSYSVLQVGYASSSSILSDISKFPNTFRTIPSDKY
QSEAIVNLIIHFGWVWVGAIATDDDYGKHGVKLFREKMERSHLCVAFSETIPKVYSNEKMQIAVNEVKKSTAKVIVLYATDIHLSPFVLEVVHHNITDKT
WIASEAWITSALIAKPEYFPYFGGTIGFAIPRSVIPGLKEFLYDIHPSKDPNDLLTIEFWQTAFNCTWPNSSVSYNVDHRVNMTGKEDRLYAMSDQLCTGE
EKLEDLKNTYLDTSQLRITNNVKQAVYLIAYAMDRLSKADITEEYRENVVCPIIPDFESGELWNYFRNIKFTTHDGRKIETDFFGDIVRGYYDILNWHMDN
AGNIAFVKVGEYKFTTSKYELVLPKNSTLFWKTESYRVSYSVCTKVCSPGTRRKNRPGQPLCCFDCIPCEDGYVSEKPGQRLCDPCGEDNWSNPQKNKCV
PKLVEFLAYEEALGYALVILSIFGALVALAVTVVYVIHRHTPLAKANDQELSFLIQMSLVITVLSSILFIGKPYNWTCMARQVTLALGFCLCLSSILGKTISLFFA
YRISKSKTRLISMHPIFRKLIVLICVVGEIGVCTAYLMLKPPRMVKNIEPQNVKIIFECNEGSIEFLCSIFAFDVLLALLCFLTTFVARKLPDNYYEGKCITFGMLV
FFIVWISFVPAYLSTKGKFKVAVEIFAILASSYGLLGCIFLPKCFIILLRPKRNTDETVGGRVPTVDRSIQLASTSVSSELNTTVSTVLDE
>Rat_Vom2r45
MASRKKCLILGLTVFLWVELYAQNEEQNVTCRLLRKFNLTGYVEAANHSMVIGGLFPIHSRTIPTNDSDEEPVSAMCEGFNFRGFRWMKTMIHTIKEIN
GRKDILPNHTLGYQIFDTCFSVSKAMETALVFLTGQEENKPNFRNSTGKYLVGIIGSGGSSLSVAASRILGLYYMPQVGYTSSCSILSDKFQFPSFYRSIPSDK
IQSEAMVNLIKHFGWVWVGAIAADDDYGKYGVKSFREKMESANLCVAFSETIPKVYSNEKMQKAVKAVKSSTAKVIVLFTTDIDLSPFVLEVVHHNITDR
TWIASEAWITSALVAKPEYFPYFGGTIGFAIPRSVIPGLKEFLYDIHPSKDPKDVLTIEFWQTAFNCTWPNSTVPYNVDHRVNMTGKEDRLYAMSNQLCT
GEEKLEDLKNTYLDTSQLRITNNVKQAVYLMAYALDRLSNCDLLEEYSNSTACSHIPDFEPREMLPYFKKLKITTHDGTEIELDNNGDIENGYYDILNWHLG
NAGEVAFVKVGEYKFTSSKYELVLPKNSSLFWNTESSMLPDSVCTKLCAPGTRKGVRQGQPICCFDCIPCADGYVSEKQGQRECDPCGEDDWSNAEKSK
CVPKLVEFLAYEEALGFTLVILSIFGAIVVFAVTIVYVIHRHTPLVKANDRELSFLIQMSLGITVLSSMLFIGKPSNWTCMARQVTLALGFCLCLSSTLGKTISLF
FAYRISKSKTRLISMHPVFRKLIVLFCVVGEIGVCAAYLVLEPPRMFKNIESQNVKIIFECNEGSIEFLCAIFGFDVFLALLCFLTTFVARKLPDNYYEGKCITFG
MLVFFIVWISFVPAYLSTKGKFKVAVEIFAILASSYGLLGCIFLPKCFIILLRPKRNTDETVGGRVPTVDRSIQLTSASVSSELNSTTVSTVLDE

>Rat_Vom2r44
MASQKICLALGFLAFLWAELGAQNKTEELQCRLMAKFNLSGYVDAKNHSLVIAGLFPIHSRIIPVDESILEPVSPMCEGFNFRGFRWMKTMIHTIKEINAR
KDILPNHTLGYQIFDSCYTISKAMESSLVFLTGQEEFKPNFRNSTGSTLAAMVGAGGSSLSVAASRILGLYYMPQVGYTSSCSILSDKFQFPSYLRVVPSDKI
QSEAMVNLIKHFGWVWVGAVAADDDYGKYGVKTFKEKMESANLCVAFSETIPKVYSNEKMQKAINAVKNSTAKVIVLYTSDIDLSPFVLELIHHNVTDR
TWIASEAWITSALIAKPEYFPYFGGSIGFALPRTTIPGLKEFLYDIHPSKDPNDVLTIEFWQTAFNCTWPNSSVPYNVDHRVNMTGKEDRLYDMSDQLCT
GEEKLEDLKNTYLDMTQLRITNNVKQAVYAIAHALDLLSRCQEGYGPFGTNNACAYIPTFDFWQLMYYMKEIKFKSHEEKWVVLDDNGDLKFGHYDIL
NWQLDDSGEISFVTVGRFNFKTDMFELIIPTNSTIFWNTESSRRPDSFCTQVCPPGTRKGIRQGQPICCFDCIPCADGYVSENPGQRECDPCGEDDWSN
AEKSKCVPKLVEFLAYEEALGLTLVILSIFGALVVLAVTVVYVIYRHTPLVKANDRELSFLIQMSLVITVLSSMLFIGKSCNWTCMARQVTLALGFCLCLSSTL
GKTISLFFAYRISKSKTRLISMSPIYRKLIVLICVVGEIGVCTAYLVLEPPRMFKNIEPQNVKIIFECNEGSIEFLCAIFGFDVFLALLCFLTTFVARKLPDNYYEGK
CITFGMLVFFIVWISFVPAYLSTKGKFKVAVEIFAILASSYGLLGCIFLPKCFIILLRPKRNTDETVGGRVPTVDRSIQLTSASVSSELNNTAVSTVLDE
>Cavia_Vmn2r1 .
MANRKKYLVLGFLAFLWPASGTQGEEKEEQTCRLLGKFDLNGYVDAKNHTLVIGGLFPIHSRTIPANESILEPVSAKCEGFNFRGFRWMKAMIHTIKEINE
RKDILPNTTLGYQIFDTCFTISKAMESALVFLTGQEENKPNIRNSTGAYLAGIVGSGGSSLSIAASRILGLYYLPQVGYFSTCSILSDKFQFPSYIRTIASDKFQS
EAMVRLIQHFGWVWVGTIAADDDYGKYGVKAFKEQMESANLCIAFSETIPKVYSNEKMQKAVKAIRSSTARVIVLYASDIDLSPFVLEMVYHNITDRTW
IASEAWITSALIAKPEYFPYFGGSIGFAIPRTDIPGLKEFLYDVHPSKDPNDVLTIEFWQTAFNCTWPNISVPYNVDHRVNMTGKEDRLYAMSDKFCTGEE
KLEELKNTYLDVSQLRITNNVKQAVYAMAYALDQLSRCEEGQGPFMPENTCAYLPDFEPWQLMYYVKTLKFNTHDGKKIEIDQNGDVFGYYDILNWQL
DDNGEIAFVKVGEYKFAPDKYELVFKKNSTIFWNTESSRLPHSVCTDVCPPGTRKGIRQGEPICCFDCIPCADGHVSERAGQRECDQCGEDYWSNEQKS
MCVLKEVEFLAYDEALGFTLVILSIFGALVILAVMIVYVIYRQTPLVNANDRELSFLIQVSLIITVLSSMLFIGKPYNWSCMARQVTLALGFSLCLSSTLGKTISL
FFAYRISISKTRFISMRPIFRKIIVLISILVEIGICIAYLILQPPRVYKNMESQNIKIILECNEGSIEFLCSMFGIDAFLALLCFLTTFVARQLPDNYYEGKCITFGMLV
LFIVWISFVPAYLSTKGKFKVAVEIFAILASSYGLLGCIFAPKCFIILLRPKRNTDETVGGRVPAVDRSIQLTSASVSSELNNTTVSTVLDE
>Rabbit_Vmn2r1 .
MASRKKCLVLGFLAFLWAEWGTEGQEEEEQTCRLLGKFDLNGYVDAQNHSVIIGGLFPIHSRTIPANESILEPVSAKCEGFNFRGFRWMKTMIHTIKEIN
ERKDILPNTTLGYQIFDTCFTISKAMESALVLLTGQEENRPNYRNSTGAYLAGIVGSGGSSLSIAASRILGLFYLPQVGYASTCTVLSDKYQFPSYIRTIASDKF
QSEAMVKLIQHFGWVWIGTIAADDDYGKYGVKVFKEKLESVNLCIAFSEILPKVYSKEKMHKVVDAVRTSTAKVIVLYTSDIDLSPFVLEMVYHNITDRT
WIASEAWITSASIAKPEYFPYFGGSIGFAVPRSDIPGLKEFLYDIHPSRDPNDVLTIEFWQTAFNCTWPNSSVPYNTDHRVNMTGKEDRLHAMSDRFCTG
EEKLEDLKNTYLDVSQLRITNNVRQAVYAMAYALDRLSRCEEGRGPFIPGHGCAYIPDFEPWQLMFYMKTLKFTTHDGRKIQIDIAGDVTGYYDLLNWQ
LDDNGDIAFVKVGEYIFTQSKFEFMIRKNSTIFWNTESSKIPHSVCTDLCPPGTRKGIRQGEPICCFDCIPCADGHVSRTAGQRECEQCGEDYWSNAEKSE
CVLKEVEFLAYDEALGFTLVILSIFGALVVLAVTVVYVIYRHTPLVNANGRELSFLIQVSLTITVLSSLLFIGKPYNWSCMARQVTLALGFSLCLACILGKTISLFL
AYRISKSKTRLISIRPLYRKIIILISVLVEVGICTAYLVLEPPRVYKNMESQNIKIIFECNEGSVEFLCSMFGVDVFLALLCFLTTFVARQLPDNYYEGKCITFGML
VFFIVWISFVPAYLSTKGKFKVAVEIFAILASSYGLLGCLFAPKCFIILLRPKRNTDEIVGGRVPAIDKSIQLTSASLSSELNTTVSAVVGD
>Rabbit_Vmn2r2 .
MASRKKCLVLGFLAFLWAEWGTEGQEEEEQTCRLLGKFDLNGYVDAQNHSVIIGGLFPIHSRTIPANESILEPVSAKCEGFNFRGFRWMKTMIHTIKEIN
ERKDILPNSTLGYQIFDTCFSISKATESALVLLTGQEENRPNFLNSTGAYLAGIVGSGGSSLSVAVSRILGLYYLSQVGYASTCTVLSDKRQFPSYIRTIASDKF
QSEAMVKLIQHFGWVWIGTIAADDDYGKYGVKIFKEKLESVNLCIAFSEILPKVYSKEKMQKVVDAVRTSTAKVIVLYTSDIDLSPFVLEMVYHNITDRTW
IASEAWITSASIAKPEYFPYFGGSIGFAVPRSDIPGLKEFLYDIHPSRDPNDILTIEFWQTAFNCTWPNSSVPYNTDHRVNMTGKEDRLHAMSDRFCTGEE
KLEDLKNTYLDVSQLRITNNVRQAVYAMAYAMDRLSRCEEKGWPFFGKYKCSNLPNFEPWQLMFFIQRVKFTTHDGRKIHVDRNGDVTGYYDLLNW
QLDDNGDIAFVKVGEYIFTQSKFEFMIRKNSTIFWNTESSKIPHSVCTDLCPPGTRKGIRHGEPTCCFECIPCADGHVSRRAGQRECEQCGEDYWSNAEK
SECVLKEVEFLAYDEALGFTLVILSIFGALVVLAVTVVYVIYRHTPLVNANGRELSFLIQVSLTITVLSSLLFIGKPYNWSCMACQVTLALGFSLCLACILGKTISL
FLAYKISKSKTRLISIRPLYWKIIILISVLVEVGICTAYLVLEPPRVYKNMESQNIKIIFECNEGSVEFLCSMFGVDVFLALLCFLTTFVARQLPDNYYEGKCITFG
MLVFFIVWISFVPAYLSTKGKFKVAVEIFAILASSYGLLGCLFAPKCFIILLRPKRNTDEIVGGRVPAIDKSVQLTSASVSSELNNTTVSAVVAD

Full or near-full lenght V2Rs (families-ABD) identified in guinea pig (Cavia porcellus) and rabbit (Oryctolagus cuniculus).

>gp1 ENSCPOG00000026120
NYQNVLAFIFAIEEINKNPCLLPNISLGYEFHNFMHSHWRVLESSLILLTGQDEIPNYTCRRQSNSIAVLTGTSWATAAQIGTLLELYKFPQLTFGCFDPMLS
ENAEFSSLYQTAPKDTSLALGMVSLMLYFGWTWVGLVITEGQKGHQFLSDVKTEMNRNKVRVAFVKMISNAVLSVLAHAQQHDFLTRETSPVNVGIIY
YDTKSLNDVNYNIGQYRMTWTVWITNSQWHADMPGRNFILESFHGTLIFAKHHKEIRGFQKFIQAVNPSMYPEDNFLTLFWYDHFHCSVADSDCKLK
NCTPNASLAWLPGNRFDMTMNDDSYNIYNAVYAMAHALHEMLLQQVQRQSMGHREGIQISPWQLHHYLKNVQVNNPAGDQVNLDNKRKLDAEY
DIFNFWNFPEGLRLKVKVGSFSSHVLHNQQLSLSEDMIEWATGITETPRSVCSESCNPGFRKSSQEGKMACCFDCTPCPENEIANDTDMEECVKCPDH
QYADIHHKHCVQKSVTFLAYEDSLGRALAGTALSLTILTVAILGLFVKHCSSPIVKANNRSLSYILLLSLSFCFLCALLFIGRPNTATCILQQVTFGIVFTVAVSTI
LAKTITVVLAFKVTTPDRRIRQFLVSGTPNCIIPICSLIQFTLCGIWMGTSPPFIDRDAHFEHGFIIIVCNKGSLTAFYCMLGYLGLLALVSFTVAFLARNLPDTF
NEAKFITFNMLVFCSVWVTFLPVYHSTKGRVMVAMEVFSILASSAGLLGCIFAPKCYIILFKKDRCSLQGIKIKIHSGSK
>gp2 ENSCPOG00000026403
MLAFIFAIEEINRNPYLLPNISLGYESHNSLHSHGNILKNVLMLHAGQDEVPNYTCGRDSKSVALLTGTSWATEAQSGPLLELYKFPQFSLQLTFGSFDLVL
SEYDRFSSLYQMASRDTSLALGMVSLVIHFNWNWVGVIVTAGAKGLQFLSDVRAEMDGNRVCLAFVKTVTSDPLLYVANTELHDFLTKDTIGVNVIIIYH
DTENVNDLNHNVGLHLGTWRVWVTNSQWQADLTGKNFLRDSFHGTFIFSNHHKEISGFKKFIQTVNPSIYPEDIFIQQFWYSHFQCSLSDSGCALENCI
PNASLEWLPVNRFDPTMSDASYNIYNAVYAVAHALHEMLLKQIQKQPAGNLHSFLRNIQFNNTVGDQMNLEEKIKLNAKFDILNFWNFPEGLRLKVKV
GEFSPYVTHGQKLSLSDEMIQWAMGSTETPRSVCTESCTPGFRKTPQEGKASCCFDCITCSENEITNNTDMEQCVKCPDQQYANDQKNQCLQKSVTFL
AYKDTLGKALVSTALSLTILTAVVLGLFVKHRDTPIAKANNRTLSYTLLISLTFCFLCALLFIGRPNTATCILQQTTFAVVFTVAVSTVLAKTITVVLAFKVTAPG
KRMRQFLVSGAPNYVIPICSLIQLTLCGTWMGTNPPYIDTDAHSEHGHIIIVCNKGSLTAFYCVLGYLGSLALVSFTVAFLARNLPDTFNEAKFLTFSMLVF
CSVWITFLPVYHSTKGKVMVAMEVFSILASSAGLLGCIFVPKCYIILLRPEKNALHGFRDKTHSRR
>gp3 ENSCPOG00000027528
NYLNVLAFIFAIEEINRNLHLLPNVSLGYEFYNFLHGHWWIMESPLTLLTGQSEIPNYTCGRGSKAIVLLLGTPWATAAQIGPLLELYKFPQLTFGSFNPNLK
DSGQFPSLYQVASKDTTLALGMVSLMLHFTWTWVGLVITEGQKGLQFLSDVRAEMGRNRICVAFVKMIEVTLRAFFGNFQYNFQTREISPANVAIIYYD
SESLSDVNFHIGGYLVTWRVWVTNSQWHADMMGRNFILDSFHGTLIFSNHHEEIPGFKNFVQRANPSRYPENNFLSLYWFKNFHCSLSDSGCSLRSCP
PNASLAWLPVNHFDTAMSDVSYNIYNAVYAVAHAFHEMILHIAQIQSMNNGEGMAFSHWQLHPFLKNIHFNSTTGDQVNLDDKRKLETEYDILNFQ
NFPEGLRLRVKVGKFSPHASHGQRLSVSDHLIEWATEVTEIPQSVCSESCDPGFRKVTQEGKAACCFDCSPCSENEIANFTDAEQCIKCPDHQYANSQRN
NCVQKSITFMDYKDLSGKILAGTALSFTILTAAVLSLFVKHRDTPIVKANNRALSYTLLVSLTFCFLCPLLFIGRPNTATCILQQTTFAVVFTVAVSTVLAKTITV
VLAFKVTAPGRRMRQLLVSGAPNYIIPICSLIQLTLCGVWMGTNPPFVDTDAHSEYGHIIIMCNKGSLTAFYCVLGYLGFLALLSFTVAFLARNLPDTFNEA
KFLTFSMLVFCSVWVTFLPVYHSSKGKVMVAMEVFSILASSAGLLGCIFVPKCYIILLQPGRNSLHGLKYTMHS
>gp4 ENSCPOG00000027304
QNFLAFIFAIEEINKNPHLLSNMSLGYEFHNFLYSHWRIVESSLVLLTGQNEIPNYSCRRKSKPVAVLTETSWAASSQVSTLLELYRFPQVTFGSFDPMLRD
NGQFPSLYQVAPKDTSLALGMVSLMLHFSWTWVGLIITEGHRGVQFLSDVRAEMERNRICVAFLKMVSSAFESYFFRPLQHDIIARETSPVTVVIIYCDTE
TLNDINYLIGQNVGTWRVWVTNSQWHADMSGANFILNPFHGSFIFSNHHEEISAFKNFVQEANPFRYPEDTYLTMYWLNHFRCSISDSDCALRNCTPN
ASLAQLPMNRFDTDMTDESYNIYNAVYAVAHALHEMLFQQTQKLTIRNRGLSIFSPWQLHPFLRNIQFNSPAGEKMHLNDEKKLDAEYDILNFWNFPD
GLRLKIKLGTFTPHVPHGQQLSLTDNMIEWMTGITEAPKSVCSKSCNPGFWKTPQEGKAACCFDCTPCSENEIANDTDMEQCVKCPDHQYANTARIQC
LQKSVTFLAYEHPLGKMLAGTALSFTVLTAVVLGLFVKHRDTPIVKANNRALSYTLLISLTFCFLCSLLFIGRPNTASCILRQTTFAVVFTVAVSTVLAKTVTVV
LAFKVTAPGRRMRQLLVSGAPNYIIPICSLIQLTLCGVWMGTNPPYIDTDAHSEHGHIIITCNKGSLTAFYCVLGYLGSLALLSFTVAFLARNLPDTFNEAKF
LTFSMLVFCSVWVTFLPVYHSSKGKVMVAMEVFSILISSAGLLGCIFGPKCYIIFLRLDRNSLNNFRNKTRSGRKKP
>gp5 ENSCPOG00000020635
RLMPNNYQSYLAFTFAIEEINRNPQLLPNISLGYEFHNFLYSHWKILENSFILLTGQYKIPNYTCRRESKCAAVLMEMSWGTSAEIGPLLELYRFPQVTFGD
YDPTLTDRGQYHSLHQVAPKDTYLALGIVTLMFHFHWTWVGLIITADHKGLQFLSDVRAEIDRHGICVAFVKMVSTVTVSYLSVIQKHDIFTADTSSVNV
VVIYYDNDNRNDLHYNIVQSSVTWKVWVTNSQWHADLSGRNFVLDTFHGVLVFSHHHEEISGFKNFVKEATPFKYPEDSYLFMYWYKNFHCSFSEPD
CALENCTINASVAWLPPNSFDTAMSDGSYNIYNGVYAVAHALHAMLFEDVQRPPVKNRNVMMFPHWELHPFLKNIQFSNPSGEQVILNDRRKLDSEY
DILNFWNFPEGLRLKVKVGTFSPLAPNGQKMTLSENMIEWTTGITETPRSVCSESCIPGFRKSSQEGKAPCCFDCTPCAGNEITNDTDMEQCIKCPEHHY
ANAQQNHCLQKSVTFLAYEDPVGKALAGTALILTVLTAAVLGIFVKHQDTPIVKANNQALSYTLLISLIFCFLCSLLFIGHPNTATCILQQTTFGVVFTVAVST
VLAKTITVMLAFKVTTPGRRMRQFLVSGAPNYIIPICTLIQFTLCAVWMGTNPPYVDTDAHSEHGHVIIMCNKGSLTAFYCVLGYLSSLALVSFTVAFLVR
NLPDTFNEAKFLTFSMLVFCSVWVTFFPVYHSRRGKVMVAMEVFSILASSAGLLGCIFVPKCYIILLRPEKNSLTEIRGRTHF
>gp6 ENSCPOG00000019824
RIMPLNYQTALSFIFAIEEINRNPHLLPNISLGYEFHNLLPSYWRLLESVFILHTGQNEIPNYTCGKESKSVALLTGTSWATSAQIGPLLELYKFPQPMFSDNG
QFPSLYQVAPKDAFLVHGMVSLMLHFSWTWVGMFVAEGHKGLQFVSDIRGQADKSRVCIAFVKMISSSYASYFFHTEKNDIIAREASPVNVVIIYYDTD
SLNHVNHYIRLHLVTWKVWVTNSQWHADVAGINFILDTFHGLLIFSHHHGKISGFNEFIQKATPSRYPEDTILSLFWFQNFHCSVSEFDCSLKNCTPNASL
AWLPENIFDPVMSDWSYNIYNAVYAVAYALQEMSLQQIQMPPMEDGGTMIFYPWQLHPFMKNIQFTSPAGEQVNLDLTRKLDANYNILNLWNFPE
GLRLKVKVGEFVPHSPLGQHFTIYEHLIKWATEITELPHSSCSESCHPGFRKSLQEGKPVCCFACTPCLENEIANDTDIEQCVMCPEQEYANIQRTHCLQKS

VMFLAYEDPLGKTLAGIALSFTIITSAVLGLFVKHRDTPIVKANNRALSYTLLISLTFCFLCSLLFIGRPNTATCILQHTTFAVVFTVAVSTVLAKTITVVLAFKVT
APGRRMRQLLVSGGPNYIIPICSLIQLTLCGVWMGTSPPYIDTDAHSENGHIIITCNKGSLTAFYCVLGYLGSLALLSFTVAFLARNLPDTFNEAKFLTFSML
VFCSVWVTFLPVYHSSKGKVMVAMEVFSMLASGAGLLVCIFAPKCFIILFRPERNSLHGFRVNKYPEEK
>gp7 ENSCPOG00000023021
FTFAVEEINRNPHILPNVSLGYEFHNFIYSHWRILENSFLLVTGQHEIPNYTCRKENKCVAVLTENPWDTSAQIGPLLKLYKFPQVYTGSFDPTLSESGQYPL
LHQVAQKDTFLAFAMVSLILHFHWTWVGLLTTEGHKGLQFLSDVRTEMDRNRVCVAFVKIVSSITVSYFSVMQKHDILTADTSSVNVVVIYYDTGSLND
VSYNIVQNLMTWKIWVTNSQWQADLDGRNFILDSFHGVLVFSHHFQEISGFTNYVQEATPFKYPEDTYLFMYWYKNFHCSFSESDCALENCTPNASLA
WLPSNNFDTAMNDRSYNIYNGVYAVAHALHAMLFAEEQGPSMRNRQMKTFSPWQLNPFLKNTQFSNSAGDQVNLNNRRKLDSEYDILNYWNFPE
GLRLKVKVGTFSPHAPHGQKMTLSEDMIEWSTGVTEIPLSVCSQSCSPGFRKSPQEGKAACCFDCIPCANSEITNETDMEQCVKCLGHQYANTQRNQC
LHKIVTFLAYEDPLGKALAGTALSLTALTAAVLGLFVKHQDTAIVKANNRTLSYTLLISLLSCFLCSLLFIGHPNTATCILQQTIFAVAFTVAVSTVLAKTITVVL
AFKITAPDKRMRWLLVSGTPNFIIPICSLIQLTLYAVWMGTNPPYIDTDAHSEHGHIIIVCNKGSLTAFYCVLGYLGSLALVSFTVAFLARNLPDTFNEAKFLT
FSMLVFCSVWVTFLPVYHSSKGKVMVAMEVFSILASAAGLLGCIFVPKCYIILLRPERNSLTGIKNNILSVRKEPS
>gp8 ENSCPOG00000024706
YQTALSFIFAIEEINRNPHLLPNISLGYEFHNLLPSYWRLLESFFILHTGQNEMPNYTCGKESKSVALLTGTSWATSAQIGPLLELYKFPQVSFGSFNPMFGD
NGQLPSVYQVASKDTSLVHGMVSLMLHFSWTWVGIIVAEGHKGLQFVSDFRGEAEKSRVCIAFVKMISSSYASYFLHIEKNDIIAQEASPVNVVIIYYDTD
SLNHVNHYIQLHLVTWKVWVTNSQWHADVAGRNFILDTFHGLLIFSHHHEKISGFKDFIQKATPSKYPEDTFLSLFWFQNFHCSLSESDCSPKNCTPNAS
LAWLPENIFDPVMSDWSYDIYNAVYAVAYALQEMSLQQIQMPPMEDEGMFYPWQLHPFMKSIQFTSPAGEQVNLDLTRKVDANYNIVNLWNFPEG
LRLRVKVGEFFSHSQFGQYLAISEDLIEWATEITELPHSSCSESCHPGFRKSLQEGKPVCCFVCTPCLENEIANGTDIEQCVMCPDQEYANIQRTHCLQKSV
IFLSYEDPLGKTLAGIALSFTIITSAVLGLFVKHQDTPIVKANNRALSYTLLISLTFCFLCTLLFIGHPNTATCILQHTTFAVVFTVAVSTVLAKTITVVLAFKVTAP
HRSMRQLLVSGAPNSIIPICSLIQLSLCGVWMGTNPPYIDTDAHSEHGHIIIMCNKGSLTAFYCVLGYLGSLALLSFTVAFLARNLPDTFNEAKFLTFSMLVF
CSVWVTFLPVYHSSKGKVMVAMEVFSMLASSAGLLVCIFAPKCFIILFRPERNSLHGFRV
>gp9 ENSCPOG00000020266
RIMPLNYQTALSFIFAIEEINRNPHLLPNISLGYEFHNLLPSYWRLLESFFILHTGQNEMPNYTCGKESKSVALLTGTSWATSAQIGPLLELYKFPQVSFGSFN
PMFGDNGQLPSVYQVASKDTSLVHGMVSLMLHFSWTWVGIIVAEGHKGLQFVSDIRGEAEKSRVCIAFVKMISSSYASYFYQIEKSIIAQEESPVNVVIIY
YDTDSLNHVNHYIQLHLVTWKVWVTNSQWHADVAGRNFILDTFHGLLIFSHHHEKISGFKDFIQKATPSKYPEDTFLSLFWFQNFHCSLSEFDCSLKNCT
PNASLAWLPENIFDPVMTDWSYDIYNAVYAVAYALQEMFIQQIQMPPMEDEGMFYPWQLHPFMKIIQFTSPAGEQVNLDLTRKVDANYNIVNLWN
FPEGLRLRVKVGEFFSHSQFGQYLAISEDLIEWATEITELPHSSCSETCHPGFRKSLQEGKPVCCFVCTPCLENEIANGTDIEQCVMCPDQEYANIQRTRCL
QKSVIFLSYEDPLGKTLAGIALSFTIITSAVLGLFVKHRDTPIAKANNRALSYTLLISLTFCFLCTLLFIGHPNTATCILQHTTFAVVFTVAVSTVLAKTITVVLAFK
VIAPQRSMRQLLVSGAPNSIIPICSLIQLSICGVWMGTNPPYLDTDAHSEHGHIIIMCNKGSLTAFYCVLGYLGSLALLSFTVAFLARNLPDTFNEAKFLTFS
MLVFCSVWVTFLPVYHSSKGKVMVAMEVFSMLASSAGLLVCIFAPKCFIILFRPERNSLHGFRVNKY
>gp10 ENSCPOG00000024100
RWFWKNYQYVLAFYFAIKEINKDSQLLPNVTLGFHVYNAITSDCFALWSTLHWLCGTEIPLPNYNCQTYRKFAAVIAGTSAAFSAETGTILELYKFPQVTY
GPFDPILSDRHQYPSIYQMAPKDSTLVHAMISLLLHFGWTWVAIFVSDDVKGEKFLGDLKAEMLKKGICVALTEKLPATKIMYASSDITFMSKIRVSSANV
HILYGEVSSLITVDIAAEFFLTTGKVWIMTAKWDIVVYEANHMLHSFHGSFSFSPHKGEVPGFRHFLQTTTPSQYPEDFYFSKLWLNFFDCSLPGSQCGRI
GVCPPNTSLEFMPGNTNLMTLSDSSYFIYNAVYALAHVLHKMLLEKAEFASSEDAEQPGLLPWQLHPHLKKIEFTNSAGYSISLDEERQHVAQYDIQNTL
NFPGGLSVLAKVGEFFFKRPHGQNLVINDELIEWPVGFTETPQSVCSQSCGPGFSKISQEGRPVCCFTCIFCPERHISNQTDAEQCTQCPEHEYTNRNRD
RCLPKILDFLSFEDSLGMALTHIALCFSVLTAVILAIFVKHRDTAIVKANNRTLSYTLLISLLLCFLCCLLFIGRPNTATCILQQMTFGLVFTVAVSCVLAKTITVV
LAFKVTGPEKRMRQLLLLGAPNYIIPICTLIQLTLCGFWMGTNPPYIDTDAHSEHGHIIIVCNKGSLTAFYCVLGYLGSLALLSFTVAFLARNLPDTFNEAKFL
TFSMLVFCSVWVTFLPVYHSSKGKVMVAMEVFSILASGVGLLGCIFLPKCYVILIRSENNTLTGLKS
>gp11 ENSCPOG00000020354
IEQINKNPHLLPNISLGYEFHNFPYSHWRMLETSLILLTGQNDIPNYSCRRKSKSVAVLTGTSWAASAQVGTLLELYKFPQVSFGSFDPVLRDNGQFPSIYQ
AAPKDTSLALAMVSLLLHFRWTWVGLVIMEGQKGFQFLSDVRVEMDRNKICVAFVKMVSTHLVSYMASAKEYIILTKETSQVNVVIIYYDTDFLNDVNH
NIEQNLLTGKVWVTNSQWHADMTGKNFILNSFHGTLIFSNHHEEISDFKTFVQEANPSKYPEDFYLTRFWFSNFHCSFSDADCSLKDCTPNASLVHLPA
NHFDPTMTDWSHNVHNAVYAVAHALHEMLLQQAQMSLMGNKKVTIFSPWQLNPILKNIQFNNPAGDQVNLNDRSKVDVQYDILNFWNFPEGLRL
KVKLGAFNPHVPHDQQLSLSDMIEWATGITEMPYSVCSESCHHGFRKTSQEGKPACCFDCTPCPENEITNDTDMEQCVKCSDDHYATIQQDLCLQKSV
TFLAHEDLMGKMLAGTALSLTVLTAAVLGLFVKHQDTPIVKANNRALSYTLLISLIFCFLCPLLFIGQPNTATCILQQTTFAVVFTVAVSTVLAKTITVVLAFK
VTAPGRRMRQLLVSGAPNYIIPICTMIQLTLCGVWMGTNPPYIDTDAHSEHGHIVIMCNKGSLAAFYCVLGYLGSLAMLSFTVAFLVRNLPDSFNEAKFL
TFSMLVFCSVWVTFLPMYHSSKGKVIVAMEVFSILVSGAGLLGCIFAPKCYIILLRPERNSLHGFRDKTRS
>gp12 ENSCPOG00000023880
KNYQYVLAFYFAIEEINKGSQLLPNVTLGFQVYNAIASDHFALWSTLHWLCGTDIPLPNYNCQTHRKFAAVIAGTSAAFSAETGTILELYKFPQFFQVTYGP
FDPILSDKHQYPSVYQMAPTDSTLVHAVISLLLHFGWTWVAIFVSNDVKGEHFLGDLKAEMLKKGICVALTKKLPATKIMYASSDITLMSKIRVSSANVHI
LYGEVASLINVDIAAEFFLTTGKVWIMTAKWDIVVYETDHMLHSFHGSFSFSPHKGEVPGFRHFLQTTKPSQYPEDFYFSKLWLNFFDCSLPGSQCGRIG
VCPPNTSLEFMPGNIDLMTLSDSSYFIYNAVYALAHVLHKMLLEKAELHPHLKKMEFTNSAGFSIFLDEERQHVAQYDIQNTLNFPGGLRMLVKVGEFFF
KRPQGQSLVVNEDLIEWPVGFTETPPQSVCSHSCGPGFSKIPQEGRPVCCFTCIFCPERHISNQTDAEQCIHCPKHEYPNRNRDRCLPKILDFLSFEDSLG
MALTHVALSFSVLTVVIMAIFVKHRDTAIVKANNRTLSYTLLISLLLCFLCSLLFIGRPNTATCILQQMTFGLVFTVAVSSVLAKTITVVLAFKVTGPGRRMRH

LLVSGAPNYIIPICTLIQLTICGFWMGTNPPYIDTDAYSEHGHIILLCNKGSLTAFYCVLGFLGSLALLSFTVAFLAKNLPDTFNEAKFLTFSMLVFCSVWVTFL
PVYHSSKGKVMVAMEVFSILASSAGLLGCIFLPKCYVIFIRSENNTLTGLKTRKNVRGNRY
>gp13ENSCPOG00000020639
IEKINKNPHLLPNISLGYEFHNFPYSHWKMLESSLILLTGQSDIPNYSCRKKNKSVAVLTGTSWAASAQVGTLLELYKFPQVTFGSFDPVLGDNGQFPSIYQ
AAPKDTSLVLAMVSLLLHFRWTWVGLVIMEGHKGLQFLSDVTGEMDRNKICVAFVKMVSTHLVSYMAAAKEYIILTKETSQVNVVIIYYDTDFLNDVNY
YIEQNLLTGKVWVTNSQWHADMTGKSFILNSFHGTLIFSNHHEEISDFKMFVQEVNPSKYPDDFYLTRFWFNNFHCSFSDADCSLKDCSPNASLVQLPA
SHFDTTMTDWSHNVHNAVYAVAHALHEMLLQQAQMSLVENKKVTVFSPWQLNPILKNIQFKNPAGDQVNLNDKSKVDGQYDILNFWNFPEGLRLK
VKFGTFTPHVPQGQRLSLSDDMIEWATGITEMPHSVCSESCQHGFRKTSQEGKPACCFDCTPCPENEITNDTDMEHCVKCPDDKYATIQQDHCLQKSV
TFLAHEDLIGKMLAGTALSLTVLTAAVLGLFVKHQDTPIVKANNRALSYTLLISLTFCFLCSLLFIGRPNSATCILQQITFAVVFTVVVSTVLAKTITVVLAFKIT
APGKRMRQLLISGAPNYIIPICTLVQLTLCGVWMGTNPPYIDTDAHSEHGHIIILCNKGSLTAFYCVLGYLGSLALLSFTVAFLVRNLPDTFNEAKFLTFSTLV
FCSMWLTFLPIYHSSKGKVMVAMEVFSILVSGAGFLGCIFAPKCYIILLRPERNSLHHFRDKTHSSK
>gp14 ENSCPOG00000027402
RLTPSNYQNYLAFTFAVEEINRNPHLLPNISLGYEFHNFIYSHWRILENSFILLTGQHEIPNYVCGKKSKCSAVLTDIPWDTSAQIGPLMELYKFPQDPTFTE
SGQYPSLHQVAQKDTHLAFGMVSLMLHFHWTWVGLLTTEGHKGLQFLSNVRTEMDRNRICVAFVKIVSSATVSYLSVMQKHDIWTADTSSVNVVVIY
YDTDSQNDVSYNIVQNLMTWKVWMTNSQWQAVLDGRNFILDSFHGVLVFSHHHEEISGFKTYVQEATPFKYPGDTYLFVYWYKNFHCSFSESDCTLK
NCTPNVSLAWLPLNHFDTAMSDRSYNIYNGVYAVAHALHVMLFGEEQGPPMRNRKMMMFSSWQLHPFLKNIQFSNPAGDQVNLNDRSKLDSEYDI
LNFWNFPEGLRLKVKVGTFTSHGPHGQKMTLSEDMIEWTTGVTEIPLSVCSESCHPGFRKSPQEGKAPCCFDCIPCADNEITNETDMEQCAKCLGHQY
ANTQRNHCLHKTVTFLAYEDPLGKVLAGTALSLTVLTAAVLWLFVKHQDTPIVKANNQALSYTLLISLLSCFLCSLLFIGHPNTTTCILQQTIFAVAFTVAVST
VLAKTITVVLAFKITAPGKRMRQLLVSRTPNSIIPICSLIQLTLCGIWMGTNPPYIDRDTHSVHGHIIIVCNKGSLTAFYCVLGYLGSLALVSFTVAFLARNLPD
TFNEAKFLTFSMLVFCGVWVTFLPVYHSSKGKEMVAMEVFSILASGAGLLVCIFVPKCYIILLRPERNSIIRIRNKTHS
>gp15 ENSCPOG00000025728
FYYAIEEINKDPHLLPNQSLGFQLYNAINSDHRTLENALIWFSGGDEILPNYSCKIPKRSVAVTAGTASPFSAQIGTLLELYRTPQITYGPFDPSLRDKHQYPS
LYQMAPGDSNLVHAMIALLLHFGWTWVAVFISDDVKGEQFMRDLEAEMLKKGICVALTEKLPAITTMFGSSHTTFMTRIRVSSANVHILYGEVRSLITV
DIAAEFFLTTGKVWIMTAKWDMVVYETDHMLHSFHGSFSLSPHKREIPGFKHFLKTSNPSQYPQDFYFSILWIAFLNCSPPGSLCGKIGVCLPNISLETLPG
NFDLMTVSDSSYFIYNAVHVLARVLHTVILEKMEMRSPEDENQPEILPWQLHPLLKKIQFINSGGHQMSFNEKRHRMAWYDIQNNVNFPAGLRLLIKV
GEFVSKSPHDQALVINAEMVEWPIGFTETPKSVCSQSCAPGFRQIPREGRPLCCFHCVLCPERHISNRTDAQHCVQCPPKEYANSERNHCLPKVITFLSLQ
DPLGMSLACTALCFSVITTLVLGLFVKHRDTPMVKANNRGLSYTLLISLTFCFLCSLLFIGRPCTASCILQQTTFAVVFTVAVSTVLAKTITVVLAFKVTAPGR
NMRQLLVSGAPNYIIPICTLIQLTLCAVWMGTNPPFVDTDAHSEHGHIIIMCNKSSLTAFYCVLGYLGSLALLSFTVAFLARKLPGTFNEAKFLTFSMLVFCS
VWVTFLPVYHSSKGKVMVAMEVFSILASSAGLLGCIFLPKCYIILIKPDKNSMKYLRSRK
>gp16 ENSCPOG00000026305
FLFAIEQINKNPHLLPNISLGYEFHNFPYSHWKMLESSLIFHTGQSDIPNYSCRRKNKSVAVLTGTSWAASAQVGTLLELYKFPQVTFGSFDPVLGDNGQF
PSIYQAAPKDTSLVLAMVSLLLHFRWTWVELVIMEGHKGLQFLSDVTGEMDRNKICVAFVKMVSTHLVSYMAAAKEHIILTKETSQVNVVIIYYDTDFLN
DVNHYIEQNLLTEKVWVTNSQWHAGMTGKSFILNSFHGTLIFSNHREEISDFKMFVQEVNPSKYPDDFYLTRFWFNNFHCSFSDADCSLKDCSPNASLV
QLPASHFDTTMTDWSHNVHNAVYAVAHALHEMLLQQAQMSLMENKKVTVFSPWQLNPILKNIQFKNPAGDQVNLNDRSKVDVQYDILNFWNFPE
GLRLKVKLGTFTPHVPQGQRLSLSDDMIEWATGITEMPRSVCSESCQHGFRKTSQEGKPACCFDCTPCPENEITNDTEDMEHCVKCPDDKYATIQKDH
CLQKSVTFLAHEDLIGKMLAGTALSLTVLTGAVLGLFVKHQDTPIVKANNRALSYTLLISLTFCFLCSLLFIGRPNSATCILQQITFAVVFTVVVSTVLAKTITV
VLAFKITATGRSMRQLLISRAPNYIIPICTLVQLTLCGVWMGTNPPYIDKDAHSEHGHIIILCNKGSLTAFYCVLGYLGSLALLSFTVAFLARNLPDTFNEAKF
LTFSMLMFCSMWLTFLPVYHSSKGKVMVAMEVFSILVSGAGLLGCIFVPKCYIILLRPERNSLHHFRDKTHSSK
>gp17 ENSCPOG00000023273
YQYVLAFYFAIEEINKDSQLLPNVTLGFHVYNAIASDHFALWSTLHWLCGTDISLPNYNCQTHRKFAAVIAGTSAAFSAETGTILELYKFPQVTYGPFDPILS
DKYQYPSVYQMAPTDSTLVHAMVSLLLHFGWIWVTIFVSDDVKGEHFLGDLKAEMLKKGICVALTEKLPATKIMYASSDITFMSKIRASSANVHILYGEV
GSLITVDIAAEFFLTTGKVWIMTAKWDIVVYETNHMLHSFHGSFSFSPHKGEIPGFRHFLQTIKPSQYPEDFYFSKLWLTFFDCSLPGSQCGRIGVCPPNTS
LEFMPGNIDLMTLSDSSYFIYNAVYALAHVLHKMLLQKAELGSSEDAEPPGLFPWQLHPHLKKVEFTNSAGYSIFLDEERQHVAQYDIQNTLNFPGGLSV
LVKVGEFFFKRSHGQSLVINEDLIEWPIGFTETPPQSVCSQSCGPGFSKIPQEGRPVCCFTCIFCPERHISNQTDAEQCTQCPKHEYTNRNRDRCLPKILDFL
SFEDPLGMALTQIALSFSVITAVILAIFVKHRDTAIVKANNRTLSYTLLISLLLCFLCSLLFIGRPNTATCILQQMTFGLVFTVAVSSVLAKTITVVLAFKVTGRR
MRHLLVSGAPNYIIPICTLIQLTLCGFWMGTNPPYIDTDVHSEHGHIIIVCNKGSLTAFYCVLGYLGSLDLLSFTVAFLARNLPETFNEAKFLTFSMLVFCSV
WVTFLPVYHSSKGKVMVAMEVFSILASSAGLLGCIFLPKCYIILIRSENNT
>gp18 ENSCPOG00000023032
RLTLSNYQNYLSFIFAIEEINKNPHILPNISLGYEFHNFIYSHWRMLENSFILLTGQHEIPNYTCSRQSKCVAVLTEMSWGTSAEIGPLLELYRFPQITFGSFDS
TLTDKVQYPSVYQVASKDTYLALGVVSLVLHFHWTWVGLLITEGHKGLQFLSDVRAEMDRNRVCVAFVKMVSTVTVSYLSATQKHHILTADTSSVNVV
VMYYDTDSQTDANYNIWQHSVTWKVWVTNSQWHPDLVGRNFILDPFHGTLIFSHNHKEISGFKNFVQGATPFKYPEDIYLIMYWYNNFHCSFSEPDC
ILKNCTPNTSLAWLPLNRFDTAMSDGSYHIYNGVYAVAHALHAMVFEEVQCPPMRNRQMMVFSTWQLHPFLKDIQFNNPVGDQVNFDHSRKLDSE
YNIHNFWNFPEGIRHKVKLGTFSSHAPYGQRMSLSEDMIEWAIGITETPQSVCSDSCNLGFRKSPQEGKAACCFDCIPCADNEIANDTDMEHCIKCSDH
EYANTQQNRCLKKAVTFLAYEDPLGKALAGTALSLTVLTAAALGIFVKHRDTPIVKANNPAMSYTLLISLIFCFLCSLLFIGRPSTVTCILQHTIFAIVFTVAVST
VLAKTITVVLAFKATAPGRRMRQLLMSGALNYIIPICTLVQLTLCGIWMGTHPPYIDTDAHSEHGHIIIMCNKGSLRAFYSVLGYLGSLALLSFTVAFLARNL
PDTYNEAKFLTFSMLVFCSVWITLLPVYHSRKGKVIVAMEIFSILASSAGLLGCIFVPKCYIMLFRKEKYSVYSVKHKTHF

>gp19 ENSCPOG00000024688
ILKSYQNVLAFIFAIEEINRNPHLLPNITLGYESHNSLHSHGNIVQNILIVQTGQDRIPNYMCGRESKSLALLTGTSMATAAQSGPLLELYKFPQLTFGSFDPI
LSESDQFSSLYQMASEETALAQGMVSLMLHFSWIWVGVAITEGPKGLRFLADVTKEMDRNRVCMAFVKMIPVDPVSYMTHTEQHELLVGESSAVNV
VIIYYDADSLNDINYSISLHLVTWRVWVTNSQWHADLTGKKFLLDSFHGTIIFSNHHEEISGFKHFVQTVNLSMYPEDIFLYQFWYWNFNCLISESDCSLK
NCTPNASLAQLPANRFDPAMSDVSYNIYNAVYAVAHALHEMTLQQVQMQNTGSGERRTFSPWQLHPFLRSIQFTNPAGHHVSLDKKIKLDVKYDILN
FWNFPEGLRLKVKVGEFSPYAIHGQQLSLFQEMIQWATGHTETPHSVCSDSCNPGFRKSPQEGKAACCFDCTPCPENEITNDTDMKNCMKCPDHQYA
SIQRNQCLPKAVTFLAYEDSMGMTLTGTALSLTSLTAAVLVLFVKHQDTPIVKANNPALSYTLLISLMFCFLCSLLFIGCPNTISCILQQTTFGVVFTVSVSTV
LAKTVTVMLAFKFTVPGSRMRQLLVSGAPNYIIPTCTLIQLTICGVWMGTSPPFVDTNAHSEHGHIIIVCNKGSLTAFYFILGYLGSLSLISFIVAFMARNLP
DAFNEAKFLTFSMLVFCSVWVTFLPVYHSSKGKVMVTMEVFCILASSSGLLGCIFFPKCYIILLKPERNSLHEFRN
>gp23 ENSCPOG00000025441
MKSHNIFPLLVALLCIFQKRSSTHSLNAPGSPGYYQDGDFIIGGLFSLRVISGDLRSKFGFQHEVYIPPFAYAHLTKHYQQVLAMVFAVNEINKKLNLLFNM
SLGFYIFNVDYIEMKAVESSLSLLSCESPPVPNYNCRPEKRDKLVAVIGGISTGISTQISRVVSLYSVPQISYGPFDHSLKDSVQLQSIYQFPMNTAALYQGLI
QLMLHFGWVWVGFVVPDDIRGDKFLQDMTQEMNNNGLCIAFAERIPEFPADDTINMQHFFERYTTTRVSVAFGDTYSLLRYVYHIYCSSLFGNILVTTL
DWDITTIPFQQGPSYTYFGGGLSFSFHIEEIPGFKTFLRSVQPTKYPHDVFIQHVWSILFECLYMNQNGIMTFTDCKENGTLETRPLHVWDMNTSPQSYN
VYAAVNVIAWALHDLLSIRTEEEPPDNVTYVNSHPWQLHPFLEKSQLERSNSKQKISSEKLSTTSLDIFNYQSLLNGTKAQVKVGEFIFQPHKAPHLSFNDK
LMMWGEHHNEAPSAICNPSCQIGFRKTAVKGKSSCCFDCIPCPGGEVANKTDMHQCVKCPENQYPNMERYQCLPKVMTFLSYDDTLGTALASTAICL
SVLTALILGLFIQYRNAPIVRANNRNLSYVLLISLILCFFCSLMFIGRPSPVTCILRQTFFGVVFSIAVSAILTKTFIVLVAFKSIKPGSSIQMWMVTRISNAIVYIC
SLIQGCICTVWLATSPPFPDTDTQSEFGQIILHCNEGSTVAFYCVLGYLGFLAFLSLVLAFLGRRLPDKFNEAKMITFSMLVFCSVWISFIPSYLSTKGKTIVAL
EIFAILSSSAGLLGCIFLPKCYLILVRPENNSRKKLHEPFT
>gp20 ENSCPOG00000026642
WGYRVAQSFVFAVEEINRSTHLLPNLTLGFSIHNSGDSVHGAMHETMSFLSRQEDPVPNYACHWGPPRVAMVGDTRSALSVAMARLLGLYKFPQVSH
SSTLPSLSDKTQFPSFLRTRASDFVSSLAVIQLVLSLGWSWVIILAQDDEFGQQAGALAARELEKAGICIEFHLTVPSQHSLEKITCVVQKMQTSTATGVLV
FLNNSNFRLILQSLLGFHPLGQVWVSEGLLHTALALATPGISQILQGAFGLLCHNSQVQGFPEFFSSLHPSQIPEDVFLVRFWEATFGCKWPHENSTETED
VRLCTGAESLRGHEFPFQDVSEVDIAYTAVYSVAHALQDLVSCEHGSGVCTDLQHLHPWQLLHPLRKVHFQTHDGTDIMFDANGDLVTKFDVLQGQW
NVEGQFQFSHIGTLDPHSSLGNSMMILLKDSVEVPSSVCSTSCEPGFSQIPRQGVPHYCFECHPCPEGQFADLLYMKSCLQCPEKQYPSQTRDHCIPRTEI
FLTFDEPLGLALTLVSLALASLACLVFGVFLWFRHTPVVRANNRALSYVLLVSLTLCALSPLLFLGRPSPTTCLLRQTTFAVVFTVAVSCVLAKTLTVDLAFRA
TRPGQRVRMCLGNSASSSVVLVSSLMQIILCGVWLGTSPPFPHRDTASEPGHLVLQCQEGSGMAFSCMLGYLGLLAIGTFSVAFLARDLPDAFNEAKFL
TFSMLLFCSVWTAFLPLYHSARGKATVAVEVFSILASTAGLLGGIFLPKCYLILLKPEQNAPTKLQIARQTQCAAVAPTNMCTPMFLAQRP
>gp21 ENSCPOG00000022627
VSSWGYLVAQSFVFAVEEINRSTHLLPNLTLGFSIRNSGDSVHGAMHETIAFLTGQEDPVPNYSCHRGPPRAAMVGDTRSALSVAMARLLGLYKFPQVS
YASSLPTLSDKTQFPSFLRTRPSDLTASYAMTQLVLHLGWSWVGIITQDDFGQQAGNIASQELGQAGVCIDFHLHVPYHQSPEKIDTIARKMEKSTARGV
LVFLTNSIIQLIIQRLVGIRVLGQVWVSETLLHTVIALATPGASWVLQGAFGLKLHSSHAHGLSEFFAHLHPSRTPEDMFLRRFWEATFGCRWPHGNYTA
PSDMQLCSGNETLRDHKYPFQEVSRLEAAYPAVYSIAHALQDMVLCEHENSECGDPVHFQPWQLLQPLRKVSFQTPDGTHIMFDANGDLVTKFDVLQ
GQWSPEGQFYFVHIGNMYPHPSLENRMMILLKDSIQVPSSVCSTSCEPGSSQIPQQGAPHCCFECRPCPEGQFADQRDMKSCLQCPDEQYSSQSKDH
CLPRTETFLAFDEPLGLVLTVVSLALASLACLILGVFLWFQKTPVVRANNRALSYVLLVSLSLCALSPLLFLGRPSTTTCLLRQITFAVVFTVAVSCVLAKTLTV
VLAFKATRPGQRVRICLGPFASSSVVLAASLVQIILCGVWLGTSPPFPHKDTASEPGHLVLQCHEGSGIAFSCVLGYLGLLAIGTFSVAFLARGLPDTFNETK
FLTFSMLLFCSVWTAFLPLYFSARGKATVAVEVFCILASTAGLLGGIFFPKCYLILLKPERNTPLSLRHEPWAQRDLGKGDPPSHRHSTISARPPSQQQDPG
SQFLVPVLTTW
>rb93 ENSOCUG00000022413
VSRWGYRVAQGFVFAIEEINRDTHLLPNLTLGFSIRNSGDSVHGALHETLGFLTGQEEPIPNYACGFSPPQAALIGDTRSALSVSMARLLGLYKFPQVSYSS
TLPSLSDKAQFPSFLRTRASDLTSSHAVSQLVLHFRWSWVGILAQDDDFGQQASSLVARELGQAGVCLEFHLHVPSQQSLEKTEALVQKMASCTATVVL
VFLSNSNFQLLLLGLQGVGVSGQVWVSKDVLHLGLALTVPGVSRVLQGSFGLMLQVSQALGFPEFLARLHPSRTPEDAFIERFWEVTFGCKWPPRNSTA
SGSTRLCSGNESLRGHEHPFQEVSKVDVAYSAVYSIAHALQALGDCVHEDGACADPLHFQPWQLLHPLRKVHFKTPDGKEIMFDSNGDLLTKFDILYGQ
KTANGLFRFVHIGVIDPGATSEKRMTVHLMKEDLQVPSSVCSRSCAPGFSQIPRQGAPQCCFDCSPCPEGQFADQRDMKRCLLCPKEQYSSPTRDRCLP
RTETFLAFDEPLGLTLASVVLLLAGLAVLVLGVFLKHSDTPVVRANNRALSYTLLTSLALCALCPLLFLGRPTTATCLLRQTTFAVVFTVAVSSVLAKTLTVVLA
FRAIRPGVRVQVCLGPNASSSVVLTASLVQVVLCGVWLGTSPPFPHRDVASEPSQVVIYCQEGSGVGFYCMLGYLAVLAGATFCMAFLARGLPDAFNET
KFLTFSMLLFCSIWTAFLPLYHSSRGKSTVAVEIFSILASTAGLLGGIFLPKCYIILLKPERNTLACIRHGRQAQQGRGRARSCRVAVSLGPH
>rb1 ENSOCUG00000024215
MFPLNFLLLFLQLCLLVTCHDDQNCFFQMERSYYKDGDIEIAAFFPIYIYLVDRMINIFKNHVTFKGFQSKNYQYVLALVFAIEEINKNPQLLPNVTLGFDLY
NVMHSDMMVMQNPFIWLAGLEEDVPNYMCRKWSKSVAVISGTSIAVQVGTLLELYKIPQLTLGSFEPLLSDSGQFPSLYQMAPKDTSLALGIVSLMLH
FSWTWVGLAISDLPKGIQFMSNLKVELQKNGICVDFVELIAVTEESYISLQNRFHIQILKSSANVVILFCDTDSLIGISFPTWERVMTWKVWVTTSQWDFA
SDEHHILLHSFHGTLIFSHHHGEISGFKNFLQTANPSKYPEDIYLLRFWLLVFDCSVTGASCKTLQNCPLNASLESLPPHRFDMSMSDGSYNIYNAVYAVA
HSVHEMLLQLLEIQPVNNGAKLTFSPWQLHPFLKNLQFTNPVGDLVNLNHMTNLEAEYDILNFWNFPYGVGRKVKVGQFSPYASQSQKLSLSENLIEW
AIGITETPRSVCSESCSPGFRKASLEGKPTCCFDCTPCPENEISNQTDMDQCMKCLDHQYASTERNQCFHKKVTFLSFEDPLGMTLVCTALCFSVLTAVVL
GVFVKHRDTPLVKANNRCLSYILLIALIFCFLCSLLFIGHPNTTTCVLQQTTFGVMFTVAVSTVLAKTITVVLAFKVTAPGRRMRHWLISGVPNSIIPICSLIQL

ALCAIWLGNSPPFIDTDAHSEHGHIILMCNKGSVIAFYCVLGYLGSLAMASFTVAFLARNLPDKFNEAKFLTFSMLVFCSVWVTFLPVYHSTKGKVMVAV
EVFSILASGAGLLGCIFVPKCYIILLRPEKNALKGLRDRRISKETDVL
>rb2 ENSOCUG00000021793
MFPLIFLLLFLQLCLLVTCHDDRNCFFQMERSYYKDGDIEIAAFFPIYIYLVDRMINIFKKDFTFKRFQSKNYQYALALVFAIEEINKNPQLLPNVTLGFDLYN
VMHSDMMVLQNSFIWLAGLEEDVPNYMCRKQSKSVAVISGTSIAVQVGTLLELYKIPQLTLGSFEPLLSDSGQFPSLYQMAPKDTSLAHGLVSLMLHFS
WIWVGLAISDLPKGIQFMSDLKVELQKNGICVDFVELIPVTEESRISLQNRFHIQILKSSANVVILFCDTDSLIGISFPTWERVMTWKVWVTTSQWDFASD
ERHILLHSFHGTLIFSHHHSEISGFKNFLWTANPSKYPEDIYLLRFWSLVFDCSVTGASCKTLRNCPLNTSLELLPLHHFDMSMSDGSYNIYNAVYAVAHSV
HEMLLQLLEMQPGNKGEKVAFSPWQLHPFLKNLQFTNPAGDLVNLNHLRNLEAEYDILNFWNFPYGVGRKVKVGQFSPYASQSQKLSLSENLIEWAT
GITETPHSVCSETCSPGFRKAPLEGKPTCCFDCTPCPENEISNQTDMDQCVKCLDHQYANRERNQCFYKKLTFLSFEEPLGMILVCTALCFSVLTALVLGVF
VKHRDTPIVKANNRCLSYILLIALIFCFLCSLLFIGRPNTTTCVFQHTTFGVVFTVAVSTVLAKTITVLLAFKVTAPGRRMRHWLISGVPNSIIPICSLIQLALCAI
WLGTSPPFIDTDAHSEHGHIILVCNKGSVIAFYCVLGYLGSLALASFSVAFLARSLPGTFNEAKFLTFSMLVFCSVWVTFLPVYHSTKGKVMVAVEVFSILCS
SAGLLGCIFVPKCYIILLRPEKNALKGLRDRRIS
>rb3 ENSOCUG00000021686
TLKMFPWIFLLLFLQLYLLVICQDGPKCYSEIERSFYKDGDIEIASFFPIYIHLVNRIMNIFQRDLTFIRFQSKNYQYVLALVFAIEEINKNTHLLPNMTLGFDLY
NVMHSDMMVMENPFIWLAGMEKHVPNYMCRKQSKSVAIISGTSIAAQMGTLLELYKIPQLTLGSFEPLLSDSGQFPSLYQMAPKDTSLAHGMVSLML
HFSWTWVGLAISDLPKGIQFMSDLKVEMQKNGICVDFVEFIPVTEESHNSFQRLYHIQILKSSANVVILFCDTDSLIGVSFPTWQCVMTWKVWVTTSQ
WDFASDEHHILLHSFHGTLIFSHHHSEISGFKNFLQTVNPSKYPEDFYLSTFWSLYFDCSVTGPSCKTLRNCPLNASLESLPFHHFDMSMSDGSYNIYNAV
YAVAHSVHEMLLQELEMQPVSSGSKVEFSPWQLHPFLKNLQLTNPAGDLVNLNHMRNLEAEYDILNFLNFPYGVGHKVKVGQFSPYVPQSQQLSLSE
NLIEWATGITETPRSVCSESCSPGFRKAPLEGTPTCCFDCTPCSENEISNQTNMDQCVKCPDLQYANTERNQCFHKRVTFLSFEDALGMTLVCTALCFSVL
TVVVLGVFVKHRDTPIVKANNRCLSYVLLITLIFCFLCSLVFIGRPNKTTCVLQQTTFGVVFTVAISTVLAKTITVLLAFRVTGPGRRMRHWLISGLHNSIIPIC
CLIQLALCGFWLGTSPPFIDTDAHSEHGHIILVCNKGSDTTFYCVLGYLGSLALASFTVAFLARNLPDTFNEAKFLTFSMLVFCSVWVTFLPVYHSTKGKVM
VAVEVFSILCSSAGLLGCIFVPKCYIILFRPEKNALK
>rb4 ENSOCUG00000024094
KMFPWIFLLLFLQLYLLVICQDGPKCYSQMERSFYKDGDIEIAAFFPIYIHLVNRLMNIFQRDLTFIRFQSKNYQYVLALVFAIEEINKNPHLLPNMTLGFDLY
NVMHSDMMVMENPFIWLVGMEKHVPNYTCGKQSKSVAIISGTSIAVQMGTLLELYKIPQLTLGSFEPLLSDSGQFPSLYQMAPKDTSLAVGMVSLML
HFSWTWVGLAISDLPKGIQFMSNLKVEMQKNGICVDFVEFIPVTDESRNSLQNRFHIQILKSSANVVILFCDTDSLIGVSFPTWQRVMTWKVWVTTSQ
WDFASDEHHILLHSFHGTLIFSHHHSEISGFKNFLQTVNPSKYPEDFFLSTFWSLHFHCSVTGASCKTLQNCPLNASLESLPFHHFDMSMSDGSYNIYNAV
YAVAHSVHEMLLQELEMQPVSSGSKVEFSPWQLHPFLKNLQLTNPAGDLVNLNHMRNLEAEYDILNFLNFPYGVGRKVKVGQFSPYVPQSQQLSLSEN
LIEWATGITETPRSVCSESCSPGFRKAPLEGKPTCCFDCTPCSENEISNQTNMDQCVKCPDLQYANTERNQCFHKRVTFLSFEDALGMILVCTALCFSVLT
VVVLGVFVKHRDTPIVKANNRYLSYILLIALIFCFLCSLLFIGRPNTATCVLQQTTFGVVFTVAISTVLAKTITVLLAFRVTGPGRRMRQCLISGLPNSIIPICCLI
QLALCGFWLGTSPPFIDTDAHSEHGHIILVCNKGSVTALYCVLGFLGSLALASFTVAFLARNLPDTFNEAKFLTFSMLVFCSVWLTFLPVYHSTKGKVMVA
VEVFSILCSSVALLGCIFVPKCYIILLRPEKNALKGFRDRIISKETDVLRN
>rb5 ENSOCUG00000023660
RWLWKNYQHVLAFHFAIQEINKNPHLLPNMSLGYQFYNGFPSDQFTLWSTLHWLSGQGIFAPNYNCKKQDKSVAIIAGTTATFSAEIGTLLELYKIPQITY
GPFDPLLSDKDRFPSLYQMATSDSSVVHGMISLLLHFGWTWVALFVSDDMKGEQFLRYFETEMVKKGLCVAFTQKLPATKRLYASTDITFMTGISLSSA
NVHILYGEVRSLINVDFAGEYFLTVGKVWIMVAKWNIVVHETYRALHSFHGGFSFSPRKGEIPGLKHFLKTVDPSHYPEDFYFTKLWFHIFTCLPSGSLCGE
IGDCPPNTSLEFSPGDIDIMTLSDSSYFIYNAVYAVAHALHKMFLEKVEMGSLGDAAQPKLLPWQLHPLLKKIQFRNSAGYDISFHETRHDVVQYDIQNIV
NFPVGLGLMVNIGKFFSKSSHDQALVINEEMIEWPIAFKKTPQSVCSQSCGPGFRKVPQEGRPVCCYTCTFCTDRDISNQTDAEQCIPCADHEYPNMER
NRCLPKLVTFLAFEESLGMALACMALCFSVLTAAILWVFVKHRDTPIVKANNRTLSYILLITLLLCFLCSLLFLGRPNTATCLLQQITFGLVFTVAVSTVLAKTI
TVILAFKVTKPGRTIRRLLVSGASNSVIPICFLIQLALCGIWLGTSPPFIDTDAHSEHGHIILVCNKGSATAFYCVLGYLGSLALASFTVAFLARNLPDTFNEAK
FLTFSMLVFCSVWVTFLPVYHSTKGKVMVAVEVFSILASSVGLLGCIFLPKCYIILLRPERNSLKGLKKRASSKGF
>rb6 ENSOCUG00000022887
PDVAPLFRWLWKNYQYVLAFRFAIQEINKDPQLLPNLTLGFQLYNSVTSGQFTLRSTLHWLTGKHHLIPNYTCQTQGKTVAIIAGTTSAFSAEIGTMLELY
KTPQITYGPFDPMLSERDKFPSLYQMATSDSSLAYGMLSLLLHFGWIWVAIFVSNDMKGEQFLQDIEAEMVKKGVCLAYSVKLPDTKSRYEETEISFLEGI
RIASANVHILYGDVRGLYTVEILSKYYLTLGKVWIMATKWEIVVKETDHMLHSFHGGFSFSHHKEEIPGLNHFVKTVDPSHYPEDFFLSQLWLHAFHCLPP
GSLCGTIGVCPPNASFEFFPGRIDMLTISDSSYFIYNAVYAVAHALHKMLSEQIEKSSPGDTSQPSILPWQLHPLLRKIQFTNSAGEDVSFHETRHHMAHY
DIHNIVNFPAGFCFLIKIGEFSPECARGEDLVLNEEMIEWPVAFKETPQSLCSQRCGPGFRKIPQEGKHVCCYTCVVCPERDISNHTADAEQCIPCADHEY
PNMERNRCLPKLVTFLAFEEFLGMALACMALCFSVLTAAVLWVFVKHRDTPIVKANNRTLSYILLIALLLCFLCSLLFLGRPNTATCLLQQIAFGLVFTIAVST
VLAKTITVILAFKALKPGRTIRRLLVSGTSNFIIPICSMIQLVLCGIWLGTSPPFIEVDKHSEHDYIILVCNKGSVTAFYCVLGYLGSLALASFTVAFLARNLPDTF
NEAKFLTFSMLVFCSVWVTFLPVYHSTKGKVMVAVEVFSILASSAGLLGCIFAPKCYVILIKPDKNSLKALRN
>rb7 ENSOCUG00000026847
KSYQYLLAFIFAIEEVNRSPHLLPNVSLGFGLYNTIHSEWKTLESTLTWLSAVGKAIPNYTRVRKSKSVAVITGNTGATSAQIGTLLELYKFPQLTYGPFDQIL
GENHQFTYQMAPRESFLAMGMVSLMLHFHWTWVGLVISEDTRGIQFLWDIREEMNKNEICVAFVEMLSVTDRTYAPSDWHNHFRILESTANVVVIY
GDTNSFMGLSFSRHGLLVTWKVWITTSQWDFITSENEIMMDTFHGTLSFSHSHNEIPGFKYFLKTVNPSKYPENFYLTKFWFLYFPCSISKSDSDMFKAC
PANASLEDLPVHVFDMSMSEGSYHIYNAVYAVAHTLHEMLLQRVEVQPVWNGDGLTVYPWQLNSFLKNIKFDNSVGDEVNLDQERVSRKDYDILNY
WNFPQGLHLQVKVGKFSPRAPHGHRLSISEEMIEWNVEFKEIPRSVCSESCGPGFRKTHQEGQAACCFDCTPCPENEISNETDVEQCVRCLDDQYPNP

ARDRCLPKVVVFLAYEDPLGAALAGVALCLSVLTALVLGVFVKHRNTPLVKANNRNLSYTLLASLCLCFLCSLLFLGRPNTATCILQQTMFAIVFTVAVSTIL
AKTITVVLAFKATAPGKRMRQWLVSGAPNSVIPICSLLQVMLCGIWLGTCPPFIDTDTHAEPQRLLVLCNKGSATLFYLVLGYLGSLALGSFSVAFLARRLP
DTFNEAKYLTFSMLVFCSVWVTFIPVYHSTKGKVMVAVEVFSILASSAGLLGCIFAPKCYILLVRPNRNVLQGIKEKARCS
>rb8 ENSOCUG00000025184
KSYQYLLAFIFAIEEVNRSPHLLPNVSLGFDLYNAIHSEWKTLESTLTWLSAVGKAIPNYTCVRKSKSVAVITGNTGAISAQIGTLLELYKFPQLTYGPFDQILG
ENHQFTYQMAPRESSLAMGMVSLMLHFHWTWVGLVISEDTRGIQFLWDIRQEMNKNEICEAFVEMLSVTDRTYASLEWHYLFRIIESTANVVVIYGD
TNSLMSLSFWRHGLLVTWKVWITTSQWDFITSETEIMMDTFHGTLSFSYSHNEIPGFKHFLKTVNPSKYPEDFYLTKFWFIYFPCSISKADCETLQGCPAN
ASLEDLPVHVFDMSMSEGSYHIYNAVYAVAHTLHEMLLQRVEVQPVWNGDGLTMYPWQLNSFLKNIKFDNSVGDEVNLDQERVSRKDYDILNYWNF
PQGLHLQVKVGKFSPRAPHGHRLSISEEMIEWNVEFKEIPHSVCSESCGPGFRKTHQEGQAACCFDCTPCPEHEISNETDVEQCVRCLDDQYPNPARDR
CLPKVVVFLAYEDPLGAALAGVALGFSVLTALVLVVFVKHRNTPLVKANNRNLSYTLLASLCLCFLCSLLFLGRPNTATCILQQTTFAIMFTVAVSTILAKTIT
VVLAFKATAPGKRMRQWLVSGAPNSVIPICSLLQVMLCGIWLGTCPPFIDTDTHAEPQQLLVLCNKGSATLFYLVLGYLGSLALGSFSVAFLARSLPDTFN
EAKYLTFSMLVFCSVWVTFIPVYHSTKGKVMVAVEVFSILASSAGLLGCIFAPKCYILLVRPNRNVLQGIK
>rb9 ENSOCUG00000026110
RFYSSNYQYVLAFVFAIEEINRNPYLLPNISLGFDLYNALHSEKRTLESCLMWLSGLGKVIPNYSCVREGKSVAVTVGTTWAISAQMGTLLELYKYPQLTYG
SFDPILSDRSQFPSIYQMAPRNTALALGMVRLMLHFGWTWVGLAISEDMKGIQFLWDLTGEMDKNGICVAFVEMIPITERAYFSLAWQYHYRIRDSSA
NVVVIYGDSDSLLGLSFSTWNILMTGKVWITTSQWDFVFSDSYSLLDSFHGTLTFSQSHKEIPGFKQFLQGVNPAKYPQDFYLAKLWFLHFHCLLSETDC
GKLESCPPNVSLESLPLHTFHMTTSDGSYYVYNAVHAVAQTLHELLFHLSEMQSPGHGEKPVVHPWQLHSILKRVQFNNSAGDQIILDQQATPVEEYDIL
NFWNFPDGLGLQVRVGHFVPLAPRDQDFSIHEELIDWAFEFKETPRSLCSKNCGPGFRQSPQEGKAACCFECTPCPEREISNDTDMKQCIKCLDDQYPN
SKRDHCLPKVVTFLAYEDPVGMVLACAALSLSILTAVVLGVFVKHRDTPIVKANNRTLSYILLLSLILCFLCPLFFIGQPNTATCILQQMTFGVLFTVAVSTVL
AKTITVVLAFKSTMPGMKMRQWLLSGAPNSIIPLCSLVQVTFGVVWMVIAPPFIDTDAHSEPTLLIMVCNKGSVIMFYIFLGYLGSLAMGSFTLAFLARSL
PDTFNEAKFLTFSMLVFCSVWIAFLPVYNSTKGKFMVAVEVFSILASSAGLLVCIFAPKCYFILLKSERNILPGSRS
>rb10 ENSOCUG00000027046
YQYVLAFVFAIEEINRNPYLLPNISLGFDLYNALHSEKWTLESCLMWLSGLGKVIPNYSCVREGKSVAVTVGTTWAISAQMGTILELYKYPQLTYGSFDPILS
DRSQFPSIYQMAPRNTALALGMVRLMLHFGWTWVGLAISEDMKGIQFLWDLTGEMDKNGICVAFVEMIPITERAYFSLAWQYHYRIRDSSANVVVIY
GDSDSLLGLSFSKWNILMTGKVWITTSQWDFVFSERYSLLDSFHGTLTFSQSHKEIPGFKQFLQGVNPAKYPQDFYLAKLWFLHFHCLLSETDCGKLESCP
PNVSLESLPLHTFDMTTSDGSYLVYNAVHAVAQTLHELLFQLSEMQSPGHGEKPVVHPWQLHSILKRVQFNNSAGDQIILDQQATPVEEYDILNFWNF
PDGLGLQVRVGHFVPLAPRDQDFSIHEELINWAFEFKETPRSLCSKNCGPGFRQSPQEGKAACCFDCTPCPEREISNDTDMKQCIKCLDDQYPNSKRDH
CLPKVVTFLAYEDPVGMVLACAALSLSILTAVVLGVFVKHRDTPIVKANNRTLSYILLLSLILCFLCPLLFIGQPNTATCILQQMTFGVLFTVAVSTVLAKTVT
VVLAFKSTMPGMKMRQWLLSGAPNSIIPLCSLVQVTFGVVWMVIAPPFIDTDTNSEPTLLIMVCNKGSVIMFYIFLGYLGSLAMGSFTLAFLARSLPDTF
NEAKFLTFSMLVFCSVWIAFLPVYNSTKGKFMVAVEVFSILASSAGLLVCIFAPKCYFILLKS
>rb11 ENSOCUG00000023052
WSHPLPDAAPLFRWLWKNYQYVLAFRFAIQEINKDSQLLPNLTLGFKFFNAFASEQYTLLSIVYWLIGINVPFPNYTCHTQGRHVAVIAGTTSALSVEFGT
LLELYKNPQLTYGPFDPMLNDKDQFPSLYQMATSDSSLGHGMIYLLLHFGWTWVVLFVSDDMKGEQFLQYFEAEMLKKGICVAFTVKLPVTKKLYGYG
DLTFMSSIRVSSANVHILYGDVRGLISVDISVQSFVTIGKVWIMTSKWDLVMSETNHMLHSLHGGFSFSPHREEIPGLKHFVKMANPSHYPEDFYFSKLW
LFHLHCSLEGSFCGHIGSCPPNTSLQFLPGHIDLLTISDSSYLVYNAVYTVAHVLHKMILEKVEMGSPGEAIQPMILPWQLHQFLRKIQFTNSAGDKVSFHE
TRNHMAHYDIQNVVNFPSGLRLLIKIGEFSSKSPHEQSLLVNEEMIEWPVAFKVTPQSVCSQSCGPGFRKMSQEGRPICCYTCVLCPEKEIANQTDAKQC
IPCEDHEYPNQDRSRCLPKLMTFLAFEESLGMALACMALCFSVLTAAVLWVFVKHQDTPIVKANNRTLSYILLITLFLCFLCSLLFLGCPNTATCLLQQIAFG
LVFTVAVSTVLAKTITVILAFKVTKPGRTVRRLLISGVSNSVIPICFLLQLILCSIWLGISPPFIELDTHSEPGHIILVCNKGSVTAFYCVLGYLGSMALASFTVAFL
ARNLPDTFNEAKFLTFSMLVFCSVWVTFLPGYHSTKGKVMVAVEVFSILASSAGLLGCIFVPKCYVILIRPEMNSL
>rb12 ENSOCUG00000023686
LFRWLWKNYQYVLAFRFAIQEINKDPQLLPNLTLGFQLYNSVTSGQFTLRSTLHWLTGKHYLIPNYTCQTQGKTVAIIAGTTSAFLAEIGTMLELYKTPQIT
YGPYDPMLSERDKFPSLYQMATSDNSLICGMLSLLLHFGWIWVAIFVSNDLKGEQFLQDILAEMVKKDVCLAYAIKLPDTKKRYKKTEIYFLAEIRILSANV
HILYGDVRSLYTMEILSKYYLTLGKVWIMAAKWEIVLLETQQMLHSFHGGFSFSHYKEEIPGLKHFVRTVDPSHYPEDFFLSQLWLHAFHCLPPGSLCGTI
GLCPPNASFEFFSGRIDMLTISDSSYFIYNAVYAVAHALHKMLSEQIEKGSPADAAQPRILPWQLHPLLREIQFTNSAGEDVSFHETRFHMAHYDIHNIVN
FPAGFRLLIKIGEFSSESKHDQSLVLNEEMIEWPVAFMETPQSLCSQRCGPGFRKIPQEGRPVCCYTCVVCPERDISNHTDAEQCIPCADHEYPNLERNHC
LPKLVTFLGFEEFLGMALTCMALCFSVLTAAVLWVFVKHRDTPIVKANNRTLSYILLIALLLCFLCSLLFLGRPKTATCLLQQIAFGLVFTVAVSTVLAKTITVIL
AFKVTKPGRTIRRLLVSGASNSVIPICFLIQLALCGIWLGSSPPFIDTDAHSEHGHIILVCNKGSATAFYCVLGYLGSLALASFTVAFLARNLPDTFNEAKFLTF
SMLVFCSVWVTFLPVYHSTKGKVMVAVEVFSILASSAGLLGCIFFPKCYIILLRPERNSLKGLQKRVSSKGS
>rb13 ENSOCUG00000027102
WLWKNYQYVLAFCFAIQEINKDPQLLPNLTLGFQLYNSVTSGQFTLRSTLHWLTGKHYLIPNYTCQTQGKTVAIIAGTTSAFSAEIGTMLELYKTPQITYGP
YDPMLSERDKFPSLYQMATSDNSLICGMLSLLLHFGWIWVAIFVSNDLKGEQFLQDILAEMVKKDVCLAYAIKLPDTKKRYKKTEISFLAEIRISSANVHILY
GDVRSLYTMEILSKYYLTLGKVWIMAAKWEIVLLETQQMLHSFHGGFSFSHYKEEIPGLKHFVRTVDPSHYPEDFFLSQLWLHAFHCIPPGSLCGTIGVCP
PNASFEFFPGHIDMLTISDSSYFIYNAVYAVAHALHKMLSEQIEKGSPADAAQPRILPWQLHPLLREIQFTNSAGEDVSFHETRFHMAHYDIHNIVNFPAG
FRLLIKIGEFSSESKHDQSLVLNEEMIEWPVAFMETPQSLCSQRCGPGFRKIPQEGRPVCCYTCVVCPERDISNHTDAEQCIPCADHEYPNLERNHCLPKL
VTFLGFEEFLGMALTCMALCFSVLTAAVLWVFVKHQDTPIVKANNRTLSYILLIALLLCFLCSLLFLGRPKTATCLLQQIAFGLVFTVAVSTVLAKTITVILAFK

VTKPGRTIRQLLVSGASNSVIPICFLIQLALCGIWLGSSPPFIDTDAHSEHGHIILVCNKGSATAFYCVLGYLGSLALASFTVAFLARNLPDTFNEAKFLTFSML
VFCSVWVTFLPVYHSTKGKVMVAVEVFSILASSVGLLGCIFLPKCYIILLRPERNSLKGLK
>rb14 ENSOCUG00000027252
MLSVSLLCLLLHTIHTLQGMTHRECHHYLKPSINQDGDLILGGFFPLYYPETETEKNYQYVLAFRFAIQEINKDPQLLPNLTLGFKFFNAFASEQYTLLSLVY
WLAGMNLAFPNYSCHKQGRHVAIIAGTTSALSIEFGTLLELYKTPQLTYGPFDPMLNDKDQFPSLYQMATSDSSLGHGMISLLLHFGWMWVALFVSDD
MKGEQFLQYFEAQMLKKSVCVAFRVKLPVTKKLYGDSDLTFMSSIRASSANVHILYGDVRGLIIVDISVRSFLTMGKVWILTSKWDLVMSETNHMLHSL
HGGFSFSPHKEEIPGLKHFVKMANPSHYPEDFYFSKLWLFHLDCSLAGSFCGRIGTCSRNTSLEFLPGHIDLLTISDSSYLVYNAVYTVAHVLHKMLLEKVE
MGSPGEAAQPMILPWQLHPFLRKIQFTNSAGEDVSFHETRNHMVHYYIQNVVNFPSGLRLLIKIGEFSSKNPHEQSLLINEEMIEWPVAFKVTPRSVCS
QSCGPGFRKMSQEGRPICCYTCVLCPEREIANQTDAKQCMQCSDDEYPNQERNRCLPKLVTFLAFEESLGMALACMALCFSVLTAAVLWVFVKHRDTP
IVKANNRTLSYILLITLLLCFLCSLLFLGRPNTATCLLQQIAFGLVFTVALSTVLAKTITVILAFKVTKPGRTMRRLLVSGVYNSVIPICSLIQLALCGIWLGTYPPF
IELDTHSELDHIILVCNKGSATAFYCVLGYLGSLALVSFTVAFLARNLPDTFNEAKFLTFSMLVFCSVWVTFLPVYHSIKGKVMVAVEVFSILASSAGLLGCIF
FPKCYVILIRPEKNTLKGLKKKASSKGF
>rb15 ENSOCUG00000023576
LFRWLWKNYQYVLAFHFAIQEVNKDTHLLPNLSLGFQFYNAIPSDQFTLWNTLYWLSGKNEMIPNYTCQTQEKLVAVIAGTTSAFSAEIGTLLELYGTPQ
LSYGPFDPMLNDKDQFPSLYQMATSDGFLAHGMISLLLHFGWTWIALFVSDDMKGEWFLRDIKAEMVKKGLCVAFEVKLPATKRMFASTDITFMTRL
SVSSANVHILYGDVRSLIHVDIVGQFFLTTGKVWVMAAKWDIVVYETYHMLHSLHGGFSFSPHRGEIPGLKHFLKTVNPSKYPEDFYFSKLWLHIFNCLP
DGSFCGKIGLCPPNASFEFFPGNIDMMTISDSSYFIYNAVYALAHVLHKMLLEKVDILSSGDSSQPMLLPWQLHPPLRKIQFTNNAGDDISFHETRDHMA
HYDIQNIGNFPVGLQLLIKIGEFSSKSPHDQALVISKEMIEWPIAFKETPQSVCSQSCGPGFMKIPQEGKPVCCYSCTFCPERYISNQTDADQCIQCAEHEY
PNSERNRCLSKLVTFLAFEDSLGMALTCMALCCSVITAAVLGVFVKHRHTPIVKANNRSLSYILLISLLLCFLCSLLFIGRPQTATCILQQIMFGLVFTVAVSTV
LAKTITVILAFKVTKPGRTTRQLLISSVSNSVIPICSLIQLIFCGIWLGTSPPFIDRDAHAEHGHIIIVCNKGSVTAFYCVLGYLGFLALASFTVAFLARNLPDTFN
EAKFLTFSMVVFCSVWVTFLPVYHSTKGKIMVAVEVFSILASSAGLLGCIFAPKCYIILIRPERNSLKDFNNRKCSKGS
>rb16 ENSOCUG00000026082
PDVAPLFRWLWKNYQYVLAFRFAIQEINKDPQLLPNLTLGFQLYNSVTSGQFTLRSTLHWLTGKHHLIPNYTCQTQGKMVAIIAGTTSAFSAEIGTMLEL
YKTPQITYGPFDPMLSERDKFPSLYQMATSDSSLACGMLSLLLHFGWIWVAIFVSNDMKGEQFLQDIEAEMVKKGVCLAYSVKLPDTKSRYEETEISFLE
GIRIASANVHILYGDVRGLYTVEFLSKYYLTLGKVWIMATKWEIVVKETDHMLHSFHGGFSFSHHKEEIPGLNHFVKTVDPSHYPEDFFLSQLWLHAFHCL
PPGSLCGTIGVCPPNASFEFFPGRIDMLTISDSSYFIYNAVYAVAHALHKMLSEQIEKSSPGDTSQPSILPWQLHPLLRKIQFTNSAGEDVSFHETRHHMP
HYDIHNIVNFPAGFRLLIKIGEFSPECARGESLVLNEEMIEWPVAFKETPQSLCSQRCGPGFRKMPQEGKHVCCYTCVVCPERDISNHTADAEQCIPCAD
HEYPNMERNRCLPKLVTFLAFEEFLGMALACMALCFSVLTAAVLWVFVKHRDTPIVKANNRTLSYILLIALLLCFLCSLLFLGRPNTATCLLQQIAFGLVFTI
AVSAVLAKTITVILAFKALKPGRTIRRLLVSGTSNFIIPICSMIQLVLCGIWLGTSPPFIEVDKHSEHDYIILVCNKGSVTAFYCVLGYLGSLALASFIVAFLARNL
PDTFNEAKFLTFSMLVFCSVWVTFLPVYHSTKGKVMVAVEVFSILASSAGLLGCIFAPKCYVILIKPDKNSLKALRNQCTCRRP
>rb17 ENSOCUG00000021616
LWKNYQYVLAFHFAIQEINKDPQLLPNLTLGFQLYNSVTSGQFTLRSTLHWLTGNIHFIPNYTCQTQGKTVAIIAGTMSAFSAEIGTMLELYKTPQITYGPF
DPMLSERDKFPSLYQMATSDSSLACGMLSLLLHFGWIWVVIFVSNDMKGEKFLQDIQTEMVKKGLCLANIIKLPDTKSRYEKTEISFLAGLRISSANVHILY
GDVRSVYTMEFLSKYYLTLGKVWIMAAKWEIVVKETDHMLHSFHGGFSFSHHKEEIPGLNHFVKTVDPSHYPEDFFLSQLWLHAFHCLPPGSLCGTIGV
CPPNASFEFFPGRIDMLTISDSSYFIYNAVYAVAHALHKMLSEQIEKGSPGDATQPRIFPWQLHPLLKKIQFTNSAGEDVSFHETRHHMAHYDIHNIVNFP
AGFRLLIKIGEFSSESAHDQGLVLNEEMIEWPIAFKKTPQSLCGQRCGPGFRKMPQEGRALCCYICVICPERDISNHTVDAEQCIPCADHEYPNMERNRCL
PKLVTFLAFEDDLGKALASVALCFSVLTAAVLWVFVKHRDTPIVKANNRTLSYILLITLLLCFLCSLLFLGRPRTATCLLQQIAFCLVFTVAVSTVLAKTITVILAF
KVTKPGRTIRQLLVSGASNFIIPICSLIQVALCVIWLGISPPFIELDAHSEPDHIILVCNKGSATAFHCVLGYLGSLALASFTVAFLARNLPDTFNEAKFLTFSVL
VFCSVWVTFLPVYHSTKGKVMVAVEIFSILASSAGLLGCIFLPKCYIILIKPDKNSLKALKNQRSSRGS
>rb18 ENSOCUG00000025789
LFRWLWKNYQYVLAFRFAIQEINKDPQLLPNLTLGFQLYNSVTSGQFTLRSTLHWLTGKHHLIPNYTCQTQGKTVAIIAGTTSAFLAEIGTMLELYKTPQIT
YGPFDPMLSERDKFPSLYQMATSDSSLAYGMLSLLLHFGWIWVAIFVSNDMKGEQFLQDIEAEMVKKGVCLAYSVKLPDTKSRYEETEISFLEGIRISSAN
VHILYGDVMGLYTMEFLSKYCLTLGKVWIMDTKWEIVVKETDHMLHSFHGGFSFSHHKEEIPGLNHFVKTVDPSHYPEDFFLSQLWLHAFHCLPPGPLC
GTIGVCPPNASFEFFPGRIDMLTISDSSYFIYNAVYAVAHALHKMLSEQMEKRSPGDTSQPRILPWQLHPLLRKIQFTNSAGEDVSFHETRHHMPHYDIH
NIVNFPAGFCLLIKIGEFSPECARGEDLVLNEEMIEWPVAFKETPQSLCSQRCGPGFRKMPQEGKHVCCYTCVVCPERDISNHTADAEQCIPCADHEYPN
MERNRCLPKLVTFLAFEEFLGMALACMALCFSVLTAAVLWVFVKHRDTPIVKANNRTLSYILLITLLLCFLCSLLFLGRPNTATCLLQQIAFGLVFTIAVSTVL
AKTITVILAFKALKPGRTIRWLLVSGTSNFIIPICSLTQLVLCGIWLGTSPPFIEVDKHSEHDYIILVCNKGSVTAFYCVLGYLGSLALASFIVAFLARNLPDTFNE
AKFLTFSMLVFCSVWVTFLPVYHSTKGKVMVAVEVFSILASSAGLLGCIFAPKCYVILIKPDKNSLKALRN
>rb19 ENSOCUG00000023906
FQSKNYQYVLALVFAIEEINKNTHLLPNMTLGFDLYNVMHSDMMVMENPFIWLAGMEKYVPNYTCRKQSKSVAVISGTSIAAQMGTLLELYKIPQLTLG
SFEPLLSDSAQFPSVYQMAPKDTSLAHGMVSLMLHFSWTWVGLAISDLPKGIQFMSDLKVEMQKNGICVDFVEFIPVTEESHNSFQRLYHIQILKSSAN
VVILFCDTDSLIGVSFPTWERVMTWKVWVNTSQWDFASDEHHILLHSFHGTLIFSHHHSEISGFKNFLRTANPSKYPEDIYLLRFWSLVFDCSVTGASCKT
LKNCPLNTSLESLTWHNFDMSMSDGSYNIYNAVYAVAHSVHEMLLQLLEMQPGNKGEKVAFSPWQLHPFLKNLQFTNPAGDLVNLNHLRNLEAEYDI
LNFWNFPYGVGRKVKVGQFSPYASQSQKLSLSENLIEWATGITETPHSVCSESCSPGFRKTPLEGKPTCCFDCTPCPKNEISNQTDMDQCMKCLDHQYA
STERNQCFHKKVTFLSFEDPLGMTLVCTALCFSVLTAVVLGVFVKHRDTPIVKANNRCLSYILLIALIFCFLCSLLFIGHPNTTTCVLQQTTFGVVFTVAVSTIL

AKTITVVLAFKVTAPGRRMRHWLISGVPNSIIPICSLIQLALCAIWLGTSPPFIDTDAHSEHGHIILVCNKGSVTAFYCVLGYLGSLALLSFTVAFLARNLPDTF
NEAKFLTFSMLVFCSVWVTFLPVYHSTKGKVMVAVEVFSILASSAGLLGCIFVPKCYIILFRPEKNALKGLRDRIISKETDVLRSSS
>rb20 ENSOCUG00000024608
LFRWLWKNYQYVLAFRFAIQEINKDPQLLPNLTLGFQLYNSVTSGQFTLRSTLHWLTGKHHLIPNYTCQTQGKTVAIIAGTTSAFSAEIGTMLELYETPQIT
YGPFDPMLSERDKFPSLYQMATSDSSLACGMLSLLLHFGWIWVAIFVSNDMKGEQFLQDIEAEMVKKGVCLAYSVKLPDTKSRYEETEISFLEGIRISSAN
VHILYGDVRGLYTVEFLSKYYLTLGKVWIMATKWEIVVKETDHMLHSFHGGFSFSHHKEEIPGLNHFVKTVDPSHYPEDFFLSQLWLHAFHCLPPGPLCG
TIGVCPPNASFEFFPGRIDMLTISDSSYFIYNAVYAVAHALHKMLSEQMEKRSPGDASQPRILPWQLHPLLRKIQFTNSAGEDVSFHETRHHMPHYDIHN
IVNFPAGFRLLIKIGEFSPECARGESLVLNEEMIEWPVAFKETPQSLCSQRCGPGFRKMPQEGKHVCCYTCVVCPERDISNHTDAEQCIPCADHEYPNME
RNRCLPKLVTFLAFEEFLRMALACMALCFSVLTAAVLWVFVKHRDTPIVKANNRTLSYILLIALLLCFLCSLLFLGRPNTATCLLQQIAFCLVFTVAVSTVLAK
TITVILAFKALKPGRTIRRLLVSGTSNFIIPICSMIQLVLCGIWLGTSPPFIEVDKHSEHDYIILVCNKGSVTAFYCVLGYLGPLALASFTVAFLARNLPDTFNEA
KFLTFSMLVFCSVWVTFLPVCHSTKGKVMVAVEVFSILASSAGLLGCIFAPKCYVILIKPDKNSLKA
>rb21 ENSOCUG00000025636
FQSKNYQYALALVFAIEEINKNPQLLPNVTLGFDLYNVMHSDMMVLQNPFIWLAGLEEDVPNYTCRKQSKSVAVISGTSIAVQVGTLLELYKIPQLTIGSF
EPLLSDSGQFSSLYQMAPKDNSLARGIVSLMLHFSWTWVGLAISDLPKGIQFMSNLKVELQKNGICVDFVELIAVTEESYISLQNRFHIQILKSSANVVILFC
DTDSLIGISFPTWERVMTWKVWVTTSQWDFASFEDPFLLHSFHGTLIFSHHHGEISGFKNFLQTANPSKYPEDFYLLRFWLLVFDCSVTGASCKTLQNCP
LNASLESLPPHRFDMSMSDGSYNIYNAVYAVAHSVHEMLLQEVEMQPVNNGAKVAFSPWQLHPFLKNLQFTNPAGDLVNLNHLRNLEAEYDILNFLN
FPYGVVHKVKVGQFSPYASQSQKLSLSENSIEWATGITETPHSVCSESCSPGFRKTPLEGKPTCCFDCTPCPENEISNQTDMDQCMKCLDHQYASTERN
QCFHKKVTFLSFEDPLGMTLVCTALCFSVLTAVVLGVFVKHRDTPIVKANNRCLSYILLIALIFCFLCSLLFIGHPNTTTCVLQQTTFGVVFTVAVSTILAKTIT
VVLAFKVTAPGRRMRHWLLSGVPNSIIPICSLIQLALCAIWLGTSPPFIDTDAHSEHGHIILVCNKGSITAFYCVLGYLGSLALLSFTVAFLARNLPDTFNEAK
FLTFSMLVFCSVWVTFLPVYHSTKGKVMVAVEVFSILASSAGLLGCIFVPKCYIILFRPEKNALKGLRDRIISKETDVLRSSS
>rb22 ENSOCUG00000024567
FQSKNYQYVLALVFAIEEINKNPYLLPNMTLGFGLYNVMHSHMTVMENPFIWLVGLEKHVPNYTYRKQNKSVAVISESSIAVQMGTLLELYKIPQLTLGP
FEPLLSDSGQFPSLYQLAPKDTSLAHGMASLMLHFGWTWVGLAISDLPKGIQFMSDLKVELQKNGICVDFVELIPATEESLSSFQSTFHIQILKSSANVVIL
FCVTESLIGASFQSWGQLMTWKVWVTTSQWDFASSEKPFLLNSFHGTLIFSHQHSEISGFKNFLQRANPSKYPEDSYLSRFWSLVFNCSVTGTSCKTLQN
CPLNASLESLPPHHFDMSMSDGSYNIYNAVYAVAHSIHEMLLQEVEMQPVNNGAKVAFSPWQLHPFLKNLQFTNPAGDLVNLNHRKNLEAEYDILNF
WNFPYGFGRKVKVGWFSPYVSQSEQLSLSENLIEWATGITETPRSVCSVSCSPGFRKTPLEGKPACCFDCIPCPENEISNQTDMDQCVKCPDLQYANTER
DQCFYKKLTFLSFEEPLGMTLVCTAVCFSVLTAVVLGVFVKHRDTPIVKANNRCLSYILLIALIFCFLCSLLFIGHPNTTTCVLQQTMFGVVFTMAVCTVLAK
TITVLLAFKVTAPGRRMRHWLISGLPNSIIPICCLIQLALCAIWLGTSPPFIDTDAHSEHGHIILVCNKGSVTAFYCVLGYLGSLALASFTVAFLARNLPDKFNE
AKFLTFSMLVFCSVWVTFLPVYHSTKGKVMVAVEVFSILASSVGLLGCIFVPKCYIILFRPEKNALKGFRDRIISKETDVLKCSS
>rb23 ENSOCUP00000025792
FKSKNYQYALALVFAIEEINKNPHLLPNMTLGFGIYNVMHSHITVMENPFIWLVGLEKYVPNYTCKKQSKSVAVISESSIAVQMGTLLELYKIPQLTLGPFE
PLLSDSGQFPSLYQMAPKDSSLARGMVSLMLHFGWTWVGLAISDLPKGIKFMTDLKVELQKNGICVDFVELIPATEEALSSFQSTFHIQILKSSANVVILFC
VTESLIGASFQSWWELMMWKVWVTTSQWDFASSEEPFLFHSFHGTLIFSDHHSEISGFKNFLRTANPSKYPEDSYLSRFWSLAFNCSVTGTSCKTLQNC
PLNASLESLPPHHFDMTMSDGSYNIYNAVYAVAHSIHEMILQEVEMQPVNNGAKVAFSPWQLHPFLKNLQFTNPAGDLVNLNQKRNLEAEYDILNFW
NFPYGFGRKVKVGRFSPHVSQSEQLSLSENLIEWATGITEIPHSVCSVSCSPGFRKTPLEGKPACCFDCTPCPGNEISNQTDMDQCVNCPDLQYANTERD
QCFHKKVTFLSFEDPLGMTLVCTALCFSILTVVFLGMFVKHRDTPIVKANNRCLSYILLIALTFCFLCSLLFIGHPNTTTCVLQQTMFGVAFTAAVSTILAKTIT
VLLAFKFTAPGRRMRHWLLSGVPNSIIPICSLIQLALCAIWLGTSPPFIDTDAHSEHGHIILVCNKGSVTAFYCVLGYLGSLALASFTVAFLARNLPDTFNEAK
FLTFSMLVFCSVWVTFLPVYHSTKGKVMVAVEVFSILCSSAGLLGCIFVPKCYIILFRPEKNALKGFRDRIVSKETDVLKNSS
>rb24 ENSOCUG00000024215
MFPLNFLLLFLQLCLLVTCHDDQNCFFQMERSYYKDGDIEIAAFFPIYIYLVDRMINIFKNHVTFKGFQSKNYQYVLALVFAIEEINKNPQLLPNVTLGFDLY
NVMHSDMMVMQNPFIWLAGLEEDVPNYMCRKWSKSVAVISGTSIAVQVGTLLELYKIPQLTLGSFEPLLSDSGQFPSLYQMAPKDTSLALGIVSLMLH
FSWTWVGLAISDLPKGIQFMSNLKVELQKNGICVDFVELIAVTEESYISLQNRFHIQILKSSANVVILFCDTDSLIGISFPTWERVMTWKVWVTTSQWDFA
SDEHHILLHSFHGTLIFSHHHGEISGFKNFLQTANPSKYPEDIYLLRFWLLVFDCSVTGASCKTLQNCPLNASLESLPPHRFDMSMSDGSYNIYNAVYAVA
HSVHEMLLQLLEIQPVNNGAKLTFSPWQLHPFLKNLQFTNPVGDLVNLNHMTNLEAEYDILNFWNFPYGVGRKVKVGQFSPYASQSQKLSLSENLIEW
AIGITETPRSVCSESCSPGFRKASLEGKPTCCFDCTPCPENEISNQTDMDQCMKCLDHQYASTERNQCFHKKVTFLSFEDPLGMTLVCTALCFSVLTAVVL
GVFVKHRDTPLVKANNRCLSYILLIALIFCFLCSLLFIGHPNTTTCVLQQTTFGVMFTVAVSTVLAKTITVVLAFKVTAPGRRMRHWLISGVPNSIIPICSLIQL
ALCAIWLGNSPPFIDTDAHSEHGHIILMCNKGSVIAFYCVLGYLGSLAMASFTVAFLARNLPDKFNEAKFLTFSMLVFCSVWVTFLPVYHSTKGKVMVAV
EVFSILASGAGLLGCIFVPKCYIILLRPEKNALKGLRDRRISKETDVLRCSS
>rb25 ENSOCUG00000021793
MFPLIFLLLFLQLCLLVTCHDDRNCFFQMERSYYKDGDIEIAAFFPIYIYLVDRMINIFKKDFTFKRFQSKNYQYALALVFAIEEINKNPQLLPNVTLGFDLYN
VMHSDMMVLQNSFIWLAGLEEDVPNYMCRKQSKSVAVISGTSIAVQVGTLLELYKIPQLTLGSFEPLLSDSGQFPSLYQMAPKDTSLAHGLVSLMLHFS
WIWVGLAISDLPKGIQFMSDLKVELQKNGICVDFVELIPVTEESRISLQNRFHIQILKSSANVVILFCDTDSLIGISFPTWERVMTWKVWVTTSQWDFASD
ERHILLHSFHGTLIFSHHHSEISGFKNFLWTANPSKYPEDIYLLRFWSLVFDCSVTGASCKTLRNCPLNTSLELLPLHHFDMSMSDGSYNIYNAVYAVAHSV
HEMLLQLLEMQPGNKGEKVAFSPWQLHPFLKNLQFTNPAGDLVNLNHLRNLEAEYDILNFWNFPYGVGRKVKVGQFSPYASQSQKLSLSENLIEWAT
GITETPHSVCSETCSPGFRKAPLEGKPTCCFDCTPCPENEISNQTDMDQCVKCLDHQYANRERNQCFYKKLTFLSFEEPLGMILVCTALCFSVLTALVLGVF
VKHRDTPIVKANNRCLSYILLIALIFCFLCSLLFIGRPNTTTCVFQHTTFGVVFTVAVSTVLAKTITVLLAFKVTAPGRRMRHWLISGVPNSIIPICSLIQLALCAI

WLGTSPPFIDTDAHSEHGHIILVCNKGSVIAFYCVLGYLGSLALASFSVAFLARSLPGTFNEAKFLTFSMLVFCSVWVTFLPVYHSTKGKVMVAVEVFSILCS
SAGLLGCIFVPKCYIILLRPEKNALKGLRDRRISKETDVLRCSS
>rb26 ENSOCUG00000026621
LSCRFQSKNYQYVLALVFAIEEINKNPHLLPNMTVGFGLYNVMHSHMTVMENPFIWLAGLEEDIPNYTCRKQSKSVAVISESSIAVQMGTLLELYKIPQLT
LGPFEPVLSDSGHFPSLYQMAPKDTSLTHGMASLMLHFGWTWVGLAISALPKGIQFMSDLKVELQKNGICVDFVELIPATLESLSSFQNSVHIQILKSSAN
VVILFCVTESLIGTSFQSWRELIMWKVWVTTSQWDFASSEKPFLFHSFHGTLIFSDHHSEISGFKNFLRTANPSKYPEDSYLSRFWSLAFNCSVTGTSCKTL
QNCPLNASLESLPPHHFDMTMSDGSYNIYNAVYAVAHSIHEMLLQEVEMQPVNNGAKVAFSPWQLHPFLKNLQFTNPAGDLVNLNHRKNLEAEYDIL
NFWNFPYGFGHKVKVGRFSPYVSQSEQLSLSENLIEWATGITETPRSVCSVSCSPGFRKTPLEGKPACCFDCTPCPENEISNQTDMDQCVKCPHLQYANT
ERDQCFYKKLTFLSFEEPLGMTLVCTALCFSVLTAVVLGVFLKHRDTPIVKANNRCLSYILLITLIFCFLCSLLFIGHPNTTTCVLQQTTFGVVFTMAVSTVLAK
TITVLLAFNFTAPGRRMRHWLISGAPNSIIPICSLIQLALCAIWLGTSPPFVDTDAHSEHGHIILVCNKGSVTAFYCVLGYLGSLALASFTVAFLARNLPDKFN
EAKFLTFSMLVFCSVWVTFLPVYHSTKGKVMVAVEVFSILCSSAGLLGCIFVPKCYIILFRPEKNAFKGFRNRIVSKDTDVLKCSS
>rb27 ENSOCUG00000025263
SLCLSCRFQSKNYQYVLALVFAIEEINNNPHLLPNMTLGFDLYNVMHSDMMVMENPFIWLVGMEKHVPNYTCRKQSKSVAIISGTSIAVQMGTLLELYK
IPQLTLGSFEPLLSDSGQFPSLYQMAPKDTSLAVGMVSLMLHFSWTWVGLAISDLPKGIQFMSNLKVEMQKNGICVDFVEFIPVTDESRNSLQNRFHIQI
LKSSANVVILFCDTDSLIGVSFPTWQRVMTWKVWVTTSQWDFASDEHHILLHSFHGTLIFSHHHSEISGFKNFLQTVNPSKYPEDFFLSTFWSLHFHCSV
TGASCKTLQNCPLNASLESLPFHHFDMSMSDGSYNIYNAVYAVAHSVHKMLLQELEMQPVSSGSKVEFSPWQLHPFLKNLQLTNPAGDLVNLNRMRN
LEAEYDILNFLNFPYGVGRKVKVGQFSPYVPQSQQLSLSENLIEWATGITETPRSVCSESCSPGFRKAPLEGKPTCCFDCTPCSENEISNQTNMDQCVKCP
DLQYANTERNKCFHKRVTFLSFEDALGMILVCTALCFSVLTVVVLGVFVKYRDTPIVKANNRCLSYILLITLIFCFLCSLLFIGRPNKTTCVLQQTTFGVVFTVA
ISTVLAKTITVLLAFRVTGPGRRMRHWLISGLPNSIIPICCLIQLALCGFWLGTSPPFIDTDAHSEHGHIILVCNKGSVTALYCVLGFLGSLALASFTVAFLARN
LPDKFNEAKFLTFSMLVFISVWVTFLPVYHSTKGKVMVAVEVFSILCSSAGLLGCIFVPKCYIILLRPEKNALKGFRDRIISKETDVLRNSS
>rb28 ENSOCUG00000026847
KSYQYLLAFIFAIEEVNRSPHLLPNVSLGFGLYNTIHSEWKTLESTLTWLSAVGKAIPNYTRVRKSKSVAVITGNTGATSAQIGTLLELYKFPQLTYGPFDQIL
GENHQFTYQMAPRESFLAMGMVSLMLHFHWTWVGLVISEDTRGIQFLWDIREEMNKNEICVAFVEMLSVTDRTYAPSDWHNHFRILESTANVVVIY
GDTNSFMGLSFSRHGLLVTWKVWITTSQWDFITSENEIMMDTFHGTLSFSHSHNEIPGFKYFLKTVNPSKYPENFYLTKFWFLYFPCSISKSDSDMFKAC
PANASLEDLPVHVFDMSMSEGSYHIYNAVYAVAHTLHEMLLQRVEVQPVWNGDGLTVYPWQLNSFLKNIKFDNSVGDEVNLDQERVSRKDYDILNY
WNFPQGLHLQVKVGKFSPRAPHGHRLSISEEMIEWNVEFKEIPRSVCSESCGPGFRKTHQEGQAACCFDCTPCPENEISNETDVEQCVRCLDDQYPNP
ARDRCLPKVVVFLAYEDPLGAALAGVALCLSVLTALVLGVFVKHRNTPLVKANNRNLSYTLLASLCLCFLCSLLFLGRPNTATCILQQTMFAIVFTVAVSTIL
AKTITVVLAFKATAPGKRMRQWLVSGAPNSVIPICSLLQVMLCGIWLGTCPPFIDTDTHAEPQRLLVLCNKGSATLFYLVLGYLGSLALGSFSVAFLARRLP
DTFNEAKYLTFSMLVFCSVWVTFIPVYHSTKGKVMVAVEVFSILASSAGLLGCIFAPKCYILLVRPNRNVLQGIKEKARCS
>rb29ENSOCUG00000025181
FYSSNYQYVLAFVFAIEEINRNPYLLPNISLGFDLYNALHSEKRTLESCLMWLSGLGKVIPNYSCVREGKSVAVTVGTTWAISAQMGTILELYKYPQLTYGSF
DPILSDRSQFPSIYQMAPRNTALALGMVRLMLHFGWTWVGLAISEDMKGIQFLWDLTGEMDKNGICVAFVEMIPITERAYSSLAWQYHYRIRDSSAN
VVVIYGDSDSLLGLSFSKWNILMTGKVWITTSQWDFVFSERYSLLDSFHGTLTFSQSHKEIPGFKQFLQGVNPAKYPQDFYLAKLWFLHFHCLLSETDCGK
LESCPPNVSLESLPLHTFDMTTSDGSYLVYNAVHAVAQTLHELLFHLSEMQSPGHGEKPVVHPWQLHSILKRVQFNNSAGDQIILDQQATPVEEYDILNF
WNFPDGLGLQVKVGHFVPLAPRDQDFSIHEELIDWAFEFQETPRSLCSKNCGPGFRQSPQEGKAACCFECTPCPEREISNDTDMKQCIKCLDDQYPNSK
RDHCLPKVVTFLAYEDPVGMVLACAALSLSILTAVVLGVFVKHRDTPIVKVNNRTLSYILLLSLILCFLCPLLFIGQPNTATCILQQMTFGVLFTVAVSTVLAK
TVTVVLAFKTTTPGMKMRQWLLSGAPNSIIPLCSLVQVTFGVVWMVIAPPFIDTDTHSEPTLLIMVCNKGSVIMFYIFLGYLGSLAMGSFTLAFLARSLPD
TFNEAKFLTFSMLVFCSVWIPFLPVYNSTKGKFMVAVEVFSILASSAGLLVCIFAPKCYFILLKSERNILPGSRSKATSR
>rb30ENSOCUG00000024710
FYSSNYQYVLAFVFAIEEINRNPYLLPNISLGFDLYNALHSEKRTLESCLMWLSGLGKVIPNYSCVREGKSVAVTVGTTWAISAQMGTILELYKYPQLTYGSF
DPILSDRSQFPSIYQMAPRNTALALGMVRLMLHFGWTWVGLAISEDMKSIQFLWDLTGEMDKNGICVAFVEMIPIIERAYFSLAWQYHYRIRDSSANV
VVIYGDSDSLLGLSFSKWNILMTGKVWITTSQWDFVFSERYSLLDSFHGTLTFSQSHKEIPGFKQFLQGVNPAKYPQDFYLAKLWFLHFNCLLSEIDCGKL
ESCPPNVSLESLPLHTFDMTMSDGSYYVYNAVHAVAQTLHELLFQLSEMQSPGHGEKPVVHPWQLHSILKRVQFNNSAGDQIILDQQATPVEEYDILNF
WNFPDGLGLQVKVGHFVPLAPRDQDFSIHEELIDWAFEFQETPRSLCSKNCGPGFRQSPQEGKAACCFECTPCPEREISNDTDMKQCIKCLDDQYPNSK
RDHCLPKVVTFLAYEDPVGMVLACAALSLSILTAVVLGVFVKHRDTPIVKANNRTLSYILLLSLILCFLCPLLFIGQPNTATCILQQMTFGVLFTVAVSTVLAK
TVTVVLAFKSTMPGMKMRQWLLSGAPNSIIPLCSLVQVTFGVVWMVIAPPFIDTDTHSEPTLLIMVCNKGSVIMFYIFLGYLGSLAMGSFTLAFLARSLP
DTFNEAKFLTFSMLVFCSVWIAFLPVYNSTKGKFMVAVEVFSILASSAGLLVCIFAPKCYFILLKSERNILLGSRSKAKSRQKEAV
>rb31 ENSOCUG00000022413
VSRWGYRVAQGFVFAIEEINRDTHLLPNLTLGFSIRNSGDSVHGALHETLGFLTGQEEPIPNYACGFSPPQAALIGDTRSALSVSMARLLGLYKFPQVSYSS
TLPSLSDKAQFPSFLRTRASDLTSSHAVSQLVLHFRWSWVGILAQDDDFGQQASSLVARELGQAGVCLEFHLHVPSQQSLEKTEALVQKMASCTATVVL
VFLSNSNFQLLLLGLQGVGVSGQVWVSKDVLHLGLALTVPGVSRVLQGSFGLMLQVSQALGFPEFLARLHPSRTPEDAFIERFWEVTFGCKWPPRNSTA
SGSTRLCSGNESLRGHEHPFQEVSKVDVAYSAVYSIAHALQALGDCVHEDGACADPLHFQPWQLLHPLRKVHFKTPDGKEIMFDSNGDLLTKFDILYGQ
KTANGLFRFVHIGVIDPGATSEKRMTVHLMKEDLQVPSSVCSRSCAPGFSQIPRQGAPQCCFDCSPCPEGQFADQRDMKRCLLCPKEQYSSPTRDRCLP
RTETFLAFDEPLGLTLASVVLLLAGLAVLVLGVFLKHSDTPVVRANNRALSYTLLTSLALCALCPLLFLGRPTTATCLLRQTTFAVVFTVAVSSVLAKTLTVVLA
FRAIRPGVRVQVCLGPNASSSVVLTASLVQVVLCGVWLGTSPPFPHRDVASEPSQVVIYCQEGSGVGFYCMLGYLAVLAGATFCMAFLARGLPDAFNET
KFLTFSMLLFCSIWTAFLPLYHSSRGKSTVAVEIFSILASTAGLLGGIFLPKCYIILLKPERNTLACIRHGRQAQQGRGRARSCRVAVSLGPH

>rb33 ENSOCUP00000001995
LFRWLWKNYQYVLAFRFAIQEINKDSQLLPNLTLGFKFFNAFASEQYTLLSIVYWLIGINMPSPNYTCHTQGRHVAVIAGTTSALSVEFGTLLELYKNPQLT
YGPFDPMLNDKDQFPSLYQMATSDSSLGHGMIYLLLHFGWTWVVLFVSDDMKGEQFLQYFEAEMLKKGICVAFTVKLPVTKKLCGYSDLTFMSSIRVS
SANVHILYGDVSGLISVDISVQSFVTVGKVWIMTSKWDLVMSETNHMLHSLHGGFSFSPHREEIPGLKHFVKTANPSHYPEDFYFSKLWLFHLHCSLEGS
FCGHIGSCPPNTSLEFLPGHIDLLTISDSSYLVYNAIYTVAHVLHKMLLEKVEMGSPGEAIQPMILPWQLHQFLRKIQFTNSAGDKVSFHETRNHMAHYDI
QSIVNFPSGLRLLTKIGEFSSKSPHEQSLLFNEEMIEWPVTFKVTPQSVCSQSCGPGFRKMSQEGRPICCYTCVLCPEKEIANQTDAKQCIPCEDHEYPNQ
DRSRCLPKLMTFLAFEESLGMALACMALCFSVLTAAVLWVFVKHQDTPIIKANNRPLSYILLITLLLCFLCSLLFLGRPNTATCLLRQIAFGLVSTVAVSTGLA
KTITVILAFKVTKPGRPVRRLLISGVSNSVIPICFLLQLILCSIWLGTSPPFIELDTHSEPGHIILVCNKGSVTAFYCVLGYLGSLALASFTVAFLARNLPDTFNEA
KFLMFSILVFCSVWVTFLPVYHSTKGKVMVAVEVFSILASSAGLLGCIFVPKCYVILIRPEMNSLKGLKDKGKF
>rb34 ENSOCUG00000027046
YQYVLAFVFAIEEINRNPYLLPNISLGFDLYNALHSEKWTLESCLMWLSGLGKVIPNYSCVREGKSVAVTVGTTWAISAQMGTILELYKYPQLTYGSFDPILS
DRSQFPSIYQMAPRNTALALGMVRLMLHFGWTWVGLAISEDMKGIQFLWDLTGEMDKNGICVAFVEMIPITERAYFSLAWQYHYRIRDSSANVVVIY
GDSDSLLGLSFSKWNILMTGKVWITTSQWDFVFSERYSLLDSFHGTLTFSQSHKEIPGFKQFLQGVNPAKYPQDFYLAKLWFLHFHCLLSETDCGKLESCP
PNVSLESLPLHTFDMTTSDGSYLVYNAVHAVAQTLHELLFQLSEMQSPGHGEKPVVHPWQLHSILKRVQFNNSAGDQIILDQQATPVEEYDILNFWNF
PDGLGLQVRVGHFVPLAPRDQDFSIHEELINWAFEFKETPRSLCSKNCGPGFRQSPQEGKAACCFDCTPCPEREISNDTDMKQCIKCLDDQYPNSKRDH
CLPKVVTFLAYEDPVGMVLACAALSLSILTAVVLGVFVKHRDTPIVKANNRTLSYILLLSLILCFLCPLLFIGQPNTATCILQQMTFGVLFTVAVSTVLAKTVT
VVLAFKSTMPGMKMRQWLLSGAPNSIIPLCSLVQVTFGVVWMVIAPPFIDTDTNSEPTLLIMVCNKGSVIMFYIFLGYLGSLAMGSFTLAFLARSLPDTF
NEAKFLTFSMLVFCSVWIAFLPVYNSTKGKFMVAVEVFSILASSAGLLVCIFAPKCYFILLKS
