# Supplementary material for: A Recent Class of Chemosensory Neurons Developed in Mouse and Rat
Source: PLoS One. 2011 Sep 9;6(9):e24462. doi: 10.1371/journal.pone.0024462 (PMC3170373; doi:10.1371/journal.pone.0024462)
Supplement: Table S1 — Distribution of family-ABD V2Rs in family-C positive neurons. (DOC) [file pone.0024462.s010.doc]

**Table S1. Distribution of family-ABD V2Rs in family-C positive neurons.** Percentage of family-ABD V2R positive neurons expressing Vmn2r1 and Vmn2r2. The number of analysed cells is shown in parenthesis and represents the number of immune-positive family-ABD VNO neurons also reacting with anti-Vmn2r1 or anti-Vmn2r2 antibodies.

For each set of coexpression experiments four 2-3-month old mice were analyzed. Due to the very low level of expression, the analysis of the distribution of subfamily A10 was performed on eight mice.

|  | **A1** | **A2** | **A3** | **A4** | **A5** | **A6** | **A8** | **A9** | **A10** | **Family-B** | **Family-D** |
| --- | --- | --- | --- | --- | --- | --- | --- | --- | --- | --- | --- |
| **Vmn2r1** | **12%**  (38/310) | **9%**  (23/270) | **5%**  (35/291) | **5%**  (10/205) | **1%**  (29/252) | **16%**  (18/113) | **88%**  (627/716) | **98%**  (629/654) | **84%**  (66/79) | **82%**  (311/253) | **79%**  (786/989) |
| **Vmn2r2** | **87%**  (390/488) | **83%**  (713/857) | **86%**  (535/632) | **91%**  (863/945) | **81%**  (74/91) | **73%**  (136/187) | **18%**  (63/360) | **19%**  (75/396) | **64%**  (147/228**)** | **37%**  (72/197) | **19%**  (112/581) |
