# Supplementary material for: A Recent Class of Chemosensory Neurons Developed in Mouse and Rat
Source: PLoS One. 2011 Sep 9;6(9):e24462. doi: 10.1371/journal.pone.0024462 (PMC3170373; doi:10.1371/journal.pone.0024462)
Supplement: Table S2 — Chromosomal organization of family-ABD genes. (DOC) [file pone.0024462.s011.doc]

**Table S2.** **Chromosomal organization of family-ABD genes.**

Chromosomal location and orientation of family-ABD V2Rs (intact genes) and their preferential expression in subfamily-C1 (shaded in dark grey) and subfamily-C2 (shaded in light grey) positive neurons.

|  | **A1** | | | | **A2** | **A3** | **A4** | **A5** | **A6** | **A8** | | **A9** | **A10** | **Fam-B** | **Fam-D** |
| --- | --- | --- | --- | --- | --- | --- | --- | --- | --- | --- | --- | --- | --- | --- | --- |
| **chromosome** | 5 | 10 | 14 | X | 17 | 17 | 7 | 7 | 17 | 7 | 17 | 10 | 10 | 6 | 7 |
| **forward strand** | 2 | 0 | 2 | 0 | 0 | 4 | 4 | 4 | 0 | 3 | 15 | 4 | 0 | 4 | 0 |
| **reverse strand** | 8 | 4 | 0 | 1 | 1 | 3 | 21 | 11 | 1 | 5 | 6 | 0 | 1 | 5 | 4 |
| **total receptors** | **17** | | | | **1** | **7** | **25** | **15** | **1** | **29** | | **4** | **1** | **9** | **4** |
